# *BcMYB111* Responds to BcCBF2 and Induces Flavonol Biosynthesis to Enhance Tolerance under Cold Stress in Non-Heading Chinese Cabbage

**DOI:** 10.3390/ijms24108670

**Published:** 2023-05-12

**Authors:** Xiaoshan Chen, Ying Wu, Zhanghong Yu, Zhanyuan Gao, Qiang Ding, Sayyed Hamad Ahmad Shah, Wenyuan Lin, Ying Li, Xilin Hou

**Affiliations:** 1 State Key Laboratory of Crop Genetics & Germplasm Enhancement and Utilization, Key Laboratory of Biology and Genetic Improvement of Horticultural Crops (East China), Engineering Research Center of Germplasm Enhancement and Utilization of Horticultural Crops, Nanjing Agricultural University, Nanjing 210095, China; 2019204020@njau.edu.cn (X.C.); 2020104052@stu.njau.edu.cn (Y.W.); 2018204024@njau.edu.cn (Z.Y.); 2021204025@stu.njau.edu.cn (Z.G.); 2019204024@njau.edu.cn (Q.D.); 2018204050@njau.edu.cn (S.H.A.S.); 2020104053@stu.njau.edu.cn (W.L.); yingli@njau.edu.cn (Y.L.); 2 Nanjing Suman Plasma Engineering Research Institute Co., Ltd., Nanjing 211162, China

**Keywords:** non-heading Chinese cabbage, flavonols, *BcMYB111*, *BcCBF2*, cold stress

## Abstract

Flavonols have been shown to respond to a variety of abiotic stresses in plants, including cold stress. Higher total flavonoid content was found in non-heading Chinese cabbage (NHCC, *Brassica campestris* (syn. *Brassica rapa*) ssp. *chinensis*) after cold stress. A non-targeted metabolome analysis showed a significant increase in flavonol content, including that of quercetin and kaempferol. Here, we found that an R2R3–MYB transcription factor, *BcMYB111*, may play a role in this process. *BcMYB111* was up-regulated in response to cold treatment, with an accompanying accumulation of flavonols. Then, it was found that BcMYB111 could regulate the synthesis of flavonols by directly binding to the promoters of *BcF3H* and *BcFLS1*. In the transgenic hairy roots of NHCC or stable transgenic *Arabidopsis*, overexpression of *BcMYB111* increased flavonol synthesis and accumulation, while these were reduced in virus-induced gene silencing lines in NHCC. After cold stress, the higher proline content and lower malondialdehyde (MDA) content showed that there was less damage in transgenic *Arabidopsis* than in the wild-type (WT). The *BcMYB111* transgenic lines performed better in terms of antioxidant capacity because of their lower H_2_O_2_ content and higher superoxide dismutase (SOD) and peroxidase (POD) enzyme activities. In addition, a key cold signaling gene, BcCBF2, could specifically bind to the DRE element and activate the expression of *BcMYB111* in vitro and in vivo. The results suggested that *BcMYB111* played a positive role in enhancing the flavonol synthesis and cold tolerance of NHCC. Taken together, these findings reveal that cold stress induces the accumulation of flavonols to increase tolerance via the pathway of BcCBF2–BcMYB111–BcF3H/BcFLS1 in NHCC.

## 1. Introduction

Flavonoids are a group of essential secondary metabolites in plants, and they play an important role in plant physiology. As one of the pigments of plants, flavonoids can affect their coloration [1]. As signaling molecules, flavonoids can also attract insects for pollination and participate in auxin metabolism [2]. Because of their ability to scavenge reactive oxygen, flavonoids can be used as plant antitoxins or antioxidants [3,4]. The biosynthesis of flavonoids in plants could induce the accumulation of flavonoids [5], including during pathogen infection [6], drought stress [7], UV [8], cold stress [9], etc. In addition, flavonoids can delay the aging of the nervous system, improve human immunity, and prevent cancer [10,11].

Flavonoids are C6–C3–C6 polyphenols composed of 15 carbons [3]; they can be divided into flavonols, anthocyanins, flavanones, flavones, isoflavones, and flavonols [12]. A large number of plant flavonoids have been identified [13]. The biosynthesis of flavonoids is mainly controlled by structural and regulatory genes. In recent years, the main structural genes (*CHI*, *CHS*, *F3H*, *FLS*, *DFR*) and regulatory genes (*MYB*s, *bHLH*, *WD40*) related to flavonoid biosynthesis have been widely studied [14,15]. It is known that the MYB family genes are important transcriptional regulators of flavonoid biosynthesis, and they can be divided into 1R–MYB/MYB-related, R2R3–MYB-related, 3R–MYB-related, and 4R–MYB4-related genes [16]. R2R3–MYBs participate in the process of flavonoid synthesis by regulating related genes [17]. CsMYB60 promoted the accumulation of flavonols and proanthocyanidins by binding to the promoters of *CsFLS* and *CsLAR* in cucumbers [18]. In plants of the genus Epimedium, the ectopic expression of *EsMYBF1* led to an increase in flavonols and a decrease in anthocyanin, while EsMYBF1 acted independently of EsTT8 [19]. MdMYB22 [20], MdMYB8 [15], and MdMYB111 [21] were also proven to regulate the biosynthesis of flavonols in apples. In addition, AtMYB4 could inhibit the expression of *AtMYB7* to down-regulate the content of flavonols [22]. In *Arabidopsis thaliana*, it was shown that AtMYB11, AtMYB12, and AtMYB111 had the potential to activate four flavonol biosynthesis genes (*CHS*, *CHI*, *F3H*, and *FLS1*). There were fewer flavonols in the triple-mutant *myb11myb12myb111*, but the accumulation of anthocyanins was unaffected [23]. When influenced by AtMYB11, AtMYB12, and AtMYB111, the spatial accumulation of flavonol derivatives was discrepant in different plant organs. Otherwise, the accumulation of kaempferol derivatives in leaves depended on *AtMYB111* [24].

Cold stress is a common abiotic stress. Plants change their soluble resistant proteins, carbohydrates, and secondary metabolites to cope with cold stress [25,26]. Most flavonols and anthocyanins accumulate when plants face cold stress [27]. As was reported, the expression of flavonoid synthesis genes, including *PAL*, *CHS*, *F3H,* and *FLS1*, could be significantly up-regulated [28,29]. Then, the biosynthesis of flavonoids was promoted [27]. At present, it has been found that low temperatures can significantly induce the accumulation of flavonoids in ginkgo leaves [30], kale [31], *Arabidopsis* [32], and apples [33]. In particular, quercetin and kaempferol increase significantly under cold stress.

Non-heading Chinese cabbage (NHCC) is one of the most popular vegetables in Asia [34]. There is extensive research on the qualities of NHCC, such as its contents of ascorbic acid [35], anthocyanin [36], and aromatic substances [37]. *MYB111* is involved in flavonoid metabolism, and it has been reported to respond to some abiotic stress pathways. However, there are few studies on the regulation of flavonoids in NHCC under cold stress. Therefore, we explored the response mode of *BcMYB111* during cold stress and identified the types of flavonoids by using non-targeted metabolomics. Furthermore, we verified the function of *BcMYB111* and found the upstream regulation of the gene that responded to cold stress. Finally, a regulatory model is proposed, showing that *BcMYB111* responded to cold stress and enhanced the accumulation of flavonols in NHCC.

## 2. Results

### 2.1. Metabolomic Analysis of NHCC in Response to Cold Stress

**Figure 1:**
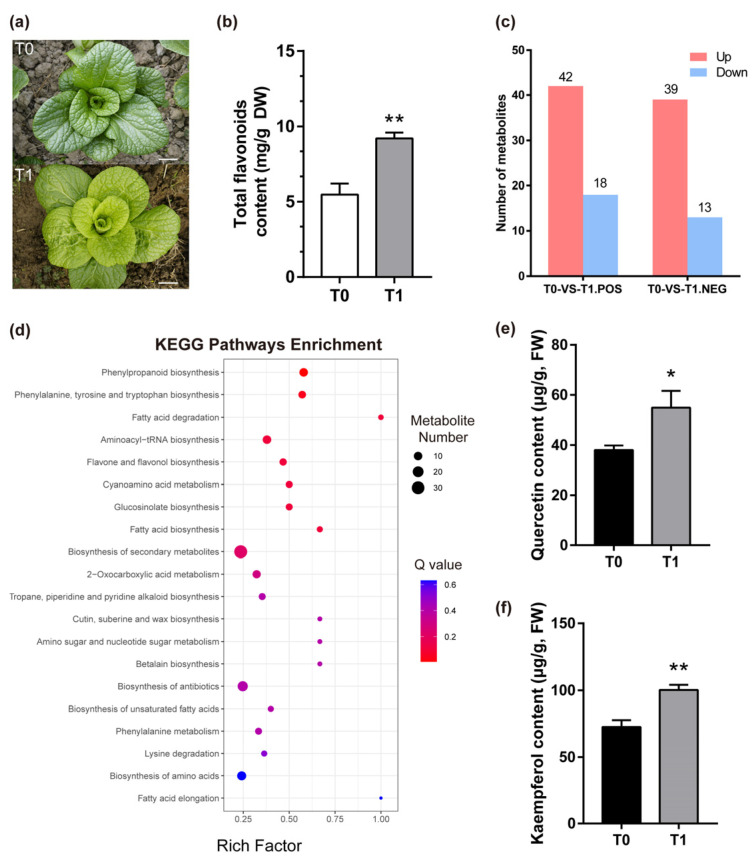
The effect in the flavonoids accumulation under cold stress in NHCC. (**a**) Phenotype observation of NHCC before and after cold stress. T0: before cold stress. T1: after cold stress. Bars: 5 cm. (**b**) Total flavonoid content of T0 and T1 (*n* = 3). DW: dry weight. (**c**) The number of up-regulated and down-regulated metabolites in the POS (positive) and NEG (negative) models between T0 and T1. (**d**) The top 20 significantly enriched KEGG pathways of the differentially expressed metabolites (DEMs) between T0 and T1. Different pathways are represented by the dots, and the gene number and *p*-value are represented by the size and color of the circle, respectively. (**e**,**f**) Alterations of quercetin (**e**) and kaempferol (**f**) content between T0 and T1. All the values represent the mean ± SD of three independent biological replicates (* *p* < 0.05, ** *p* < 0.01, Student’s *t*-test).

As secondary metabolites in plants, flavonoids are generally classified as flavonols, flavonoids, or anthocyanins [13]. After cold stress, there was a significant accumulation of total flavonoids in NHCC (Figure 1a,b). To better understand this biological process, we performed a non-targeted metabolomic analysis. A total of 708 metabolites (327 in the positive ion mode and 381 in the negative ion mode) were identified, and Orthogonal Partial Least Squares–Discriminant Analysis (OPLS–DA) was used to analyze and identify 112 differentially expressed metabolites (DEMs) (Figure S1, Table S1). After exposure to a low temperature, the accumulation of 81 metabolites increased and that of 31 metabolites decreased (Figure 1c). Kyoto Encyclopedia of Genes and Genomes (KEGG) pathway analysis showed that most DEMs were enriched in the pathways of ‘biosynthesis of secondary metabolites’ and ‘biosynthesis of antibiotics’ (Figure 1d). Interestingly, the pathways of ‘phenylpropanoid biosynthesis’, ‘phenylalanine, tyrosine, and tryptophan biosynthesis’, and ‘phenylalanine metabolism’ were also significantly enriched. The results suggested that low-temperature stress had obvious effects on the synthesis and metabolism of phenylalanine-related substances in NHCC. The synthesis of flavonoids begins with phenylalanine, so flavonoids received more attention. Flavonols were highlighted because of their significant accumulation in the process. The contents of quercetin and kaempferol significantly increased after the cold treatment (Figure 1e,f). In a word, these findings showed that the accumulation of flavonols in NHCC was significantly increased after exposure to cold temperatures.

### 2.2. Characterization and Expression Profile of BcMYB111

**Figure 2:**
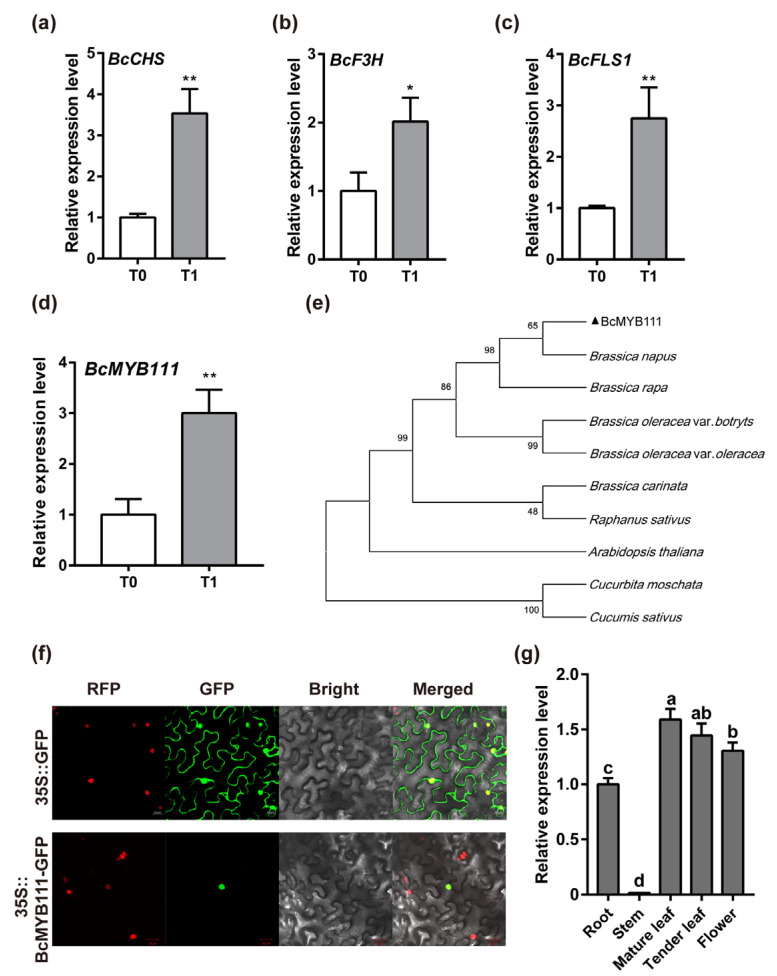
The characterization and expression profile of *BcMYB111* in NHCC. (**a**–**d**) The relative expression of *BcCHS* (**a**), *BcF3H* (**b**), *BcFLS1* (**c**), and *BcMYB111* (**d**) between T0 and T1. The internal control was performed by the expression of *BcGAPC*. Bars indicate the mean ± SD of three repeated experiments (* *p* < 0.05 and ** *p* < 0.01, Student’s *t*-test). (**e**) The phylogenetic relationship of BcMYB111 (highlighted with a black triangle) with other MYB111 proteins in different plants by the neighbor-joining method with 500 bootstrap replicates. The plants used for comparison were *Brassica napus* (XP_048591191.1), *Brassica rapa* (XP_009151728.1), *Brassica oleracea* var. *botrytis* (QCI34366.1), *Brassica oleracea* var. *oleracea* (XP_013594136.1), *Brassica carinata* (KAG2261091.1), *Raphanus sativus* (XP_004134499.1), *Arabidopsis thaliana* (NP_199744.1), *Cucurbita moschata* (XP_022934658.1), and *Cucumis sativus* (XP_018467577.1). (**f**) BcMYB111 is located in the nucleus by subcellular localization analysis. H2B–RFP was used as the nucleus fluorescent protein control. Bars: 20 µm. (**g**) The relative expression of *BcMYB111* in different organs (root, stem, mature leaf, tender leaf, flower) during the flowering period in NHCC. The internal control was performed by the expression of *BcGAPC*. Bars indicate the mean ± SD of three repeated experiments. Different letters indicated statistically significant differences at the level of *p* < 0.05.

Flavonols accumulated during overwintering cultivation, so the synthesis genes were analyzed. The increased expression of *BcCHS*, *BcF3H*, and *BcFLS1* indicated that the synthesis of flavonols was enhanced (Figure 2a–c). The genes of the transcription factor MYB family were always involved in flavonol synthesis. So, we also analyzed the expression of the *MYB* genes in this process (unpublished data, Figure S2). *BcMYB111*, an R2R3-MYB transcription factor, was significantly increased after cold stress (Figure 2d). Notably, *MYB111* was reported to be involved in the positive regulation of flavonol biosynthesis and the response to cold in *Arabidopsis thaliana* leaves [38,39]. As a result, it was very likely that *BcMYB111* could play an important role in the regulation of flavonol synthesis at low temperatures in NHCC.

In order to explore the function of *BcMYB111*, we performed a character analysis. BcMYB111, a protein with 434 amino acids, was used for the phylogenetic relationship analysis (Figure 2e and Figure S3). The results indicated that *Brassica campestris* had the closest relationships with *Brassica napus* and *Brassica rapa*. According to the subcellular localization analysis, it was shown that BcMYB111 functions in the nucleus (Figure 2f). The gene pattern analysis showed that the highest expression levels were in the leaves (Figure 2g). These findings showed that *BcMYB111* mainly functions in the leaves, which may directly respond to cold stress.

### 2.3. Cold Stress Induces the Up-Regulated Expression of BcMYB111 and the Accumulation of Flavonols

**Figure 3:**
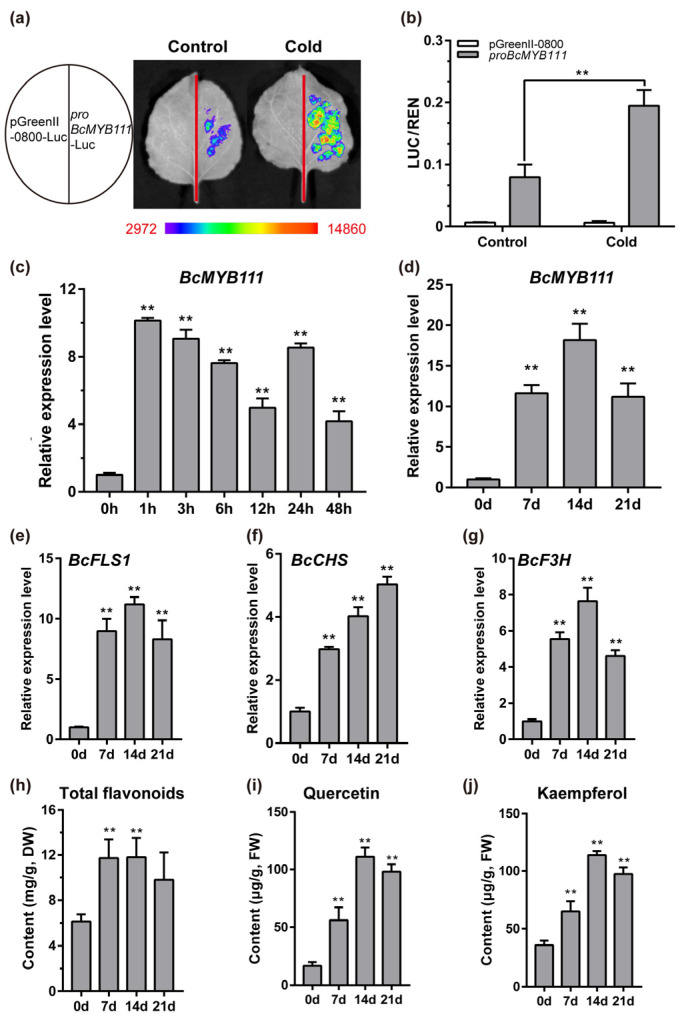
Cold stress induced the up-regulation of *BcMYB111* and the accumulation of flavonols. (**a**) Analysis of the promoter activity in tobacco leaves under control (22 °C for 12 h) and cold (8 °C for 3 h and then 22 °C for 9 h). (**b**) The value of LUC/REN activity. (**c**,**d**) The relative expression of *BcMYB111* in the treatment of 48 h (**c**) and 21 days (**d**) under cold stress (8 °C). (**e**–**g**) The relative expression of *BcFLS1* (**e**), *BcCHS* (**f**), and *BcF3H* (**g**) in the 21-day cold stress. The internal control was performed by the expression of *BcGAPC*. (**h**–**j**) The content of total flavonoids (**h**), quercetin (**i**), and kaempferol (**j**) in the 21-day cold stress. FW, fresh weight. All the bars indicate the mean ± SD of three repeated experiments (** *p* < 0.01, Student’s *t*-test).

*MYB111* was up-regulated when *Arabidopsis thaliana* responded to cold [39]. So, we next analyzed the corresponding model of the response of *BcMYB111* to cold stress. We found that the signal from the *BcMYB111* promoter-driven LUC reporter was significantly enhanced after cold in tobacco (Figure 3a,b). A short-term cold treatment (16 h/8 h, 8 °C) was performed in NHCC, showing that the expression of *BcMYB111* reached its peak after 1 h and became stable at a high level (Figure 3c).

To further explore the response of *BcMYB111* and flavonoid accumulation to cold stress, a 21-day cold treatment was performed with NHCC. As before, *BcMYB111* maintained high expression for a long time and reached the highest level on the 14th day (Figure 3d). At the same time, the expression trends of *BcFLS1*, *BcCHS*, and *BcF3H* were consistent with those of *BcMYB111* (Figure 3e–g). Correspondingly, the total flavonoids and flavonols significantly increased (Figure 3h). The contents of quercetin and kaempferol reached their highest levels on the 14th day (Figure 3i,j). Together, these findings indicated that cold stress induced the up-regulation of *BcMYB111* and the accumulation of flavonols.

### 2.4. BcMYB111 Is a Transcriptional Activator of BcF3H and BcFLS1

**Figure 4:**
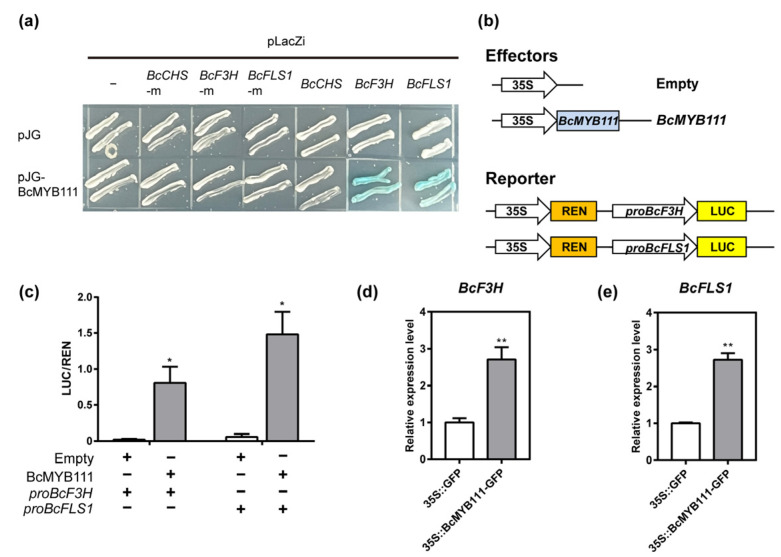
BcMYB111 activates the expression of *BcF3H* and *BcFLS1*. (**a**) Y1H assay for BcMYB111 and the DNA-binding elements of *BcCHS*, *BcF3H*, and *BcFLS1*. Representative image based on three replicates. (**b**) The effectors and reporter constructs used in the dual luciferase assay. (**c**) Measurement of LUC/REN activity in tobacco leaves. (**d**,**e**) The relative expression of *BcF3H* and *BcFLS1* in the transient expression assay. The internal control was performed by the expression of *BcGAPC*. All the bars indicate the mean ± SD of three repeated experiments (* *p* < 0.05, ** *p* < 0.01, Student’s *t*-test).

In *Arabidopsis thaliana*, AtMYB111 has been proven to promote flavonol biosynthesis by interacting with the promoters of *CHS*, *F3H,* and *FLS1* [38]. We speculated that *BcMYB111* may also be a regulatory gene for flavonol synthesis in the cold response of NHCC. After we analyzed the promoter region, the specific DNA-binding elements of BcMYB111 were found in *BcCHS* (CTTAGTTGTTGCGA), *BcF3H* (GGTCCCAGGTAGCT), and *BcFLS1* (TGTGGTAGTTACTT). Then, the mutated sequences were sequenced; these included *BcCHS*-m (CTTAAAAAAAGCGA), *BcF3H*-m (GGAAAAAAATAGCT), and *BcFLS1*-m (TGTAAAAAAACTT). The results of the yeast one-hybrid (Y1H) assay indicated that BcMYB111 is bound to the promoters of *BcF3H* and *BcFLS1* (Figure 4a). There was no interaction between BcMYB111 and the mutated cis-element. A dual luciferase assay was performed on tobacco leaves. The ratio of firefly luciferase to renilla luciferase (LUC/REN) showed a significant increase when 35S::BcMYB111–GFP was added (Figure 4b,c). In addition, a transient expression assay was performed in the cotyledon of NHCC. After overexpression of *BcMYB111*, the expression of *BcF3H* and *BcFLS1* was significantly up-regulated compared with the control (Figure 4d,e and Figure S4). These results indicated that *BcMYB111* could positively regulate *BcF3H* and *BcFLS1* in NHCC.

### 2.5. BcMYB111 Promotes the Accumulation of Flavonols

**Figure 5:**
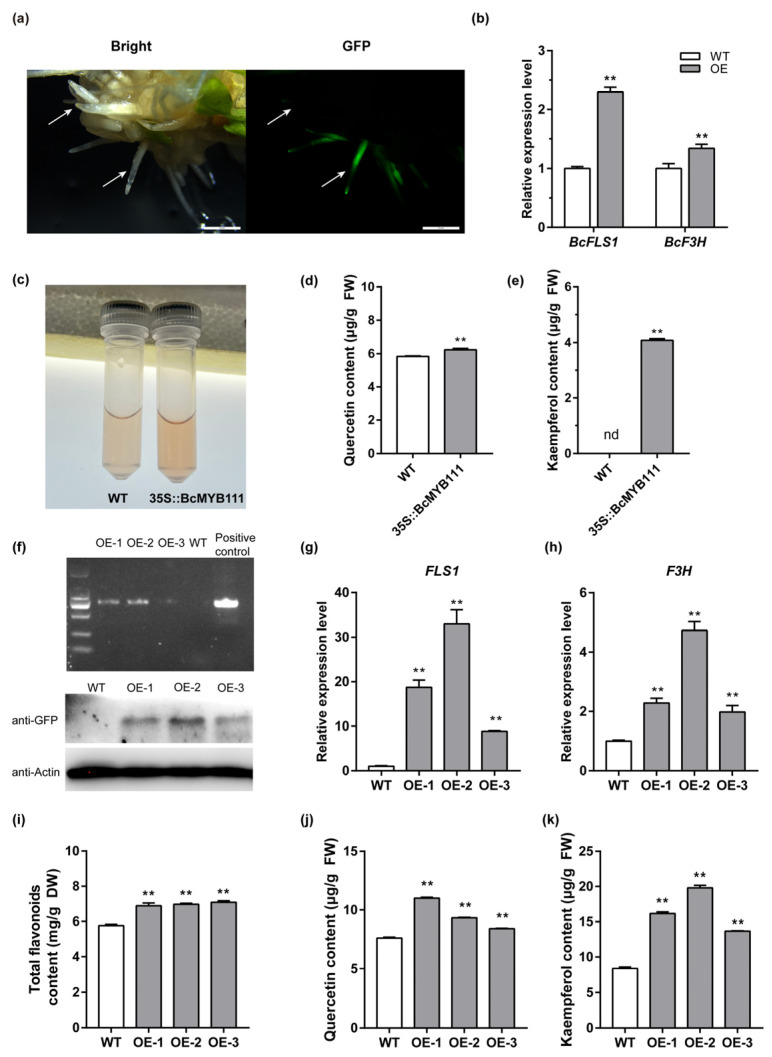
BcMYB111 promotes the accumulation of flavonols in NHCC and *Arabidopsis*. (**a**) The GFP observation of transgenic hairy roots by a fluorescence microscope. Bar: 2 mm. (**b**) The relative expression of *BcF3H* and *BcFLS1* between wild-type (WT) and transgenic hairy roots. (**c**) The phenotype of flavonol content after hydrolysis between WT and transgenic hairy roots. (**d**,**e**) Measurement of quercetin (**d**) and kaempferol (**e**) content between WT and transgenic hairy roots (n = 3). ‘nd’ is marked for ‘not detected’, because the content was below the detection limit. (**f**) The identification of overexpression lines of *Arabidopsis* by PCR and western blot. ‘BcMYB111-DL-F’ and ‘GFP-R’ were used for the PCR to identify the strip (primer Information was provided in Table S2). (**g**,**h**) The relative expression of *FLS1* and *F3H* in WT and overexpression lines. The internal control was performed by the expression of *AtACTIN*. (**i**–**k**) Measurement of total flavonoids (**i**), quercetin (**j**), and kaempferol (**k**) content. All the bars indicate the mean ± SD of three repeated experiments (** *p* < 0.01, Student’s *t*-test).

In order to characterize the function of *BcMYB111* in NHCC, it was constitutively overexpressed in the roots of the ‘Suzhouqing’ variety. Overexpressed roots were generated by using *Agrobacterium rhizogenes* MSU440 and identified via GFP (Figure 5a). The transgenic roots showed no obvious physical abnormalities, but we found that *FLS1* and *BcF3H* were up-regulated in the transgenic roots compared with the wild-type roots (Figure 5b). When we quantified the flavonol content, we found that there was a darker color in the overexpressed root extracts after hydrolysis (Figure 5c) and an increased content of quercetin and kaempferol (Figure 5d,e).

Next, the *BcMYB111* gene was ectopically expressed in *Arabidopsis thaliana* to establish the gene function of *BcMYB111* in the leaves (Figure S4). Three lines (OE-1, OE-2, and OE-3) were verified by using PCR and Western blotting (Figure 5f). The transcription levels of the *BcMYB111*-downstream genes, *FLS* and *F3H*, were also up-regulated in the *BcMYB111* overexpressors (Figure 5g,h). There were significant increases in the contents of total flavonoids, quercetin, and kaempferol (Figure 5i–k). Overall, these results showed that *BcMYB111* could positively regulate the accumulation of flavonols.

### 2.6. The Silencing of BcMYB111 Decreases the Synthesis of Flavonols

**Figure 6:**
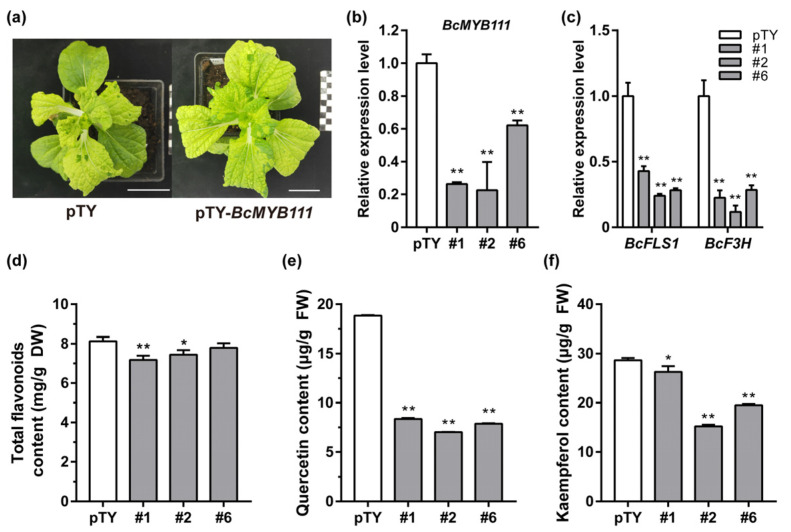
The silencing of *BcMYB111* decreases the biosynthesis of the flavonols and tolerance to cold stress. (**a**) Virus symptoms in the plants. Bar: 5 cm. (**b**) *BcMYB111* expression detection in silenced plants. (**c**) The relative expression of *BcFLS1* and *BcF3H* in silenced plants. The internal control was performed by the expression of *BcGAPC*. (**d**–**f**) Measurement of total flavonoids (**d**), quercetin (**e**), and kaempferol (**f**) content in silenced plants. All the bars indicate the mean ± SD of three repeated experiments (* *p* < 0.05, ** *p* < 0.01, Student’s *t*-test).

To further explore whether *BcMYB111* was needed in flavonol synthesis, silenced lines (pTY–*BcMYB111*) were created by using virus-induced gene silencing (VIGS) in NHCC (Figure 6a,b). The plants injected with an empty pTY vector were used as a control (pTY). The transcription data showed a significant decrease in the expression of *BcFLS1* and *BcF3H* (Figure 6c). The total flavonoid content also slightly decreased (Figure 6d). The accumulation of quercetin and kaempferol was substantially reduced (Figure 6e,f). These observations indicated that *BcMYB111* is critical for the synthesis of flavonols in NHCC.

### 2.7. BcMYB111 Improves Cold Tolerance

**Figure 7:**
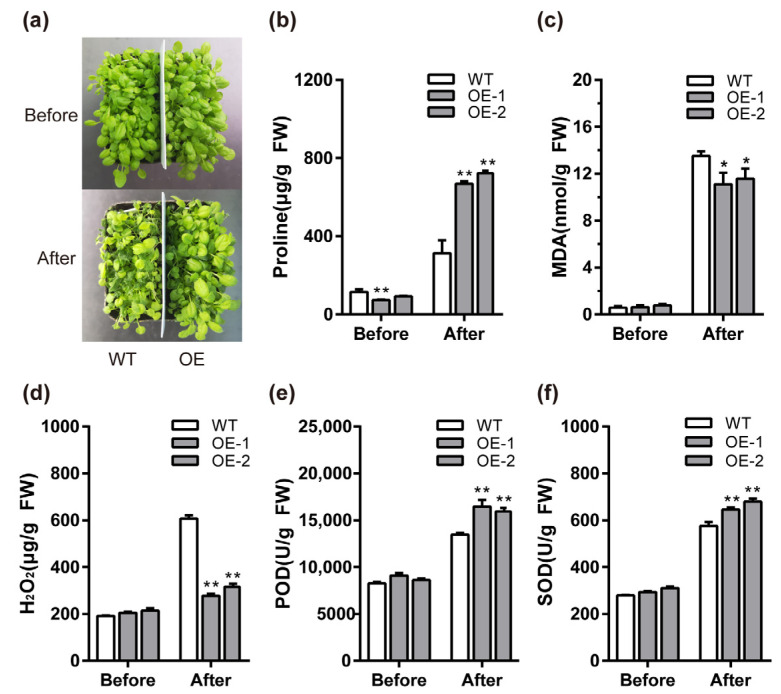
Overexpression of BcMYB111 increased cold tolerance in *Arabidopsis thaliana*. (**a**) Phenotypes of *Arabidopsis thaliana* before and after cold treatment. The plants were moved to a −5 °C chamber for 24 h, another chamber for a suitable temperature for recovery, and observed as ‘After’. (**b**–**d**) Content assay of proline (**b**), MDA (**c**), and H_2_O_2_ (**d**) in WT and overexpressing lines before and after cold treatment. (**e**,**f**) Activity of POD (**e**) and SOD (**f**) in *Arabidopsis thaliana* seedlings from WT and transgenic lines. All the bars indicate the mean ± SD of three repeated experiments (* *p* < 0.05, ** *p* < 0.01, Student’s *t*-test).

As antioxidants, flavonols often exhibit positive performance in plants’ adaptation to stress. It is worth studying whether the overexpression lines of *BcMYB111* perform better because of the higher flavonol content under cold stress. *BcMYB111* transgenic *Arabidopsis* (OE) and the wild type (WT) were exposed to cold. The WT plants were more sensitive to cold than the overexpressing lines (Figure 7a). Compared with the WT, the overexpressing lines contained more proline, suggesting that the transgenic lines had better resistance to cold (Figure 7b). In terms of plant peroxidation damage, there were lower MDA and H_2_O_2_ contents in the overexpressing lines than in the WT (Figure 7c,d). In the meantime, the increase in POD and SOD enzyme activities in the overexpressing lines suggested that the transformed plants suffered less damage during the cold treatment (Figure 7e,f). Taken together, these data demonstrate that the overexpression of *BcMYB111* could increase the tolerance to cold stress.

### 2.8. BcMYB111 Is a Direct Target Gene of BcCBF2

**Figure 8:**
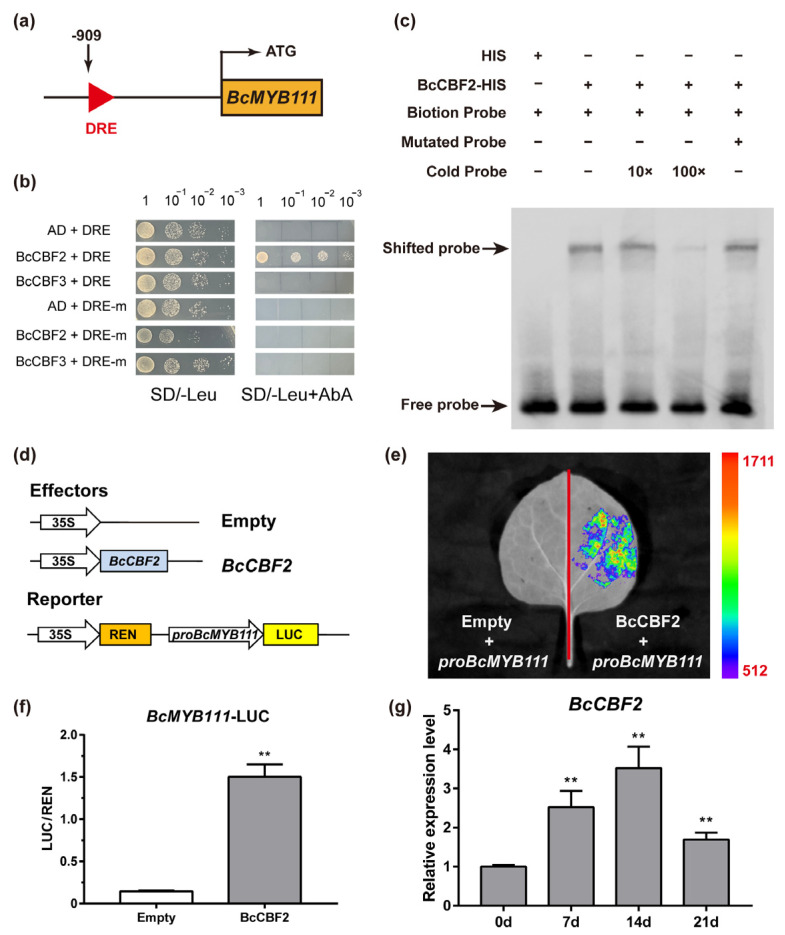
BcCBF2 activates the expression of *BcMYB111* under cold stress. (**a**) Diagram of the *BcMYB111* promoter. The DRE element is marked with a red triangle. (**b**) Growth of yeast cells on the SD medium lacking leucine (SD/-Leu) with or without Aureobasidin A (AbA) for the Y1H assay. The left shows the various combinations of bait and prey. (**c**) The electrophoretic mobility shift assay (EMSA) of BcCBF2–HIS and DRE elements from the promoter of *BcMYB111*. −: absence; +: presence. (**d**) The effectors and reporter constructs used for the dual luciferase assay. (**e**) Analysis of the LUC signal of the *BcMYB111* promoter in tobacco leaves. (**f**) The value of LUC/REN activity. (**g**) The relative expression of BcCBF2 in the NHCC under the 21-day cold treatment (8 °C). All the bars indicate the mean ± SD of three repeated experiments (** *p* < 0.01, Student’s *t*-test).

The induced expression of CBFs at low temperatures triggers the cold-response transcriptional cascade, and the CRT/DRE element in the promoter of the target genes is an important activation binding site for CBF proteins [40,41]. After scanning, we found the DRE element in the promoter sequence of *BcMYB111* (Figure 8a). In the Y1H assays, BcCBF2 could bind the promoter of *BcMYB111*, while BcCBF3 could not (Figure 8b). There were also no interactions between BcCBF2 and the mutation promoter of *BcMYB111*. After the BcMYB111–His fusion protein was extracted, the EMSA demonstrated that BcCBF2 could bind to the probe with the DRE element of *BcMYB111* and cause a mobility shift (Figure 8c). Moreover, BcCBF2 could not bind to the mutated element (AAAAAA), suggesting that BcCBF2 could specifically bind to the DRE element (GCCGAC) of *BcMYB111*’s promoter. In the dual luciferase assay, the ratio of LUC/REN significantly increased after BcCBF2–GFP was added (Figure 8d–f). In addition, we checked the expression levels of *BcCBF2* during the 21-day cold treatment in NHCC. Our conjecture was further proven because the expression trend was the same between *BcCBF2* and *BcMYB111* (Figure 8g). These results demonstrate that the BcCBF2 protein directly binds and promotes the expression of *BcMYB111* to enhance flavonol synthesis under cold stress.

## 3. Discussion

The MYB transcription factor family is one of the largest gene families in plants. It plays an important role in many biological processes, such as plant growth and development, primary and secondary metabolic responses, and responses to environmental stress [42]. It was proven that *MYB111* could regulate flavonol metabolism in *Arabidopsis thaliana* [23,43]. However, there have been few studies on the regulation of flavonoid metabolism by *MYB111* under cold stress. The central gene, *BcMYB111,* was found to respond to cold stress. *AtMYB111* and the homologous gene *AtMYB12* were proven to be positive regulators of salt stress by regulating the key enzyme genes for flavonoid synthesis [38,44]. Our findings showed that *BcMYB111* could participate in the response to cold stress in NHCC (Figure 3a–d).

There are many kinds of flavonoids [45]. As an important class of flavonoid compounds, the biosynthesis of flavonols is located downstream from the flavonoid pathway, which usually exists in the form of glycosylation in plants [46]. Flavonol synthase (FLS) is a key rate-limiting enzyme in flavonol biosynthesis, and it can convert dihydroflavonols into kaempferol, quercetin, and myricetin in a Freesia hybrid [47]. *MYB111* was proven to promote flavonol biosynthesis in many species, but the structural genes regulating flavonoid metabolism were different in different species. MYB111 could activate the expression of *CHI* and *F3′5′H* in the pomegranate pericarp [48]. However, *MYB111* could interact with the promoters of *CHS* (GAGTTGTTGG), *F3H* (TGGTAGGTA), and *FLS1* (TGGTAGTTG) in *Arabidopsis* [38]. In NHCC, *BcMYB111* could bind the promoters of *BcF3H* (GGTCCCAGGTAGCT) and *BcFLS1* (TGTGGTAGTTACTTC) to regulate flavonol biosynthesis (Figure 4a,c). With regard to the MYB family, more and more studies have shown that MYBs are the main regulators of flavonoid biosynthesis in different species [49,50]. With the exception of that of flavonols, the synthesis pathways of anthocyanin and proanthocyanidins were also regulated by the regulation of *AtMYB114*, *AtMYB113*, *AtMYB90*, and *AtMYB75* [42,51]. In NHCC, we identified a large number of flavonol metabolites that were up-regulated during cold stress through a non-targeted metabolic analysis (Figure 1c,d). These flavonols were affected by low temperatures and were promoted by *BcMYB111* (Figure 3i,j).

Cold stress can affect plant growth, development, and yield [52]. After a long evolution, plants have developed complex biochemical and physiological mechanisms to adapt to cold stress, and these are regulated by a series of transcription factors [53]. There are three main cold-response genes in plants: the inducer of CBF expression (*ICE*), the C-repeat binding factor (*CBF*), and the cold-regulated protein (*COR*). So far, the ICE–CBF–COR signaling pathways have been found in many plants to regulate their adaptation to cold stress [54,55]. CBF, which is also known as the *DREB1* gene, usually induces expression by binding to the cold and dehydration regulatory elements (CRT/DRE) in the promoter of the *COR* genes [56,57]. As a negative regulator of *CBF1* and *CBF3*, *CBF2* is more important in improving plant cold tolerance [58]. In this study, we found that there was a core DRE element (GCCGAC) in the promoter of *BcMYB111*. It was determined that the transcriptional activity of the *BcMYB111* promoter could be activated by cold stress. *BcCBF2* was found to be upstream from *BcMYB111* and able to bind the promoter in vivo and in vitro (Figure 8b,c,e,f). Then, BcCBF2 activated the expression of *BcMYB111* to increase the accumulation of flavonols in NHCC.

In plants, the genes of the MYB family are widely involved in the response to environmental factors and play an important regulatory role in the response to stress [59]. In response to drought, *AtMYB60* improved the drought tolerance of *Arabidopsis thaliana* by regulating the constitution of the stomatal opening [60]. By controlling cell expansion and cuticle deposition, *AtMYB41* was used to cope with abiotic stresses such as ABA, drought, and salt stress [61]. In terms of improving the tolerance of tall plants to drought and salt stress, *GmMYB118* could adjust the osmotic and oxidizing substances in soybean, while *ZmMYB3R* could improve the accumulation of ABA in maize [62,63]. In addition to activating the biosynthesis of the secondary cell wall, *MdMYB46* could also activate the stress response signal, thereby enhancing the tolerance of apples to salt and penetration [64]. MYBs were also involved in the regulation of plant tolerance to cold stress in different modes. By integrating ABA-dependent and ABA-independent signals, the *MYB96*–*HHP* module activated the CBF pathway and enhanced plants’ adaptability to environmental fluctuations [65]. However, *AtMYB14* and *AtMYB15* participated in the cold response by negatively regulating the expression of the CBFs [66,67]. In this study, the expression of *BcF3H* and *BcFLS1* in the hairy roots and *Arabidopsis* leaves of the *BcMYB111* lines was significantly higher than that in the wild-type lines (Figure 5b,g,h). As a result, the total amount of flavonoids and the contents of quercetin and kaempferol were significantly higher than those of the wild type (Figure 5d,e,j,k); thus, they may play a positive role in improving the tolerance of plants to cold temperatures. The relevant indicator of reactive oxygen species (ROS) reflected the sensitivity of plants to abiotic stress [68,69]. The antioxidant capacity of flavonoids can reduce oxidative damage to plants caused by ROS. The phenotype of *BcMYB111*-overexpressing lines under cold stress confirmed our hypothesis. Higher antioxidant capacity was found in the overexpressing lines compared with the WT in *Arabidopsis thaliana* (Figure 7b–f). Research has shown that quercetin and kaempferol can achieve protective effects by clearing reactive oxygen species when exposed to ultraviolet radiation [70]. Cold stress could lead to a significant accumulation of ROS, which is harmful to plants. The stronger cold resistance of *BcMYB111* overexpressing lines was likely caused by the increase in quercetin and kaempferol. These flavonols largely inhibit the accumulation of ROS, which is consistent with other reports [71]. In addition, flavonoids could also enhance plants’ stress resistance by regulating the response and transportation of plant hormones [1,72]. It remains to be explored in more detail whether *BcMYB111* could enhance cold resistance by regulating the synthesis, metabolism, and transportation of different kinds of hormones. In summary, *BcMYB111* played a positive role in the cold resistance of NHCC; this is significant and can be used as a reference for revealing the metabolic network pathway of the MYB family when regulating plants that are under stress. This study revealed one of the functional modes of flavonols in response to low temperatures and showed an improvement in the cold tolerance of NHCC, which provides an important perspective for improving the selection of quality and variety of horticultural plants. Unfortunately, except for the up-regulation of flavonol biosynthesis, we still do not know whether *BcMYB111* improves cold tolerance via a hormonal pathway, the protection of cell membranes and proteins, or other pathways. Further research is needed in this area.

## 4. Materials and Methods

### 4.1. Plant Materials, Growth Conditions, and Treatments

The seedlings of inbred lines (*Brassica rapa* ssp. *Chinensis* cv. NHCC-084-2) were grown in pots containing a soil mix (1:1, vermiculite: humus) in a growth chamber (Ningbo Southeast Instrument Co., Ltd., Ningbo, China), which was set for a daily cycle of 16 h of light (μmol·m^−2^·s^−1^) and 8 h of dark, with 65% humidity and at 22 °C. After the four-leaf stage, the seedlings were moved to 8 °C for the cold-stress treatment. Then, their leaves were sampled at various points for treatments that lasted 48 h and 21 days. In addition, the plants used for the untargeted metabolomic analysis were grown in natural field conditions in the autumn.

*Nicotiana benthamiana* seeds were grown in a 22 °C/19 °C, 75% humidity, and 16 h/8 h light/dark cycle climate chamber. After the disinfection process, *Arabidopsis thaliana* and transgenic seedlings were placed in the same chamber. The State Key Laboratory of Crop Genetics and Germplasm Enhancement of Nanjing Agricultural University provided all of the plant materials.

### 4.2. Untargeted Metabolomic Analysis

The NHCC for the untargeted metabolomic analysis was grown in the city of Jurong. The samples were collected on 23 November 2019, and they were referred to as T0 (18–22 °C), while T1 (8–12 °C) was used to refer to the samples collected on 3 December 2019. The third fully expanded leaf was sampled from the center. Six duplicate samples of independent plants were immediately frozen in liquid nitrogen and stored at −80 °C. Then, 300 μL of methanol and an internal standard were added to the 100 μL samples. After being vortexed and homogenized, the mixture was sonicated for 5 min in an ice-water bath. This was followed by incubation at −20 °C for 2 h and centrifugation for 15 min. The 200 μL samples of the supernatants were transferred to liquid chromatograph–mass spectrometer (LC−MS) vials for an LC−MS analysis. The identification and quantification of the metabolites were performed with the assistance of Genedenovo Biotechnology Co., Ltd. (Guangzhou, China). In particular, the online Kyoto Encyclopedia of Genes and Genomes (KEGG) database was used for annotation. The metabolomic variability between the groups of samples was distinguished by using partial least squares discriminant analysis (PLS–DA). The *p*-value of the Student’s *t*-test and the variable importance in the projection (VIP) were used to screen the differential metabolites (*p* < 0.5 and *VIP* ≥ 0.8).

### 4.3. Subcellular Localization and the Assay of Transient Expression in the Cotyledon of NHCC

The coding region of *BcMYB111* was amplified and inserted into a pCAMBIA1302–GFP vector by using a ClonExpress II One-Step Cloning Kit (C112-01, Vazyme, Nanjing, China) to generate *BcMYB111*–GFP. The recombinant plasmid was introduced into Agrobacterium tumefaciens strain GV3101. Then, the positive agrobacteria of *BcMYB111*–GFP and pCAMBIA1302–GFP were resuspended in an infiltration buffer (10 mM MES, 10 mM MgCl_2_, 150 μM acetosyringone) to inject the leaves of 30-day-old tobacco and the cotyledons of 7-day-old NHCC (Figure S4). About 48 h after the injection, the tobacco leaves were observed to identify the subcellular localization by using confocal laser scanning microscopy (LSM780, Zeiss, Oberkochen, Germany). The cotyledons were collected for quantitative real-time PCR analysis.

### 4.4. Quantitative Real-Time PCR Analysis

The total RNA of NHCC was extracted using the RNAsimple Total RNA Kit (TIANGEN, Beijing, China) and reverse-transcribed using the Hifair^®^ III 1st stand cDNA synthesis superMIX for qPCR (11141ES60, Yeasen, Shanghai, China). Real-time quantification PCR was performed by using the Hieff^®^ qPCR SYBR Green Master Mix (11202ES03, Yeasen, Shanghai, China) from QuantStudio 5 (ABI, San Francisco, CA, USA). The PCR conditions were as follows: predenaturation, 95 °C for 5 min, 1 cycle; for two-step PCR, the conditions were as follows: 95 °C for 10 s, 60 °C for 30 s, 40 cycles. *BcGAPC* and *AtActin* were chosen as the internal standards. The data were analyzed using the 2^−ΔΔCt^ method. There were three biologically independent repetitions in the experiments. All of the primer information is provided in Table S2.

### 4.5. Measurement of the Total Flavonoid, Quercetin, and Kaempferol Contents

After being dried, pulverized, and passed through a 40-mesh sieve, the 0.02 g dry samples were mixed with 2 mL of 60% alcohol (*v*:*v*) and shocked for 2 h at 60 °C. Then, the mixtures were centrifuged at 10,000× *g* for 10 min. After the treatment (Suzhou Comin Biotechnology Co., Ltd., Suzhou, China), the absorbance value of the supernatant at 510 nm was determined by using a Microplate Reader (CYTATION3, Bio Tek, VT, USA). The standard curve was the following: y (mg/g, DW) = 5.02 × ΔA_510_ + 0.0007, R^2^ = 0.9996.

The flavonols in NHCC were determined according to the following method [39]: 0.5 g of leaves were taken; the samples were ground into a powder with liquid nitrogen and mixed with 1 mL of 80% methanol (*v*:*v*) and left overnight at 4 °C; then, centrifugation was performed for 20 min at 4 °C with 12,000 r·min^−1^, and the centrifuged supernatant was filtered with a 0.22 µm organic filter. The filtrate was hydrolyzed by an equivalent of 6 mol/L hydrochloric acid for 40 min at 70 °C. Then, the contents of quercetin and kaempferol were determined with ultra-high-performance liquid chromatography (HPLC, Waters, MA, USA). The injection was 2 μL and the detection wavelength was 360 nm. The standard substances of the flavonoids included quercetin and kaempferol (Yuanye, Shanghai, China).

### 4.6. Dual Luciferase Assay

The promoters of *BcMYB111*, *BcF3H*, and *BcFLS1* were cloned and inserted into the pGreenII–0800–LUC vector. The ORFs of *BcCBF2* and *BcMYB111* were inserted into the pRI101 vector to generate 35S::BcBcCBF2 and 35S::BcMYB111. The constructed vector and empty vector (control) were transformed into GV3101 (including pSoup-19). After injecting different combinations of agrobacterium solution (agrobacteria resuspended with 10 mM MES, 10 mM MgCl_2_, and 150 μM acetosyringone) into tobacco for 48 h at 22 °C, the fluorescence signal was observed with a living plant imaging system (Berthold, Stuttgart, Germany). The enzyme activities of LUC and REN were determined by using the Dual-Luciferase Reporter Gene Assay Kit (11402ES60, Yeasen, Shanghai, China). In particular, 36 h after injection, the plants used for the cold-response analysis were moved to 8 °C for 3 h and then back to 22 °C for 9 h. The following detection methods were the same as those above.

### 4.7. Yeast One-Hybrid Assay

After the promoters of *BcFLS1*, *BcF3H*, and *BcCHS* were cloned, the predicted combination sequences were replicated three times in series and inserted into the pLacZi vector. At the same time, the ORF of *BcMYB111* was constructed to create the pJG4-5 vector. According to the Yeastmaker™ Yeast Transformation System (TaKaRa, Osaka, Japan), different combinations were co-transferred into yeast cells (EGY48). After culturing in SD/-Trp/-Ura medium at 30 °C, a binding test was performed in Ura-/Trp-deficient SD medium with x-gal.

In the same way, the DRE element of *BcMYB111* was replicated three times in series, inserted into the pABAi vector, and transferred into Y1H Gold yeast. The yeast was screened to determine the lowest concentration of aureobasidin A (AbA) on the SD/-Ura plate. *BcCBF2* and *BcCBF3* were inserted into the pGADT7 vector and transferred into Y1H Gold cells that contained a bait vector. A binding test was performed at 30 °C on SD/-Leu plates containing the corresponding AbA.

### 4.8. Electrophoretic Mobility Shift Assay (EMSA)

The ORF of *BcCBF2* was cloned into the pCold–HIS vector and transformed into *Escherichia coli Rosetta* (DE3). After isopropy-β-D-thiogalactoside (IPTG) was added, the 6× His–BcCBF2 fusion protein was generated. Then, the fusion protein was extracted and enriched using the His-tag Protein Purification Kit (Beyotime, Shanghai, China). The EMSA probe primers were labeled with biotin. Then, the EMSA was performed with the LightShift Chemiluminescent EMSA Kit (Thermo Fisher, MA, USA).

### 4.9. Silencing of BcMYB111 in NHCC by Virus-Induced Gene Silencing (VIGS)

According to the description [73], an 80-bp specific palindromic DNA sequence (5′-ATGAGATCCTCACTAACTATATCCAAACCAATGGGGAAGGCCTTCCCCATTGGTTTGGATATAGTTAGTGAGGATCTCAT-3′) was synthesized by the GeneScript company (Nanjing, China) and recombined into a pTY vector. After being wrapped in gold particles, 4 μg plasmids of PTY and pTY–*BcMYB111* were used to bombard the 2-week-old seedings with a gene gun (Biolistic PDS-1000/He, Bio-rad, Hercules, Waltham, CA, USA). After two weeks of growth, both the pTY and pTY–*BcMYB111* lines were obtained for subsequent research.

### 4.10. Generation of Transgenic Arabidopsis thaliana

The fusion constructs of 35S::BcMYB111–GFP were transformed into GV3101. To generate BcMYB111-overexpressing *Arabidopsis thaliana*, the floral dip method [74] was used. Transgenic plants were selected on 1/2 MS medium containing 25 mg/L of hygromycin and 16 mg/L of timentin. After the homozygous progeny of the transgenic plants were identified via PCR (the primer sequence is shown in Table S2) and Western blotting with the GFP antibody, phenotypic characterizations were performed.

### 4.11. Measurement of MDA, H_2_O_2_, and Enzyme Activity of POD and SOD

First, 0.1 g of fresh plant leaves were cut, ground with liquid nitrogen, and added to 1 mL of the corresponding extraction solution. After an adequate vortex in an ice-water bath, the supernatant was obtained via centrifugation at 8000× *g*. Then, the contents of MDA and H_2_O_2_ and the enzyme activities of POD and SOD were determined (BC0020; BC3595; BC0090; BC0170, Solarbio, Beijing, China).

### 4.12. Agrobacterium Rhizogenes-Mediated Transformation of Hairy Roots

‘Suzhouqing’ seeds were soaked in 75% ethanol for 2 min and 10% sodium hypochlorite for 18 min, washed with ddH_2_O five times, placed in a germination medium (1/2 MS, pH 5.8), and kept in the culture room (23 ± 2 °C, light cycle with 16 h of light and 8 h of dark for 7 days; the light was 2000–3000 lx). *BcMYB111* over-expression vectors were introduced into the *Agrobacterium rhizogenes* strain MSU440, and the bacteria were cultured overnight. After the medium was centrifuged at 5000 rpm for 10 min and the supernatant was discarded, a co-culture liquid medium (MS diluted tenfold (pH5.2) + acetosyringone (100 μM)) was added to make OD600 = 0.8 and placed at 28 °C and 250 rpm for 3 h to form the infection medium. We cut the original main roots of the seedling. Then, the rootless seedlings were soaked in the infection medium for 10 min, washed with ddH_2_O three times, and placed in an MS medium containing timentin (160 mg/L). After 25–35 days, the hairy roots were grown out and used for subsequent experiments after identification.

### 4.13. Statistical Analysis

All of the data are presented as the means and standard deviation (SD) of at least three independent replicates for each experiment. The Student’s two-tailed *t*-test was conducted using GraphPad Prism version 7.0 (GraphPad Software, San Diego, CA, USA).

## 5. Conclusions

**Figure 9:**
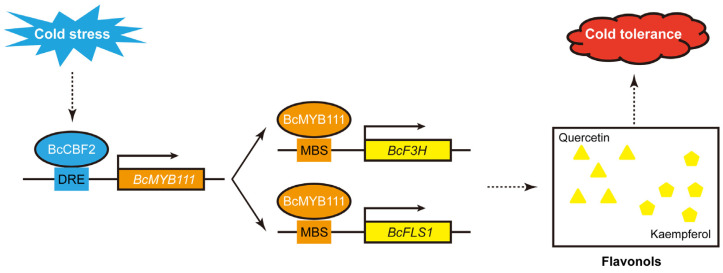
A proposed working model of BcCBF2–BcMYB111–BcF3H/BcFLS1 in flavonol accumulation under cold stress. When NHCC suffers from cold stress, the cold response gene *BcCBF2* is rapidly induced. The BcCBF2 protein directly binds the DRE element of *BcMYB111* to activate its expression. Lots of BcMYB111 proteins further increase the promotion of *BcF3H* and *BcFLS1*. Then, flavonols are largely accumulated, including quercetin and kaempferol, which can improve cold tolerance in NHCC.

In this study, we showed that BcMYB111 acts as a positive regulator of flavonol synthesis by directly binding *BcF3H* and *BcFLS1* in NHCC (Figure 9). *BcMYB111* actively regulates the synthesis of flavonols. *BcMYB111* is directly induced by BcCBF2 to regulate flavonol synthesis under cold stress. Furthermore, BcMYB111 also plays a positive role in promoting tolerance to cold stress. As a result, a model for *BcMYB111*’s regulation of flavonoid metabolism in response to cold stress was proposed.

## Data Availability

Data will be made available on request.

## Supplementary Materials

The following supporting information can be downloaded at: https://www.mdpi.com/article/10.3390/ijms24108670/s1.
