# Supplementary material for: BcMYB111 Responds to BcCBF2 and Induces Flavonol Biosynthesis to Enhance Tolerance under Cold Stress in Non-Heading Chinese Cabbage
Source: Int J Mol Sci. 2023 May 12;24(10):8670. doi: 10.3390/ijms24108670 (PMC10217907; doi:10.3390/ijms24108670)
Supplement: Supplementary file 1 [file ijms-24-08670-s001.zip › Supplementary Figure.pdf]

# *BcMYB111* responds to BcCBF2 and induces flavonol biosynthesis to enhance tolerance under cold stress in non-heading Chinese cabbage

**Supplementary figures:**

**Supplementary Figure S1**

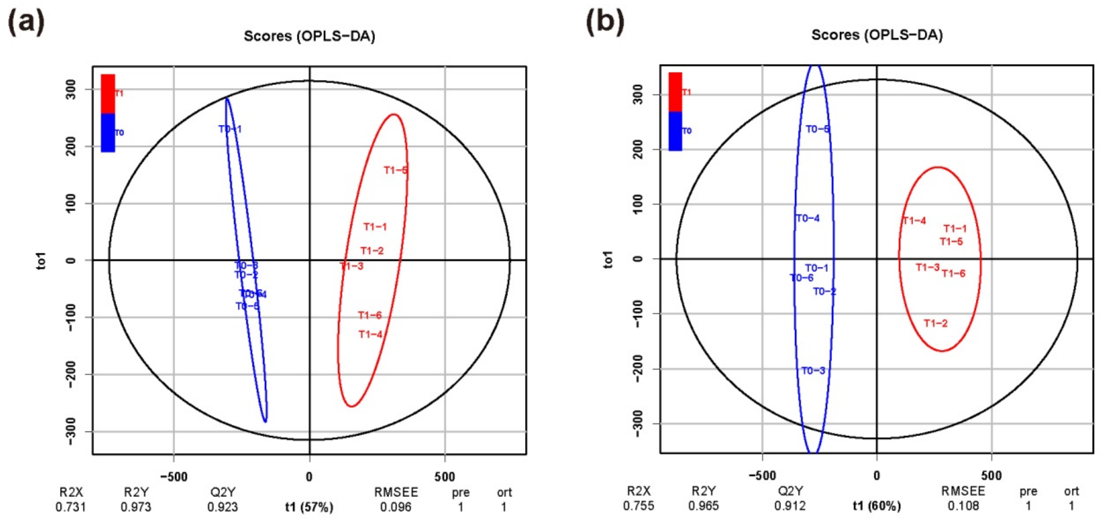


**Figure S1** Orthogonal Partial Least Squares- Discriminant Analysis (OPLS-DA) in the POS (a) and NEG (b) model for the samples of T0 and T1. The model of OPLS-DA was proved to be reliable because the value of Q2 was all over 0.9. Both T0 and T1 group contained six samples.

**Supplementary Figure S2**

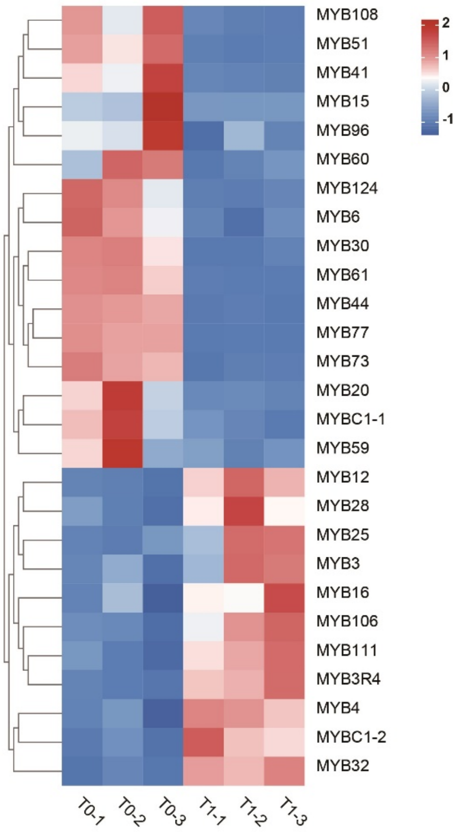


**Figure S2** Cluster and heat map of DEGs of NHCC between T0 and T1. The heat map shows the gene expression obtained by the clustering affinity search technique. Each block refers to the data for one gene. The color bar represents the log2 of fold change values.

**Supplementary Figure S3**

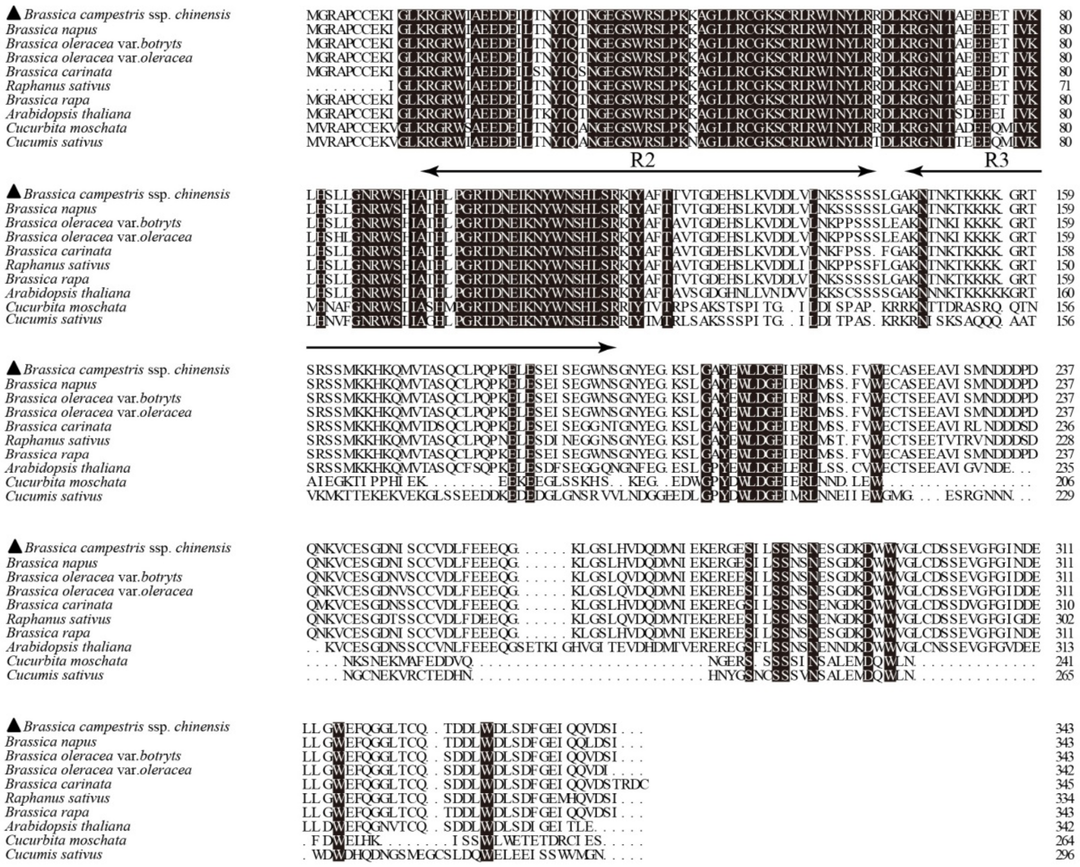

**Figure S3** Multiple alignment of MYB111 proteins. Amino acids identical in all proteins are marked black, and others were marked in white. The sequence of *Brassica campestris* ssp. *chinensis* was highlighted with a black triangle. The other plants were used for the compare containing *Brassica napus* (XP_048591191.1), *Brassica rapa* (XP_009151728.1), *Brassica oleracea* var.*botryts* (QCI34366.1), *Brassica oleracea* var.*oleracea* (XP_013594136.1), *Brassica carinata* (KAG2261091.1), *Raphanus sativus* (XP_004134499.1), *Arabidopsis thaliana* (NP_199744.1), *Cucurbita moschata* (XP_022934658.1), and *Cucumis sativus* (XP_018467577.1). The R2 and R3 MYB domains were marked according to SMART database (http://smart.embl-heidelberg.de/).

**Supplementary Figure S4**

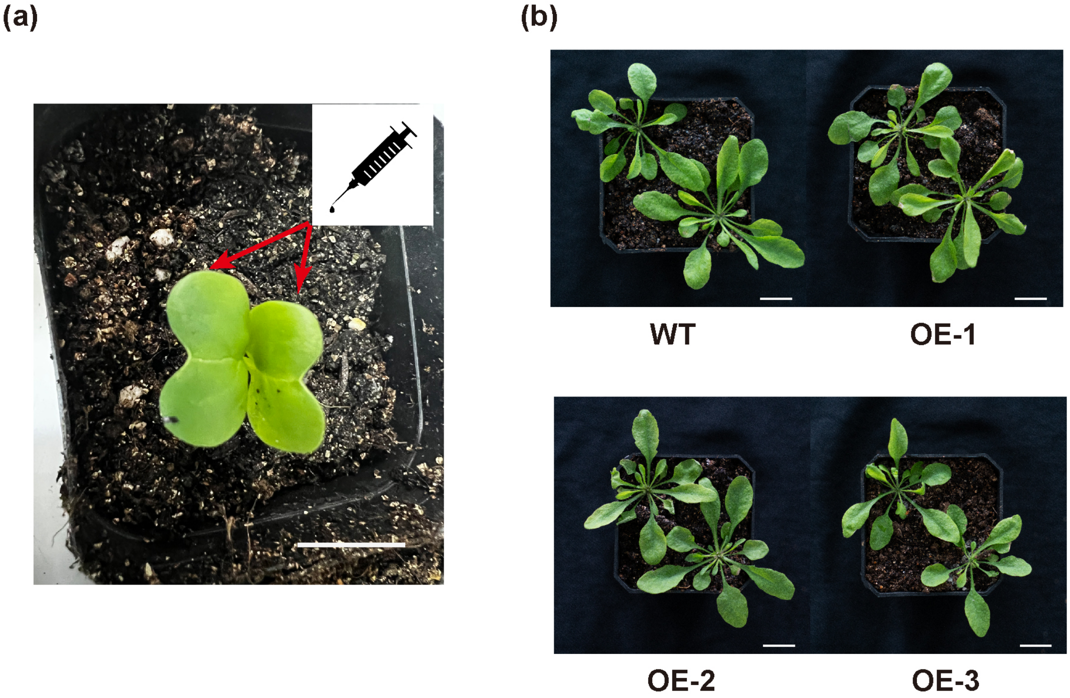


**Figure S4** Phenotypes of the cotyledon of non-heading Chinese cabbage for the transient expression assay (a) and phenotypes of the Arabidopsis (b). (a) The Agrobacterium tumefaciens carried with 35S::*BcMYB111*-GFP and 35S::GFP were separately injected into the left cotyledon and the right cotyledon. Bar: 2cm. (b) The phenotypes of Arabidopsis in wild type (WT) and *BcMYB111* overexpressed lines (OE-1, OE-2 and OE-3). Bar: 2cm.
