# Supplementary material for: BcMYB111 Responds to BcCBF2 and Induces Flavonol Biosynthesis to Enhance Tolerance under Cold Stress in Non-Heading Chinese Cabbage
Source: Int J Mol Sci. 2023 May 12;24(10):8670. doi: 10.3390/ijms24108670 (PMC10217907; doi:10.3390/ijms24108670)
Supplement: Supplementary file 1 [file ijms-24-08670-s001.zip › Supplementary Table S1.pdf]

Supplementary Table S1 -Identification, quantification and annotation for metabolites of T0 and T1

index	T0-1	T0-2	T0-3	T0-4	T0-5	T0-6	T1-1	T1-2	T1-3	T1-4	T1-5	T1-6	control_m	case_mea	log2_FC(	Pvalue	fdr	vip	MS2 nam	MS1 nam	mzCloud	RT [min]	Molecula	Formula	CAS	Δppm	-	C_ID	Pathway	KEGG_B	KEGG_A_class
NEG0000	731.0619	320.9292	635.7339	637.0337	132.1392	304.9866	767.8762	595.7486	595.4935	743.2116	654.9647	802.6902	460.3141	693.3308	0.590925	0.04972	0.068885	0.558132	Glycolic a	Glycolic acid		0.829	76.01421	C2H4O3	79-14-1	24	Hydroxy	C00160	ko01100//	Carbohyc	Metabolism
NEG0000	68391.7	74510	75737.07	59282.47	70497.59	78686.56	72163.04	70469	84852.42	67766.95	80640.33	92618.07	71184.23	78084.97	0.133487	0.183909	0.220344	2.866921	Pyruvate	Pyruvic acid		0.934	88.01426	C3H4O3	127-17-3	19	Keto acid	C00022	ko01100//	Amino ac	Human Diseases;Metabolism
NEG0000	297.5743	13.02077	286.0659	248.7477	8.550722	293.2841	258.5615	276.5405	234.6932	406.4303	535.9065	1169.893	191.2073	480.3375	1.328911	0.094335	0.121821	0.617223	Lactic aci	L-(+)-Lact	99.6	14.365	90.02995	C3H6O3	50-21-5	18	Hydroxy	-	-	-	-
NEG0000	11488.14	4791.525	10416.29	8862.845	10409.18	6507.202	6919.776	5059.186	5410.908	7455.936	10942.16	10059.7	8745.865	7641.277	-0.19479	0.462411	0.496278	0.581337	Sulfuric acid			1.054	97.9666	H2O4S	7664-93-9	7	-	C00059	ko01100//	Amino ac	Environmental Information Processing;Metabolism
NEG0000	665.8937	453.1285	455.5463	333.3244	686.1867	512.4626	406.8253	613.4826	474.3469	246.6227	293.1457	272.7034	517.757	384.5211	-0.42921	0.1279	0.159771	0.366385	Phosphoric acid			0.937	97.97509	H3O4P	7664-38-2	18	Non-meta	C00009	ko01100//	Energy m	Environmental Information Processing;Metabolism
NEG0000	31.64946	15.20255	26.8057	22.674	14.07441	16.68754	21.03821	13.09207	11.13363	13.75373	28.01928	15.32142	21.18228	17.05972	-0.31226	0.312485	0.34914	0.043138	Succinic anhydride			1.237	100.0143	C4H4O3	108-30-5	17	Oxolanes	-	-	-	-
NEG0000	311.2677	276.3947	369.8924	279.9043	171.7781	291.0095	424.11	401.5449	350.5059	416.5136	516.6937	420.4058	283.3745	421.629	0.573264	0.002413	0.005165	0.509895	2-Ketobu	2-Oxobut	90.4	1.264	102.0299	C4H6O3	600-18-0	17	Keto acid	C00109	ko01100//	Amino ac	Metabolism
NEG0000	128.5979	167.8752	149.8552	139.5614	173.8963	175.1639	315.7382	342.946	327.5367	243.8642	391.1843	438.9306	155.825	343.3667	1.139823	6.08E-05	0.000279	0.652528	Malonic a	Malonic acid		0.941	104.0092	C3H4O4	141-82-2	16	Carboxyli	C00383	ko00240//	Metabolis	Metabolism
NEG0000	399.4893	284.4863	354.7302	269.2611	261.5547	338.8398	305.1994	315.3849	279.0414	207.3707	309.2656	405.2851	318.0602	303.5912	-0.06717	0.682869	0.714761	0.018367	Glyceric a	(2R)-2,3-D	73.9	0.873	106.0248	C3H6O4	473-81-4	17	Organoox	C00258	ko01100//	Amino ac	Metabolism
NEG0001	1117.816	391.9286	1315.269	896.974	782.2862	967.8825	744.082	761.8086	587.9008	538.3217	716.7672	844.9921	912.026	698.9788	-0.38383	0.150433	0.183701	0.449094	2-Furoic a	2-Furoic a	69.1	1.237	112.0143	C5H4O3	88-14-2	15	Furans	C01546	ko01120//	Global an	Metabolism
NEG0001	12.82926	12.82037	20.53758	16.29855	5.711546	13.33565	94.67912	31.15946	36.01139	30.70068	59.53061	33.82603	13.58883	47.65121	1.810092	0.009179	0.015969	0.256507	Uracil	Uracil	89.9	1.237	112.0255	C4H4N2(	66-22-8	15	Diazines	C00106	ko01100//	Global an	Metabolism
NEG0001	6.625987	9.997318	11.58793	9.83395	7.20511	14.89009	134.8814	162.2558	141.4874	249.0708	152.8899	217.0853	10.0234	176.2785	4.136413	5.00E-06	4.88E-05	0.575665	L-Proline	Proline		0.837	115.0615	C5H9NO	147-85-3	15	Carboxyli	C00148	ko01100//	Amino ac	Environmental Information Processing;Genetic Information Processing;Metabolism
NEG0001	659.8666	981.2837	883.1646	679.7339	823.73	1227.903	1019.518	564.2551	929.0646	518.2965	882.6649	1031.606	875.947	824.2343	-0.08779	0.691185	0.720379	0.051093	Maleic ac	MALEIC ACID		1.182	116.0092	C4H4O4	110-16-7	15	Carboxyli	C01384	ko01120//	Amino ac	Metabolism
NEG0001	39.77975	21.06836	45.76747	39.7966	23.89408	23.75837	95.22046	79.52882	57.10687	79.06313	83.16241	100.6406	32.3441	82.45372	1.350082	5.94E-05	0.000276	0.331908	Levulinic	Levulinic	86.4	1.797	116.0456	C5H8O3	123-76-2	15	Keto acid	-	-	-	-
NEG0001	164.8674	71.63453	178.936	123.1727	11.10759	86.99647	22.66817	41.57861	39.53432	42.39365	36.43446	58.37693	106.1191	40.16436	-1.4017	0.029546	0.043973	0.311314	Succinic a	Succinic acid		1.336	118.0249	C4H6O4	110-15-6	14	Carboxyli	C00042	ko01100//	Amino ac	Metabolism
NEG0001	22.89941	47.91498	82.59718	60.92622	16.24691	60.45275	33.94383	9.387781	24.35411	38.76748	20.61791	49.37932	48.50624	29.40841	-0.72194	0.136186	0.167919	0.145648	Purine			0.793	120.0416	C5H4N4	120-73-0	5	Imidazop	-	-	-	-
NEG0001	1.558703	0.491823	1.072434	0.277605	0.466535	0.512161	187.8786	176.5194	155.3993	188.1197	423.9409	206.0567	1	222.9858	7.800808	0.00028	0.00084	0.683852	acetopher	Phenylace	99.5	2.697	120.0558	C8H8O	98-86-2	14	Organoox	C07113	ko01120//	Global an	Metabolism
NEG0001	10.12627	7.371586	87.04915	9.244133	11.37105	7.91237	76.4148	73.4388	69.90762	63.51915	75.33224	71.4496	22.17909	71.67704	1.692311	0.003656	0.007179	0.296588	Benzoic a	Benzoic a	96	2.381	122.0351	C7H6O2	65-85-0	13	Benzene a	C00180	ko01100//	Amino ac	Metabolism
NEG0001	69.53637	46.34816	78.65214	62.6149	55.20181	53.35589	38.31866	147.422	95.94832	39.3931	28.25273	206.0003	60.95155	92.55585	0.602661	0.312515	0.34914	0.163265	Pyroglut	4-Oxoprol	96.7	0.917	129.0408	C5H7NO	98-79-3	13	Carboxyli	C01879	ko01100//	Global an	Metabolism
NEG0002	2367.548	1762.449	2556.617	1717.015	2127.238	2515.234	2072.239	2105.67	1868.939	1239.309	2093.606	2131.787	2174.35	1918.591	-0.18054	0.243801	0.28148	0.367708	Itaconic a	Itaconic a	98.8	1.361	130.0248	C5H6O4	97-65-4	13	Fatty Acy	C00490	ko00660//	Carbohyc	Metabolism
NEG0002	46.8331	27.76115	45.30042	39.58238	22.66526	44.95931	264.0316	151.4671	100.1259	207.2181	180.8553	174.7565	37.85027	179.7424	2.247555	9.72E-05	0.000402	0.546945	Ketoleuci	4-Methyl-2-oxovaler		2.347	130.0613	C6H10O3	816-66-0	13	Keto acid	C00233	ko01100//	Amino ac	Metabolism
NEG0002	180.6674	145.8781	219.4663	157.4539	127.1328	202.9807	360.0644	410.0726	279.1623	338.9553	344.7658	482.8635	172.2632	369.314	1.100233	0.000104	0.000426	0.64561	DL-β-Leu	DL-β-Leu	99.4	1.316	131.0929	C6H13N(	5699-54-7	13	Carboxyli	-	-	-	-
NEG0002	173.9067	219.6884	287.6929	209.8768	91.22637	213.5021	663.367	521.815	441.285	607.481	650.304	770.8684	199.3156	609.1867	1.61183	1.86E-05	0.000118	0.954178	Glutaric acid			0.824	132.0405	C5H8O4	110-94-1	13	Carboxyli	C00489	ko01120//	Amino ac	Metabolism
NEG0002	156.2179	142.3566	122.8219	103.506	133.5929	149.1035	197.1616	112.8694	119.2281	191.498	136.5229	270.1127	134.5998	171.2321	0.347277	0.186578	0.221009	0.208533	L-Asparagine			0.785	132.0517	C4H8N2(	70-47-3	13	Carboxyli	C00152	ko01100//	Amino ac	Genetic Information Processing;Metabolism
NEG0002	9.673177	8.947858	8.52223	7.442248	4.065446	9.355493	15.32153	11.67505	13.56403	15.83185	12.8762	18.90302	8.001075	14.69528	0.877087	0.000572	0.00154	0.113999	Ornithine	Ornithine	98.5	0.717	132.0881	C5H12N2	70-26-8	13	Carboxyli	-	-	-	-
NEG0002	954.5516	1604.821	1214.114	864.1201	398.0033	1304.982	3382.842	2484.046	3722.989	3572.297	2939.264	4136.299	1056.765	3372.956	1.674358	1.34E-05	9.66E-05	2.200083	L-Asparti	Aspartic acid		0.788	133.0357	C4H7NO	56-84-8	13	Carboxyli	C00049	ko01100//	Amino ac	Environmental Information Processing;Genetic Information Processing;Metabolism
NEG0002	699.456	643.2549	648.5787	452.7689	403.5205	610.1523	1479.264	545.703	995.1261	2468.674	1039.257	2388.595	576.2886	1486.103	1.366671	0.018919	0.03016	1.138175	4-Hydroxybenzeneacetonitrile			0.788	133.055	C8H7NO	14191-95-8	8	Benzene a	-	-	-	-
NEG0002	176.0316	222.5982	180.8622	120.8941	168.9123	186.221	155.0569	71.23172	209.4551	94.3531	171.7481	217.5941	175.9199	153.2398	-0.19913	0.434	0.467102	0.073731	L-Malic a	(±)-Malic Acid		0.888	134.0209	C4H6O5	97-67-6	4	Hydroxy	C00149	ko01100//	Carbohyc	Metabolism
NEG0002	246.13	196.37	296.7483	258.8234	125.2606	247.6741	458.088	293.439	226.4241	373.5224	368.6426	420.717	228.5011	356.8055	0.642937	0.012635	0.021301	0.473132	Deoxyrib	DEOXYRIBOSE		1.302	134.0561	C5H10O4	533-67-5	13	Organoox	C01801	ko00030//	Carbohyc	Metabolism
NEG0003	16.26257	23.6722	18.10817	11.44447	12.0082	17.93077	116.0996	80.47378	81.66612	152.9653	66.78871	46.48028	16.57106	90.74563	2.453162	0.000789	0.00199	0.347475	Adenine	Adenine	85.3	1.267	135.0526	C5H5N5	73-24-5	14	Imidazop	C00147	ko01100//	Global an	Metabolism
NEG0003	39267.02	40850.25	51566.93	40886.95	29567.21	51263.74	14828.67	21594.07	15375.49	16503.55	21423.75	18230.73	42233.68	17992.71	-1.23098	4.97E-05	0.000239	7.120885	Threonic	Threonic acid		0.811	136.0355	C4H8O5	7306-96-9	12	Organoox	C01620	ko00053//	Carbohyc	Metabolism
NEG0003	1.811592	1.750676	2.354151	1.593935	1.341794	1.920314	142.9353	68.2388	33.40145	145.2019	199.6082	141.8478	1.79541	121.8723	6.084912	0.000638	0.001665	0.498638	2-Methyll	2-Methylb	96.7	2.377	136.0506	C8H8O2	118-90-1	13	Benzene a	C07215	ko01100//	Global an	Metabolism
NEG0003	64.16076	49.79307	66.0583	46.19948	34.59764	42.68647	3.640784	5.527487	2.462061	2.236051	4.895986	5.852467	50.58262	4.102473	-3.62408	3.56E-06	3.99E-05	0.31093	Salicylic a	Salicylic a	99.6	2.042	138.03	C7H6O3	69-72-7	12	Benzene a	C00805	ko01100//	Amino ac	Environmental Information Processing;Metabolism
NEG0003	34.99572	34.52326	48.13887	25.56037	10.84011	21.25792	43.41997	44.35601	39.84501	42.92305	46.92354	46.22355	29.21938	43.94852	0.588889	0.020803	0.032483	0.153716	2-Keto-gl	2-Keto-glutaramic a		0.872	145.0369	C5H7NO	18465-19-5	4	Keto acid	C00940	ko00250//	Amino ac	Metabolism
NEG0003	525.4507	1218.421	784.5302	553.0498	820.2007	1636.987	130.7883	502.6255	121.4918	292.9843	169.4315	128.6748	923.1065	224.3327	-2.04086	0.003713	0.007218	1.136705	Indole-3-	4-Indoleca	99.6	3.274	145.051	C9H7NO	487-89-8	12	Purine nu	-	-	-	-
NEG0003	32803.44	24518.77	38315	6962.057	19228.08	27708.04	20742.44	24985.32	19436.93	16503.18	21793.53	9614.46	24922.57	18845.98	-0.4032	0.250804	0.287821	2.188032	Oxogluta	2-Oxoglut	87.6	1.237	146.0199	C5H6O5	328-50-7	11	Keto acid	C00026	ko01100//	Amino ac	Metabolism
NEG0003	0.287657	0.636741	0.615998	0.212604	0.541937	0.721561	6.904905	4.216028	3.809126	5.463184	3.285317	3.845713	1	4.587379	2.19767	2.54E-05	0.000146	0.091722	Adipic ac	(3R)-4,4-D	66.5	1.701	146.0573	C6H10O4	124-04-9	4	Fatty Acy	C06104	ko01100//	Global an	Metabolism
NEG0003	2207.485	1558.648	1390.804	1081.27	1126.596	1527.838	2245.145	1230.253	2631.855	3852.257	1760.217	4180.373	1482.107	2650.017	0.838352	0.042617	0.059915	1.199385	L-Glutam	Glutamine		0.781	146.0674	C5H10N2	56-85-9	11	Carboxyli	C00064	ko01100//	Amino ac	Environmental Information Processing;Genetic Information Processing;Metabolism
NEG0003	74.75392	61.85083	68.55938	50.80966	48.59293	64.88494	168.0037	132.457	129.3668	106.1963	153.5208	145.9769	61.57528	139.2536	1.177291	1.18E-05	8.99E-05	0.426339	L-Lysine	L-(+)-Lysine		0.697	146.1039	C6H14N2	56-87-1	11	Carboxyli	C00047	ko01100//	Amino ac	Environmental Information Processing;Genetic Information Processing;Metabolism
NEG0004	60.29708	91.20149	118.5737	76.05409	54.91578	128.6225	219.1468	88.85971	74.05999	136.7761	159.3369	373.275	88.27744	175.2424	0.989235	0.091642	0.119574	0.364853	L-Glutam	L-Glutamic acid		0.79	147.0514	C5H9NO	56-86-0	11	Carboxyli	C00025	ko01100//	Amino ac	Environmental Information Processing;Genetic Information Processing;Metabolism
NEG0004	420.0248	229.8404	387.7578	322.2394	88.84615	417.0032	345.4331	275.4754	263.909	349.5199	324.4904	340.7028	310.952	316.5884	0.025917	0.920986	0.928296	0.002188	Citramali	(-)-Citramalic acid		1.399	148.0354	C5H8O5	2306-22-1	11	Fatty Acy	-	-	-	-
NEG0004	49.43977	61.89092	78.2842	60.24882	47.34496	59.45567	71.52656	75.57487	56.33153	76.87494	76.03993	97.26171	59.44406	75.60159	0.346884	0.043463	0.06084	0.155348	L-2-Hydr	(+/-)-2-Hydroxyglut		0.809	148.0364	C5H8O5	13095-48-2	5	Fatty Acy	C03196	ko01120//	Global an	Metabolism
NEG0004	0.774979	0.589549	0.564093	0.464922	0.694996	0.526729	24.17532	13.42635	5.556592	21.02197	40.03436	16.68266	1	20.14954	4.332675	0.002152	0.004704	0.197177	Cinnamic	trans-Cin	66	4.072	148.0507	C9H8O2	621-82-9	11	Cinnamic	-	-	-	-
NEG0004	412.1797	327.2868	476.6176	359.3174	249.0899	379.7945	391.6489	249.2473	202.08	429.6285	327.9766	396.6428	367.381	332.8707	-0.14232	0.493598	0.523846	0.127438	Mevalonic acid			1.641	148.0718	C6H12O4	150-97-0	11	Fatty Acy	C00418	ko01100//	Global an	Metabolism
NEG0004	36.3883	35.80286	46.65099	28.82216	24.80775	37.83903	69.19244	76.56467	78.87022	81.47962	95.0836	99.67879	35.05185	83.47822	1.251909	6.55E-06	5.55E-05	0.323569	L-Methionine			1.235	149.0493	C5H11N(	63-68-3	11	Carboxyli	C00073	ko01100//	Amino ac	Genetic Information Processing;Metabolism
NEG0004	32.45361	45.00449	59.93561	48.35231	20.68689	38.96153	154.2546	84.91203	85.71663	114.1537	127.6671	186.2562	40.89907	125.4934	1.617471	0.000588	0.001566	0.427049	L-Xylulose			0.814	150.052	C5H10O5	527-50-4	5	Organoox	C00312	ko01100//	Carbohyc	Metabolism
NEG0004	0.906639	0.245008	1.49186	1.083689	0.881562	0.722927	70.246	89.15312	65.65533	69.84103	89.39328	90.19116	1	79.07999	6.305241	1.42E-08	2.70E-06	0.422132	hydrocin	[Similar to: Mono(2-		2.802	150.0663	C9H10O2	501-52-0	11	Phenylpr	C05629	ko01120//	Amino ac	Metabolism
NEG0004	16.01743	10.83192	20.02787	13.89757	19.71389	13.12299	22.50216	31.21794	21.25229	8.21594	20.74845	11.35663	15.60195	19.21557	0.30055	0.352828	0.387399	0.055022	Vanillin	Vanillin	80.5	3.497	152.0457	C8H8O3	121-33-5	10	Phenols	C00755	ko01100//	Amino ac	Metabolism
NEG0004	5.723871	6.127472	6.255956	4.439421	12.8278	4.72743	15.68106	9.536349	6.549819	7.826	11.80556	8.022933	6.683658	9.90362	0.567318	0.114825	0.145343	0.070085	p-Anisic a	2-Anisic a	83.5	2.154	152.0457	C8H8O3	100-09-4	10	Benzene a	C02519	ko01220//	Global an	Metabolism
NEG0005	2.827326	1.803249	3.212124	2.243153	1.541301	6.421254	17.24343	13.97094	11.99291	16.85933	18.37462	22.66909	3.008068	16.85172	2.485987	8.83E-06	7.15E-05	0.174271	3-Hydrox	3-Aminos	91.2	4.527	153.0409	C7H7NO	548-93-6	11	Benzene a	C00632	ko01100//	Amino ac	Metabolism
NEG0005	30.26444	19.50686	22.23226	17.36267	20.32947	20.93299	72.20049	46.99705	43.92006	59.07901	57.23664	60.44635	21.77145	56.6466	1.379552	1.67E-05	0.000111	0.278646	L-Histidi	L-Histidir	96.4	0.73	155.0677	C6H9N3(	71-00-1	10	Carboxyli	C00135	ko01100//	Amino ac	Environmental Information Processing;Genetic Information Processing;Metabolism
NEG0005	799.7432	788.8731	1000.739	749.5933	706.1362	917.0826	961.0826	848.1742	857.4638	773.204	921.9709	1055.667	827.0279	902.927	0.126673	0.238687	0.277255	0.300048	Phosphoglycolic acid			14.579	155.9798	C2H5O6F	13147-57-4	16	Organic p	C00988	ko01100//	Carbohyc	Metabolism
NEG0005	6.583412	5.435676	5.810995	6.57765	2.853145	4.268124	6.625116	6.713129	8.456256	5.064142	8.52699	8.139707	5.254834	7.254224	0.465176	0.034089	0.049012	0.056764	Oxoadipi	2-OXOADIPATE		1.332	160.0355	C6H8O5	3184-35-8	10	Keto acid	C00322	ko01100//	Amino ac	Metabolism
NEG0005	25.70241	30.78169	27.5577	25.92943	37.85424	42.26454	25.2381	24.21568	18.97051	21.63529	20.89612	22.45158	31.68167	22.23455	-0.51085	0.009599	0.016623	0.123323	Pimelic a	3-Methyla	95.1	2.316	160.072	C7H12O4	111-16-0	9	Fatty Acy	C02656	ko01100//	Global an	Metabolism
NEG0005	15.75249	4.477593	7.375421	1.927934	102.3978	2.380889	451.9205	378.796	315.5794	149.3252	224.247	196.3894	22.38536	286.0429	3.675604	0.000368	0.001054	0.739992	Indole-3-	1,5-Isoqui	78.6	3.491	161.0461	C9H7NO	771-50-6	9	Indoles a	-	-	-	-
NEG0005	7.986949	7.664607	10.26254	6.699169	4.801306	6.1845	17.86732	16.69855	13.04993	13.31668	16.42341	12.8274	7.266512	15.03055	1.048563	6.20E-05	0.000281	0.129777	L-2-Aminoadipic acid			1.243	161.0671	C6H11N(	1118-90-7	10	Carboxyli	C00956	ko01100//	Amino ac	Metabolism
NEG0005	323.9095	386.6649	454.5135	428.6818	220.1878	368.4935	1472.832	1029.793	790.1591	1099.981	1259.764	1571.124	363.7418	1203.942	1.72678	4.73E-05	0.000231	1.379771	Levogluc	[Similar to: (3β,5ξ,9ξ		0.806	162.0511	C6H10O5	498-07-7	10	Oxepanes	-	-	-	-
NEG0005	0.981708	0.941908	0.524479	0.407307	5.972659	2.065113	9.651411	2.557089	0.451716	1.43956	6.730972	0.8875	1.815529	3.619708	0.995483	0.326905	0.362066	0.045804	Raphanus	XN0294000		1.932	162.9755	C4H5NO	98169-56-3	4	Carboxyli	-	-	-	-
NEG0005	9.138588	4.396446	6.204774	5.090289	8.839669	4.47624	62.27465	78.22894	25.33963	41.48903	98.04638	78.61335	6.357668	63.99866	3.331472	0.000368	0.001054	0.357305	Phenylpy	Phenylpy	84	1.556	164.0456	C9H8O3	156-06-9	10	Benzene a	C00166	ko01100//	Amino ac	Metabolism
NEG0006	1777.612	1404.841	1710.138	1306.753	1039.603	1650.399	3293.6	3310.224	2671.821	3004.532	3045.081	3521.51	1481.558	3141.128	1.084168	1.76E-06	2.92E-05	1.921566	L-Phenyla	L-Phenyla	86.7	1.693	165.0773	C9H11N(	63-91-2	10	Carboxyli	C00079	ko01100//	Amino ac	Environmental Information Processing;Genetic Information Processing;Metabolism
NEG0006	22.08621	8.89273	26.03564	22.1585	9.762808	10.04192	7.604121	8.318438	7.214667	5.489207	6.394339	6.71563	16.4963	6.956067	-1.2458	0.013411	0.022313	0.122115	Phthalic a	[Similar to: (1'S,2'S)-		1.603	166.0249	C8H6O4	88-99-3	10	Benzene a	C01606	ko01100//	Global an	Environmental Information Processing;Metabolism
NEG0006	39.75849	22.84124	868.222	30.27505	66.40018	30.56521	66.32648	80.91038	61.06618	71.0019	73.09874	87.1172	176.3437	73.25348	-1.26742	0.474017	0.507305	0.230679	Isophthal	Isophthali	97.4	2.371	166.0249	C8H6O4	NA	9	-	-	-	-	-
NEG0006	2369.706	2496.45	2957.644	2458.712	1607.748	3216.408	120.3462	279.9924	171.4529	104.6405	244.0623	110.6738	2517.778	171.8614	-3.87283	1.24E-06	2.25E-05	2.274065	??D-??Xyl	D-Xylonic acid		0.814	166.047	C5H10O6	526-91-0	4	-	-	-	-	-
NEG0006	6.784043	18.60172	6.530537	5.50937	68.81724	19.37191	56.2161	42.86021	40.88759	16.88193	36.2862	20.27499	20.9358	35.56784	0.764601	0.235848	0.274796	0.124828	Homoger	Homogen	93.9	1.944	168.0407	C8H8O4	451-13-8	9	Phenols	C00544	ko01100//	Amino ac	Metabolism
NEG0006	0.484251	0.676327	0.956336	0.171714	0.594727	1.37781	11.73884	6.83728	5.856227	5.631955	9.254627	9.5798	1	8.149787	3.026762	2.32E-05	0.00014	0.131864	Vanillic a	Vanillic a	92.6	2.848	168.0407	C8H8O4	121-34-6	9	Benzene a	C06672	ko01120//	Global an	Metabolism
NEG0006	0.317514	0.356504	0.235701	0.353387	0.578229	0.660046	4.983685	2.700726	1.665124	3.020184	6.378724	2.840452	1	3.598149	1.847255	0.001209	0.002861	0.081556	Gallic aci	Gallic acid		1.979	170.02	C7H6O5	149-91-7	9	Benzene a	C01424	ko01120//	Global an	Metabolism
NEG0006	5248.589	3771.667	5116.638	3632.328	5064.051	5130.328	3559.969	4540.018	4111.954	2549.668	4310.978	4384.215	4660.6	3909.467	-0.25354	0.112215	0.142514	0.808116	Aconitic a	trans-Aco	69.4	1.357	174.0148	C6H6O6	4023-65-8	9	Carboxyli	C02341	ko00660//	Carbohyc	Metabolism
NEG0006	397.9823	318.6045	214.2308	263.8764	314.8641	309.7438	233.5608	287.5103	225.7817	134.3296	257.3134	171.8838	303.217	218.3966	-0.4734	0.03171	0.046289	0.316065	Dehydroa	Dehydroascorbic aci		1.235	174.0148	C6H6O6	490-83-5	9	Lactones	C05422	ko01100//	Carbohyc	Metabolism
NEG0006	54.26401	56.23786	67.54072	56.11332	59.86525	71.99294	56.28409	75.37411	56.93532	115.3996	66.065	96.1212	61.00235	77.69655	0.348986	0.128676	0.160215	0.108837	Suberic a	Suberic ac	94.6	2.67	174.0876	C8H14O4	505-48-6	9	Fatty Acy	-	-	-	-
NEG0007	85.11973	61.76828	86.86775	68.94696	44.25643	77.40057	125.3503	104.7081	101.412	111.2155	101.6998	126.4903	70.72662	111.8127	0.660758	0.000472	0.001314	0.284291	L-Arginir	L(+)-Arginine		0.721	174.1101	C6H14N4	74-79-3	9	Carboxyli	C00062	ko01100//	Amino ac	Environmental Information Processing;Genetic Information Processing;Metabolism
NEG0007	42.68527	44.88135	51.93862	36.68246	67.31517	52.64983	4.613114	12.544	5.371566	4.224116	3.704133	3.442698	49.35878	5.649938	-3.127	2.35E-06	3.14E-05	0.305805	3-Indolea	Guanidin	71	4.459	175.0617	C10H9N(	87-51-4	8	Indoles a	C00954	ko01100//	Amino ac	Environmental Information Processing;Metabolism
NEG0007	5.003747	3.646982	6.032782	3.837371	5.925902	4.217378	4.402834	4.117762	4.230565	9.078211	5.517423	13.18429	4.77736	6.755181	0.499781	0.232205	0.27138	0.042027	L-Ascorbi	Ascorbic acid		1.239	176.0304	C6H8O6	50-81-7	9	Dihydrof	C00072	ko01100//	Carbohyc	Metabolism
NEG0007	118.011	97.93157	109.5022	86.33101	15.40853	108.2317	239.8714	358.5575	322.5243	283.1124	367.3701	534.662	89.23599	351.0163	1.97584	0.00015	0.000535	0.739964	2-Isoprop	2-Isopropylmalic aci		2.211	176.067	C7H12O5	3237-44-3	8	Fatty Acy	C02504	ko01100//	Amino ac	Metabolism
NEG0007	5.589061	3.42156	3.535273	2.801738	1.030903	7.766348	8.434693	4.492745	2.790674	4.75108	7.019552	7.954697	4.024147	5.90724	0.553801	0.184567	0.220439	0.047762	Alliin			1.657	177.0444	C6H11N(	556-27-4	8	Carboxyli	-	-	-	-
NEG0007	0.129179	0.325034	0.199196	0.341754	0.175022	0.325588	358.6226	170.6973	93.29407	329.6269	541.6301	359.8496	1	308.9534	8.271246	0.000748	0.001901	0.804521	esculetin	esculetin		2.382	178.025	C9H6O4	305-01-1	9	Coumarir	-	-	-	-
NEG0007	1214.951	982.9215	1790.871	1578.374	668.0532	1017.827	537.8121	517.9948	429.6038	717.3356	770.5512	845.715	1208.833	636.5021	-0.92538	0.010328	0.017725	0.956159	Gulonola	D-GULONIC ACID		0.856	178.0462	C6H10O6	1128-23-0	8	Lactones	-	-	-	-
NEG0007	1.282565	0.934488	0.781866	0.722655	0.37024	1.099957	1129.105	596.4554	326.5256	1027.549	1829.846	1062.072	1	995.2588	9.958928	0.000805	0.002007	1.440233	Caffeic A	Caffeic ac	99	2.379	180.0406	C9H8O4	331-39-5	9	Cinnamic	-	-	-	-
NEG0007	2.890003	1.987749	3.162392	2.606987	3.221004	3.624178	1.363925	2.15569	0.982493	1.784739	2.421432	1.630338	2.915386	1.723103	-0.75868	0.003576	0.007071	0.046242	5-Methylt	5-methylthioribose		1.702	180.0449	C6H12O4	624740-12-	4	Organoox	C03089	ko01100//	Amino ac	Metabolism
NEG0007	201.2821	215.2954	241.1847	173.7687	157.0278	206.6618	494.7148	329.0555	262.7355	335.8822	423.5481	487.9167	199.2034	388.9755	0.965437	0.000848	0.002085	0.649362	α-D-Gluc	Glucose		1.29	180.0617	C6H12O6	492-62-6	8	Organoox	C00031	ko01100//	Biosynthe	Environmental Information Processing;Human Diseases;Metabolism
NEG0008	154.2309	125.6402	133.8513	82.87516	91.4508	135.7172	360.034	295.0589	246.4233	298.6224	270.2681	353.4102	120.6276	303.9695	1.333366	6.94E-06	5.75E-05	0.640336	L-Tyrosin	L-Tyrosin	91.5	1.264	181.0723	C9H11N(	60-18-4	8	Carboxyli	C00082	ko01100//	Amino ac	Genetic Information Processing;Metabolism
NEG0008	31.48945	25.48497	27.48825	25.47211	42.59316	44.65185	20.63659	31.91828	26.0983	16.48924	23.80197	42.0747	32.8633	26.83651	-0.29228	0.266507	0.303102	0.06295	Homovar	Homovanillic acid		2.564	182.0564	C9H10O4	306-08-1	8	Phenols	C05582	ko01100//	Amino ac	Metabolism
NEG0008	419.506	605.0777	311.9612	264.3641	686.7174	631.6878	410.8349	421.4878	595.2416	202.1676	419.9	275.4725	486.5524	387.5174	-0.32833	0.305561	0.344434	0.248383	D-Glycerate 3-phosphate			0.92	185.9913	C3H7O7F	3443-57-0	8	Carbohyc	C00197	ko01100//	Amino ac	Metabolism
NEG0008	239.9507	259.9468	320.9017	256.5375	215.5339	266.3032	262.4839	248.5597	279.2229	303.1555	282.3012	418.6026	259.8623	299.0543	0.20266	0.204499	0.240476	0.197793	Nonaned	Azelaic ac	97.8	3.106	188.1033	C9H16O4	123-99-9	8	Fatty Acy	-	-	-	-
NEG0008	132.1657	114.0493	158.5962	122.0734	117.3239	128.9975	79.91562	71.52823	75.23508	74.06486	81.21569	67.61163	128.8677	74.92852	-0.7823	1.42E-05	0.0001	0.336655	Glycyl-L-	Glycyl-L-l	66.1	1.766	188.1146	C8H16N2	869-19-2	7	Carboxyli	-	-	-	-

NEG0008	1.536795	1.205454	1.723155	1.576435	2.422307	1.31636	53.00901	24.79553	9.541541	29.43388	62.64735	24.2992	1.630084	33.95442	4.380581	0.002609	0.005523	0.256767	9-Hydrox	10-Hydro:	96.4	3.76	188.1398	C10H20O	1422-27-1	7	Hydroxy	-	-	-	-
NEG0008	13.44781	16.38043	19.46403	13.06917	10.93562	14.53892	29.68917	29.06609	17.63631	27.82691	34.64633	26.87382	14.63933	27.6231	0.916026	0.000515	0.001421	0.164207	N-Acetyl	N-Acetylglutamic ac		0.894	189.0621	C7H11NO	1188-37-0	8	Carboxyli	C00624	ko01100//	Amino ac	Metabolism
NEG0008	4.843295	5.553598	2.642294	0.973872	4.447979	4.178105	7.099705	5.347818	1.634634	5.929335	4.011429	7.733062	3.773191	5.292664	0.488209	0.21059	0.246876	0.04364	3-Dehydroquinic acid			1.397	190.0462	C7H10O6	10534-44-8	8	Alcohols	C00944	ko01100//	Amino ac	Metabolism
NEG0008	0.245353	0.10598	0.366442	0.27246	0.107852	0.129484	0.291952	0.421656	0.268925	0.279488	0.288674	0.391162	1	1	0	0.042271	0.05965	0.012746	5-Hydrox	5-Hydrox	81.9	3.08	191.0568	C10H9NO	54-16-0	7	Indoles ar	C05635	ko01100//	Amino ac	Metabolism
NEG0008	45963.53	48349.55	56653.45	42625.27	30852.36	44023.72	42426.85	52257.43	44051.17	36063.27	46862.72	50063.54	44744.65	45287.5	0.017398	0.899132	0.915607	0.518422	Citric acid	Citric acid	98.8	0.871	192.0255	C6H8O7	77-92-9	7	Carboxyli	C00158	ko01100//	Amino ac	Metabolism
NEG0009	958.5276	552.8889	485.9242	715.9331	540.6347	682.3872	317.0284	167.7812	140.2656	173.2502	253.5768	311.9301	656.0493	227.3054	-1.52917	0.000242	0.000754	0.889719	Quinic ac	D-(-)-quinic acid		0.835	192.0617	C7H12O6	77-95-2	8	Quinoline	C00296	ko00400//	Amino ac	Metabolism
NEG0009	1304.846	1295.427	1909.91	1228.757	659.1651	1717.718	814.6579	973.1895	660.9028	687.8361	1028.668	668.0895	1352.637	805.5573	-0.74772	0.01619	0.026138	0.920818	D-Glucuronic acid			0.824	194.0417	C6H10O7	1700908	4	Furofurar	-	-	-	-
NEG0009	1.176222	1.009466	1.132725	3.275183	0.959191	1.23975	161.22	118.1052	77.63165	452.5135	152.0682	611.7637	1.465423	262.217	7.483301	0.014721	0.023969	0.63117	ferulic aci	Ferulic aci	95.4	2.972	194.0562	C10H10O	1135-24-6	8	Cinnamic	C01494	ko01100//	Biosynthe	Metabolism
NEG0009	40743.61	42337.47	53721.45	42784.71	30852.8	53467.77	14099.65	21215.52	14953.21	15845.86	20739.06	17482.55	43984.63	17389.31	-1.3388	3.16E-05	0.000165	7.497596	Gluconic	Gluconic a	60.8	0.809	196.0566	C6H12O7	526-95-4	8	Organoox	C00257	ko01100//	Carbohyd	Metabolism
NEG0009	4.410139	5.175476	6.444118	4.972138	4.685337	9.372306	18.22398	23.95328	11.68062	24.57635	29.04741	32.16936	5.843252	23.27517	1.993948	0.000232	0.000729	0.189116	Syringic acid			1.858	198.0548	C9H10O5	530-57-4	10	Benzene a	C10833	ko01120//	Global an	Metabolism
NEG0009	14.45956	47.68291	9.135478	6.290623	9.746457	47.61438	2.097545	5.40928	4.139119	5.429052	3.28184	1.834402	22.48823	3.69854	-2.60414	0.041833	0.059251	0.16072	Isowillardiine			3.472	199.0618	C7H9N3O	21381-33-9	12	Carboxyli	-	-	-	-
NEG0009	73.01616	65.37715	81.35368	64.29631	64.33451	76.11298	64.34289	64.75591	62.76379	65.85291	65.441	79.48172	70.74847	67.10637	-0.07625	0.367914	0.402802	0.044956	Camalexi	camalexin		1.952	200.0418	C11H8N2	135531-86-	4	Indoles ar	-	-	-	-
NEG0009	29.16884	23.44037	49.97369	33.9341	14.81293	28.31925	19.1641	15.58686	9.124009	17.34475	20.4939	30.02884	29.94153	18.62374	-0.68501	0.06905	0.091666	0.111727	(±)-Camphoric acid			3.242	200.1034	C10H16O	5394-83-2	7	Carboxyli	-	-	-	-
NEG0009	9.957632	8.275014	9.6352	8.819863	7.813755	9.881542	14.86913	13.90025	11.20423	10.44355	15.83989	14.79942	9.063834	13.50941	0.575771	0.000959	0.002313	0.098955	Lauric aci	Lauric aci	98.6	8.654	200.176	C12H24O	143-07-7	8	Fatty Acy	C02679	ko01100//	Global an	Metabolism
NEG0009	153.5511	138.1732	195.6922	55.46838	143.8785	184.5687	41.44873	39.49887	32.44331	31.13449	43.91022	39.98967	145.222	38.07088	-1.9315	0.000363	0.001054	0.45022	Alanyl-Le	Ala-Leu		1.765	202.1303	C9H18N2	3303-34-2	7	Carboxyli	-	-	-	-
NEG0010	453.7212	267.3269	425.9433	270.1805	263.0399	323.8879	885.5571	768.9766	676.2199	764.0497	759.6975	847.5521	334.0166	783.6755	1.230336	1.98E-06	3.01E-05	1.002725	L-Tryptop	D-(+)-Tryp	84	1.942	204.0884	C11H12N	73-22-3	7	Indoles ar	C00078	ko01100//	Amino ac	Genetic Information Processing;Metabolism
NEG0010	1.777514	1.725688	2.380483	2.374495	1.367054	1.372685	0.366814	0.744963	0.515962	0.552986	0.364932	2.146455	1.832986	1	-0.8742	0.010581	0.018077	0.039132	2-Naphth	2-Naphtha	97.3	2.865	208.0179	C10H8O3	120-18-3	7	-	-	-	-	-
NEG0010	73.46336	72.61258	109.2693	95.74276	109.8526	103.1551	217.7889	167.6506	107.3006	156.2531	159.5321	53.74856	94.01595	143.7123	0.612206	0.065587	0.08768	0.243701	Garcinia acid			0.953	208.0203	C6H8O8	27750-10-3	7	Carboxyli	-	-	-	-
NEG0010	0.17577	0.127069	0.290622	0.138989	0.130221	0.157797	3.586693	1.064791	0.553511	3.978721	14.97607	4.893644	1	4.842239	2.275674	0.054175	0.073981	0.085311	Fraxetin	3-[(1-Carboxyvinyl)(		2.396	208.0358	C10H8O5	574-84-5	6	-	-	-	-	-
NEG0010	125.0738	102.6446	140.5099	135.8991	209.4242	130.0498	545.2549	397.144	223.8758	408.6441	468.2103	522.2491	140.6002	427.563	1.604538	0.000176	0.000588	0.798042	Lipoic acid, reduced			0.841	208.0568	C8H16O2	462-20-4	11	Fatty Acy	-	-	-	-
NEG0010	15.7954	10.20166	18.35288	14.28968	7.602293	13.02977	10.35627	10.91246	6.984143	11.28886	10.34195	12.21124	13.21195	10.34915	-0.35233	0.13092	0.162477	0.055065	Chalcone			2.654	208.0915	C15H12O	94-41-7	12	Linear 1,3	-	-	-	-
NEG0010	46666.3	49920.49	58852.24	44105.43	30960.69	45990.32	47561.81	57079.3	47855.92	40343.3	51975.49	55054.79	46082.58	49978.43	0.117084	0.401859	0.437452	1.832749	Galactari	MUCIC ACID		0.826	210.036	C6H10O8	526-99-8	7	Organoox	C00879	ko00053//	Carbohyd	Metabolism
NEG0010	29.15337	28.36273	32.16419	25.05362	14.56089	28.68512	38.04816	52.04284	61.96176	39.57504	114.472	54.37834	26.32998	60.07969	1.190171	0.01675	0.026928	0.237988	5-Hydrox	2,4-diacetylphlorogl		2.392	210.0515	C10H10O	110642-42-	6	Cinnamic	C05619	ko01100//	Biosynthe	Metabolism
NEG0010	821.5871	1128.534	1952.083	1203.573	643.289	1263.841	931.5286	883.1623	705.3202	781.0881	856.7895	996.9769	1168.818	859.1442	-0.44408	0.133173	0.164736	0.558052	Sedoheptulose			0.858	210.0724	C7H14O7	3019-74-7	7	Organoox	C02076	ko00710//	Energy m	Metabolism
NEG0010	2.828544	3.519665	4.325527	3.27494	0.666703	4.314659	67.11684	48.65659	48.55183	78.24181	112.1358	85.45194	3.155006	73.35913	4.539262	3.36E-05	0.000173	0.390385	Eudesmic acid			2.03	212.0671	C10H12O	118-41-2	6	Benzene a	-	-	-	-
NEG0011	5.931658	4.728392	3.298956	4.428768	7.221914	2.710547	23.99797	16.39138	20.45308	7.590328	21.48211	13.51735	4.720039	17.23871	1.868781	0.00062	0.001629	0.165212	cucurbic a	[Similar to: Corchori		4.111	212.1396	C12H20O	58240-50-9	7	Fatty Acy	-	-	-	-
NEG0011	117.3318	133.6051	132.5851	85.02611	74.10092	144.2221	39.10611	58.89878	26.82648	50.65353	49.01605	64.83859	114.4785	48.22326	-1.24728	0.00045	0.001261	0.353992	Dihydrojasmonic acid			5.25	212.1399	C12H20O	98674-52-3	6	Lineolic a	-	-	-	-
NEG0011	837.8523	611.1744	880.7807	855.8348	469.0374	586.4732	677.5222	405.2415	404.0021	265.7939	579.8517	325.0614	706.8588	442.9121	-0.6744	0.019685	0.031249	0.576677	1-Deoxy-D-xylulose 5-phospha			0.854	214.023	C5H11O7	190079-18-	5	Organoox	C11437	ko01100//	Global an	Metabolism
NEG0011	1.744178	1.342576	2.527586	1.969014	2.60806	1.704018	2.15757	2.183338	1.762514	1.715535	1.930895	2.174926	1.982572	1.987463	0.003555	0.982763	0.982763	0.001958	Succinylproline			1.879	215.078	C9H13NO	63250-32-8	6	-	-	-	-	-
NEG0011	16.80957	34.07312	27.86391	25.84519	32.14142	30.88398	15.43873	22.49593	44.97371	129.8929	31.97558	35.66263	27.9362	46.73991	0.742519	0.303575	0.343211	0.070839	N-Nonanoylglycine			3.276	215.1508	C11H21N	NA	6	Carboxyli	-	-	-	-
NEG0011	2.444704	0.145574	0.885798	0.207413	1.315732	0.959789	0.203167	0.189796	7.718611	0.162657	0.191826	0.237283	1	1.450557	0.536607	0.732283	0.760218	0.005286	L-1,2,3,4-Tetrahydro-beta-carb			4.605	216.0884	C12H12N	42438-90-4	6	Indoles ar	-	-	-	-
NEG0011	2.013202	1.386173	1.78997	1.432845	1.617547	1.401114	84.55327	44.10934	15.24278	50.81791	123.5685	45.42477	1.606809	60.61943	5.23751	0.003425	0.006832	0.344457	12-Hydro	12-Hydro:	89	4.982	216.1712	C12H24O	505-95-3	6	Hydroxy	-	-	-	-
NEG0011	192.9153	130.328	237.8794	187.1676	145.2879	206.5455	154.9371	125.7719	100.9115	125.9198	142.2667	128.9273	183.354	129.7891	-0.49846	0.013228	0.022104	0.288306	Pantother	Pantothenic acid		1.768	219.1094	C9H17NO	137-08-6	5	Alcohols	C00864	ko01100//	Global an	Metabolism
NEG0011	116.087	71.08983	90.59872	90.31462	42.08231	107.4852	32.61951	45.74294	54.6262	57.48702	28.87466	19.42606	86.27627	39.79607	-1.11634	0.004048	0.00775	0.308941	Isofraxidin			2.456	222.0549	C11H10O	486-21-5	9	-	-	-	-	-
NEG0011	17.77018	12.59623	19.80467	14.94689	9.250489	14.29866	11.06094	10.00176	11.19603	13.00035	11.83791	19.32368	14.77785	12.73678	-0.21444	0.344078	0.378884	0.034881	Flavone			3.135	222.0725	C15H10O	525-82-6	19	Flavonoic	-	-	-	-
NEG0012	789.7739	662.4962	716.051	661.6149	523.4191	629.2933	899.5483	751.349	706.2033	665.3412	1008.061	783.2647	663.7747	802.2945	0.273438	0.055174	0.074792	0.489818	Hydroxyanthraquinone			0.855	224.0517	C14H8O3	129-43-1	19	-	-	-	-	-
NEG0012	7.15994	6.400213	6.356842	4.229824	6.664648	5.122194	5410.842	3470.005	1613.722	2736.871	4760.362	3866.05	5.988944	3642.975	9.2486	6.90E-05	0.000305	2.891652	Sinapic ac	Sinapinic	86.3	2.717	224.067	C11H12O	530-59-6	6	-	C00482	ko01100//	Biosynthe	Metabolism
NEG0012	0.961604	0.56106	0.375142	0.286045	1.339305	0.338674	5.371004	6.586985	2.197072	4.441616	10.02919	7.106973	1	5.955473	2.574216	0.00067	0.001736	0.107149	Chorismi	5-[(1-Carboxyvinyl)(		1.558	226.0465	C10H10O	617-12-9	5	Carboxyli	C00251	ko01100//	Amino ac	Metabolism
NEG0012	6.774254	6.236122	7.344458	6.059055	6.862117	8.17751	12.46995	12.05579	15.01903	11.35467	11.50182	12.20376	6.908919	12.43417	0.847778	5.22E-06	4.97E-05	0.106277	Porphobi	Porphobilinogen		1.734	226.094	C10H14N	487-90-1	6	Organoni	C00931	ko01100//	Global an	Metabolism
NEG0012	72.79534	62.34047	99.06088	67.91586	56.31019	85.52356	96.1452	134.6783	72.94015	115.9944	88.8917	154.2122	73.99105	110.477	0.578323	0.025989	0.039608	0.22911	Allixin			3.988	226.1192	C12H18O	125263-70-	5	Pyrans	-	-	-	-
NEG0012	16.76176	12.9907	18.48934	13.44907	12.61366	18.06017	21.89043	26.8246	20.12569	16.64001	20.41734	25.05469	15.39412	21.82546	0.503633	0.006018	0.010918	0.107971	Myristoleic acid			9.103	226.1918	C14H26O	544-64-9	6	Fatty Acy	-	-	-	-
NEG0012	410.6982	263.946	475.5429	312.9473	132.9849	305.4896	1461.535	799.1676	1342.286	771.2466	948.8319	1195.319	316.9348	1086.398	1.777294	0.000128	0.000508	1.283754	Traumatic Acid			4.514	228.1348	C12H20O	6402-36-4	5	Fatty Acy	C16308	ko01110//	Global an	Metabolism
NEG0012	164.0424	146.3506	204.3684	148.2758	149.7432	184.7917	243.6198	187.5937	280.1685	209.2505	231.4683	315.3431	166.262	244.574	0.556812	0.00439	0.008322	0.378246	Leucylpro	Leucylpro	60.5	1.855	228.1461	C11H20N	6403-35-6	5	Carboxyli	-	-	-	-
NEG0012	78.37663	72.84875	82.22827	71.46141	85.07307	93.04698	96.30197	99.14249	83.10305	84.1605	112.4627	133.5466	80.50585	101.4529	0.333644	0.032694	0.047183	0.191742	Myristic a	Myristic a	99.5	9.884	228.2075	C14H28O	544-63-8	6	Fatty Acy	C06424	ko01100//	Global an	Metabolism
NEG0012	0.178845	0.513161	0.862482	1.640066	0.086469	2.227182	0.117731	0.107956	0.100697	0.097782	0.11716	0.140612	1	1	0	0.043594	0.06084	0.035729	Cyclobras	160492		6.884	234.0274	C11H10N	105748-58-	4	Indoles ar	-	-	-	-
NEG0013	297.3331	215.3218	460.2675	338.6504	174.0956	266.6278	150.08	161.1152	131.0737	154.0597	158.2814	164.571	292.0494	153.1968	-0.93083	0.007415	0.013263	0.484577	7-Hydroxyflavone			0.82	238.0675	C15H10O	6665-86-7	18	-	-	-	-	-
NEG0013	49.92934	57.79014	96.98521	67.3524	24.02465	66.90415	51.93237	27.0509	29.82977	54.26254	39.17267	71.30543	60.49765	45.59228	-0.40809	0.240427	0.278427	0.109142	7-Hydroxyflavanone			0.772	240.0827	C15H12O	NA	16	-	-	-	-	-
NEG0013	20.85644	15.05231	18.65692	17.50349	29.95397	23.50997	95.86012	98.44716	86.0228	59.79416	96.78893	79.75289	20.92218	86.11101	2.041164	1.37E-06	2.37E-05	0.38561	Genipinic	[Similar to: (+)-Syrin		2.044	242.0778	C11H14O	NA	5	Carboxyli	-	-	-	-
NEG0013	40.00465	318.1763	35.18149	31.32766	164.2228	428.4217	28.81847	18.77393	13.69669	15.63838	17.85172	24.34969	169.5558	19.85481	-3.0942	0.055497	0.074792	0.441116	Thymidir	1-(2-Deoxypentofura		1.654	242.0892	C10H14N	50-89-5	4	Pyrimidir	C00214	ko01100//	Global an	Metabolism
NEG0013	4.367217	3.991991	5.380694	3.34747	3.162505	3.496863	9.73591	15.04832	10.43743	15.86718	17.52521	29.72573	3.95779	16.38996	2.050046	0.001863	0.004127	0.155392	Lapachol			0.714	242.0978	C15H14O	84-79-7	14	-	-	-	-	-
NEG0013	28.70991	27.08454	31.89459	31.36922	33.14641	32.19822	46.67275	42.52024	41.04928	39.75437	35.66868	43.80812	30.73382	41.57891	0.436025	0.000129	0.000508	0.147998	Pentadecy	Pentadeca	98.6	10.265	242.2233	C15H30O	1002-84-2	5	Fatty Acy	-	-	-	-
NEG0013	13.73624	48.3722	9.585612	7.271066	10.51154	51.70135	2.547336	5.331777	4.72268	6.006915	3.910331	2.505903	23.52967	4.170824	-2.49608	0.045076	0.062679	0.163024	1-O-Galloylglycerol			3.471	244.0599	C10H12O	87087-60-3	6	Benzene a	-	-	-	-
NEG0013	373.3721	298.6572	411.2691	330.6427	236.8753	376.5662	669.7914	623.5683	560.2811	603.5338	582.7936	775.6573	337.8971	635.9376	0.912301	2.68E-05	0.000149	0.800932	Uridine	URIDINE		1.251	244.0683	C9H12N2	58-96-8	5	Pyrimidir	C00299	ko01100//	Global an	Metabolism
NEG0013	212.3485	132.9084	222.2499	211.129	111.8061	166.3139	22.74978	27.68749	22.48963	27.03317	21.33463	34.68112	176.126	25.99597	-2.76025	1.34E-05	9.66E-05	0.566052	Leucyl-le	Leu-Leu		2.15	244.1777	C12H24N	3303-31-9	4	Carboxyli	-	-	-	-
NEG0013	149.9309	130.1975	169.954	126.3664	118.0769	168.5943	189.2703	197.1288	183.6589	141.2786	156.0654	227.1444	143.8533	182.4244	0.3427	0.03143	0.046056	0.247286	Leucyl-As	Leucylasparagine		1.732	245.1364	C10H19N	NA	4	Carboxyli	-	-	-	-
NEG0014	225.3429	206.2619	270.4011	198.4487	208.1456	266.6048	202.6591	178.0598	231.3072	179.4034	186.2694	216.2308	229.2008	198.9883	-0.20393	0.082679	0.108623	0.192721	Aspartyl-	asp-leu		1.836	246.1204	C10H18N	NA	4	Carboxyli	-	-	-	-
NEG0014	161.806	117.2767	218.3287	164.2471	111.6442	187.7663	57.73383	107.086	66.17523	98.74554	63.80665	125.7742	160.1782	86.55357	-0.88801	0.004488	0.008465	0.370478	Dibutyl m	Dibutyl malate		2.661	246.1456	C12H22O	2385-79-7	4	Hydroxy	-	-	-	-
NEG0014	584.5534	744.189	530.9733	430.3133	859.1746	699.0363	11008.88	8026.999	6916.791	6830.832	9868.954	8543.247	641.3733	8532.618	3.733752	3.91E-07	9.32E-06	4.304076	Norambr	4-tert-Octylphenol m		8.738	250.192	C16H26O	564-20-5	5	Naphthof	-	-	-	-
NEG0014	85.32209	76.60948	102.938	77.67161	76.73057	87.67371	38.8821	38.89147	29.64608	33.56559	34.17869	37.10787	84.49091	35.37863	-1.25592	5.96E-07	1.30E-05	0.324891	Alanyl-Ty	MFCD03548046		1.864	252.11	C12H16N	3061-88-9	3	Carboxyli	-	-	-	-
NEG0014	45.41329	34.13014	51.68869	42.23243	19.17233	51.70611	200.113	179.0881	368.8819	137.367	204.2024	221.0476	40.72383	218.45	2.423358	0.000287	0.000853	0.584813	Benzoyl n	2-(Benzoyloxy)-3-hy		2.336	254.0416	C11H10O	65621-34-3	4	Benzene a	-	-	-	-
NEG0014	3.221384	1.162328	0.701337	0.133097	0.457847	0.63003	2.363383	0.944925	1.165221	0.273447	0.757311	0.456216	1.051004	1	-0.07177	0.918292	0.928035	0.002177	Daidzein	Daidzein	98.8	5.621	254.0568	C15H10O	486-66-8	4	Isoflavon	C10208	ko01110//	Biosynthe	Metabolism
NEG0014	351.7435	193.7958	354.4121	246.0135	176.905	303.6321	1276.043	952.8332	959.0274	797.6524	1043.977	851.3761	271.0837	980.1515	1.854267	2.94E-06	3.51E-05	1.261979	cis-9-paln	Palmitolei	79.8	10.513	254.2233	C16H30O	373-49-9	5	Fatty Acy	C08362	ko00061//	Lipid met	Metabolism
NEG0014	54.66905	45.82526	68.43445	50.14405	68.86668	58.45675	0.931559	0.413766	0.376986	0.345585	0.449924	0.491413	57.73271	1	-5.85132	3.95E-08	3.76E-06	0.355465	piscidic acid			1.458	256.0571	C11H12O	35388-57-9	4	Phenylpr	-	-	-	-
NEG0014	1642.055	1431.589	1722.109	1340.725	1172.289	1510.81	2710.929	2525.108	2333.803	2001.296	2441.498	2463.262	1469.93	2412.649	0.714871	2.22E-05	0.000136	1.458167	Palmitic a	Ethyl myr	99.6	10.924	256.2389	C16H32O	21096	5	Fatty Acy	C00249	ko01100//	Global an	Metabolism
NEG0014	0.455907	0.32235	0.173792	0.161662	0.312555	0.252374	0.148451	0.174052	0.363989	0.290469	0.14211	0.176955	1	1	0	0.296594	0.336317	0.007926	N-Lauroy	N-lauroylglycine		7	257.1979	C14H27N	NA	5	Carboxyli	-	-	-	-
NEG0015	2.394841	0.632792	1.59013	1.156318	0.957749	1.19896	625.3229	497.1641	345.3427	347.5287	622.1175	466.1201	1.321798	483.9327	8.51616	2.53E-06	3.14E-05	1.062267	6-Phosph	Methyl (2E)-3-[4-(su		2.301	258.0187	C6H11O9	2641-81-8	18	Organoox	C01236	ko01100//	Carbohyd	Metabolism
NEG0015	122.8561	107.0328	134.3539	112.963	104.9763	135.3794	90.81273	98.95594	89.28787	82.76254	79.13651	132.2831	119.5936	95.53978	-0.32397	0.030755	0.045418	0.177272	Leucyl-Gl	leu-gln		1.739	259.1521	C11H21N	NA	4	Carboxyli	-	-	-	-
NEG0015	703.9063	403.8728	564.366	377.1133	489.5088	595.8425	724.8357	488.0092	476.2953	312.7834	671.4797	401.3737	522.4349	512.4628	-0.0278	0.905412	0.917452	0.035936	D-fructos	D-Fructos	74.5	0.828	260.0285	C6H13O9	15978-08-2	4	Carbonyl	C01094	ko01100//	Amino ac	Metabolism
NEG0015	52.909	49.09446	60.88997	44.47983	43.69197	53.59653	26.18215	30.67043	34.46856	24.79543	23.86124	38.89272	50.77696	29.81175	-0.76829	0.000163	0.000554	0.202721	gamma-C	gamma-Glutamylleu		1.74	260.1361	C11H20N	2566-39-4	3	Carboxyli	-	-	-	-
NEG0015	0.114772	0.093775	0.126539	0.100033	0.072515	0.112984	12.79215	6.269129	3.058214	5.762079	7.732916	6.297277	1	6.985295	2.804321	0.000391	0.001112	0.122532	Mannitol	1-phosphate		2.976	262.0441	C6H15O9	15806-48-1	4	Organoox	C00644	ko00051//	Carbohyd	Metabolism
NEG0015	364.942	247.3937	461.4422	310.488	254.6113	255.0314	517.9658	559.7096	267.8184	337.7187	351.7286	360.75	315.6514	399.2819	0.339075	0.178906	0.215706	0.30755	Ascorbala	4,5-Dihydroxy-4-(2,3		0.841	263.0629	C9H13NO	41679-87-2	4	Carboxyli	-	-	-	-
NEG0015	14.04713	6.57289	19.09419	17.47456	7.530607	7.970841	10.07526	7.296306	8.704955	7.882364	5.293209	7.645214	12.11504	7.816218	-0.63226	0.094617	0.121821	0.072488	Thiamine			0.755	264.1034	C12H16N	59-43-8	4	Diazines	C00378	ko01100//	Folding, s	Environmental Information Processing;Genetic Information Processing;Metabolism
NEG0015	23.02866	20.98208	30.55892	21.90445	16.47549	20.14395	2.117217	2.793306	2.148443	2.470975	2.228211	2.651472	22.18226	2.401604	-3.20734	1.16E-06	2.21E-05	0.206483	Subaphyl	Vorinostat		2.006	264.1464	C14H20N	501-13-3	4	Cinnamic	C10497	ko00330//	Amino ac	Metabolism
NEG0015	289.2425	255.2854	376.7105	226.6623	265.7637	300.457	255.6345	238.9819	201.8839	192.8576	306.3916	356.0188	285.6869	258.6281	-0.14356	0.433776	0.467102	0.07462	Xanthoxi	Dodecyl s	99.8	10.939	266.1539	C15H22O	NA	7	-	C13454	ko00906//	Metabolis	Metabolism
NEG0015	146.1239	142.8585	181.1628	117.8115	270.54	142.6578	243.7243	310.6647	218.2534	209.4327	264.7086	408.024	166.8591	275.8013	0.724999	0.016003	0.025945	0.446868	Adenosin	Adenosin	97.9	1.376	267.0955	C10H13N	58-61-7	4	Purine nu	C00212	ko01100//	Global an	Metabolism
NEG0016	79.81697	81.47707	105.6285	33.89904	112.5573	79.45529	60.34604	74.69559	31.47852	107.4178	117.4781	173.5675	82.13903	94.16393	0.197107	0.617082	0.64947	0.091219	Inosine	2-(alpha-D-mannosy		0.938	268.0782	C10H12N	58-63-9	9	Purine nu	C00294	ko01100//	Global an	Metabolism
NEG0016	2.538715	0.938078	0.650082	0.514229	0.264842	0.34757	0.446335	0.875581	0.115004	0.138332	0.195697	0.372454	1	1	0	0.186785	0.221009	0.019727	Imperato	(3R)-8-hyd	77.7	6.603	270.0882	C16H14O	482-44-0	3	Coumarin	-	-	-	-
NEG0016	62.24558	59.97392	52.03592	36.76367	69.31633	67.94924	160.4366	86.9312	90.91352	80.09952	95.66693	99.71384	58.04744	102.2936	0.817411	0.00656	0.01179	0.296724	Heptadec	Heptadecanoic acid		11.326	270.2547	C17H34O	506-12-7	4	Fatty Acy	-	-	-	-
NEG0016	12.14289	30.80253	21.80003	22.42203	13.27116	34.23411	9.594211	21.58253	45.7189	82.52815	42.82442	42.82816	22.44546	40.84606	0.863773	0.120043	0.151446	0.107524	Tridecanc	N-Tridecanoylglycin		5.425	271.2137	C15H29N	NA	4	Carboxyli	-	-	-	-
NEG0016	3.613537	2.064741	3.775253	1.963006	1.214668	2.355165	18.88056	13.97453	6.887235	19.00882	17.17426	18.09604	2.497728	15.67024	2.649339	5.11E-05	0.000244	0.167822	Naringen	Naringeni	64.9	4.233	272.0674	C15H12O	480-41-1	3	Flavonoic	C00509	ko01100//	Biosynthe	Metabolism
NEG0016	79.06746	70.4562	107.896	88.59301	27.23722	103.359	74.52865	142.405	112.6667	178.698	133.6681	204.7359	79.43483	141.117	0.829049	0.020283	0.031933	0.28208	Arbutin			2.463	272.0886	C12H16O	497-76-7	3	Organoox	C06186	ko00010//	Carbohyd	Metabolism
NEG0016	7.716697	7.162881	10.53527	9.018597	7.72939	9.207407	25.01957	19.29882	22.09053	34.00161	24.88296	23.81654	8.561708	24.85167	1.537372	1.48E-05	0.000103	0.177424	16-Hydro	16-Hydroxyhexadec		9.335	272.234	C16H32O	NA	4	Fatty Acy	-	-	-	-
NEG0016	363.8171	359.1414	448.065	356.9814	239.6493	405.7519	312.417	260.5602	357.3073	204.3696	246.5917	361.7151	362.2343	290.4935	-0.31842	0.092373	0.120117	0.271928	6-Phosphogluconic acid			1.167	276.0278	C6H13O1	53411-70-4	11	Organoox	C00345	ko01100//	Carbohyd	Metabolism
NEG0016	25.31317	26.73613	34.30162	25.32536	16.30344	25.19786	47.3342	41.36968	42.10572	63.34651	55.21023	54.4455	25.5296	50.63531	0.987973	0.000137	0.000511	0.219596	Gamma	Glutamylglutamic aci		0.825	276.0944	C10H16N	1116-22-9	4	Carboxyli	-	-	-	-
NEG0016	0.821734	0.725914	1.104616	0.644766	0.360637	5.272732	28.22319	7.625729	6.793135	17.32393	27.15079	12.78486	1.4884	16.65027	3.483712	0.003008	0.006128	0.171911	Panteth ei	(+/-)-pantetheine		1.999	278.129	C11H22N	496-65-1	3	Carboxyli	C00831	ko01130//	Biosynthe	Metabolism
NEG0017	3.111644	5.112828	7.730894	6.823164	3.162243	2.718642	9.330684	7.660484	3.491013	3.494175	4.558372	7.07795	4.776569	5.935446	0.313382	0.400627	0.43736	0.034925	Panaxaco	1-Isopropyl-4,8-dim		7.196	278.187	C17H26O	106828-96-	4	Fatty Acy	-	-	-	-

NEG0017	7.166736	7.570067	9.158924	7.504921	6.334216	8.19152	75.90867	48.18593	43.69478	76.08661	61.40489	82.24203	7.654397	64.58715	3.076886	5.76E-06	5.35E-05	0.352948	α-Linolen	Oleic acid	97.5	7.983	278.2232	C18H30C	463-40-1	4	Fatty Acy	C06427	ko01100//	Global an	Metabolism
NEG0017	70.24949	44.34879	74.06522	45.06057	53.19739	73.08882	54.18128	46.7853	53.34926	37.2473	47.21703	52.88809	60.00171	48.61138	-0.30371	0.101244	0.129442	0.109156	Asparagi	asn-phe		1.87	279.1208	C13H17N	NA	3	Carboxyli	-	-	-	-
NEG0017	96.71066	68.82228	95.7855	69.28034	78.13914	100.2918	50.88267	57.89241	74.02191	48.48018	42.63095	72.15631	84.83829	57.6774	-0.55671	0.006398	0.011553	0.21804	L-beta-as	α-Asparty	94.2	1.974	280.1049	C13H16N	NA	3	-	-	-	-	-
NEG0017	656.8136	878.402	732.4917	502.5731	724.9079	731.5367	4516.334	3197.62	2629.475	2662.461	3901.907	3095.937	704.4542	3333.955	2.242657	6.35E-06	5.55E-05	2.473759	Linoleic a	9(Z),11(E)	99.6	10.363	280.2389	C18H32C	60-33-3	4	Fatty Acy	C01595	ko01100//	Global an	Metabolism
NEG0017	14.75651	12.21739	15.03738	11.47413	18.4322	19.0542	11.93606	14.18431	12.19137	7.767031	17.00294	16.24955	15.16197	13.22188	-0.19753	0.32449	0.360439	0.02841	4-Hydrox	N-4-hydroxyphenyl		2.595	281.0888	C13H15N	1029120-37	4	-	C05595	ko00350//	Amino ac	Metabolism
NEG0017	1.623689	1.269781	2.076127	1.286184	1.536103	1.379248	8.904852	4.203678	1.59492	6.105951	8.1763	7.941102	1.528522	6.154467	2.009497	0.002473	0.005264	0.098365	Xylobiose			1.313	282.0938	C10H18C	6860-47-5	5	Organoox	C01630	ko02010//	Membran	Environmental Information Processing
NEG0017	63.28544	36.02274	72.31995	61.26745	88.30336	58.31136	13.03003	17.1801	11.52009	5.691522	9.365151	7.843796	63.25172	10.77178	-2.55385	2.69E-05	0.000149	0.329809	Artemisir	NP-00519	88.5	2.378	282.1458	C15H22C	63968-64-9	3	-	-	-	-	-
NEG0017	0.192895	1.623766	0.209897	24.69781	3.580558	0.193955	258.7044	61.56656	50.93956	156.207	127.8603	175.8128	5.083147	138.5151	4.768177	0.001838	0.004119	0.518997	(6E,8R,10	(6E,8R,10Z)-8-Hydr		5.526	282.1819	C16H26C	2190485-67	4	-	-	-	-	-
NEG0017	1572.759	1195.826	1508.623	1141.571	1508.865	1190.035	2317.194	2378.341	1762.896	1421.616	2256.505	1843.868	1352.947	1996.737	0.561539	0.004236	0.008069	1.153291	Oleic Aci	Oleic acid	99.6	11.085	282.2546	C18H34C	112-80-1	4	Fatty Acy	C00712	ko01040//	Lipid met	Metabolism
NEG0018	0.180008	0.174853	0.21713	0.177545	0.134243	0.203799	4.369016	2.331136	0.419844	1.52112	4.742001	1.195912	1	2.429838	1.28086	0.010738	0.018265	0.065702	Rhein			4.322	284.0311	C15H8O6	478-43-3	3	Anthrace	-	-	-	-
NEG0018	1268.851	1055.515	1535.72	885.3814	995.0433	1156.678	1722.701	1353.018	1642.729	1184.889	1632.908	1885.62	1149.531	1570.311	0.450004	0.013535	0.022387	0.893911	Stearic ac	Stearic aci	99.7	12.227	284.2702	C18H36C	21128	4	Fatty Acy	C01530	ko01040//	Lipid met	Metabolism
NEG0018	152.731	131.5347	185.2524	137.2355	86.4521	201.6717	122.1226	67.59115	91.96106	98.34079	74.75177	129.3235	149.1462	97.34847	-0.6155	0.024596	0.03794	0.276829	Morphine	KL2880000		1.585	285.1314	C17H19N	57-27-2	17	Morphina	C01516	ko01100//	Biosynthe	Metabolism
NEG0018	36.94618	6.149897	3.312689	7.598541	5.330326	5.872137	684.2088	364.2357	93.46006	236.4786	681.1965	205.3818	10.86829	377.4936	5.118255	0.005148	0.009476	0.858893	Luteolin	Luteolin	98	4.32	286.0466	C15H10C	491-70-3	3	Flavonoic	C01514	ko01100//	Biosynthe	Metabolism
NEG0018	0.76553	0.543553	0.321167	0.198719	0.530192	0.232576	5.699099	9.496163	5.887031	1.00665	1.923043	1.375361	1	4.231224	2.081075	0.020038	0.031679	0.075189	Kaempfe	(4E)-5-(4-Methoxypl		2.389	286.05	C15H10C	520-18-3	7	Flavonoic	C05903	ko01100//	Biosynthe	Metabolism
NEG0018	3.69022	2.097313	4.215035	2.91102	2.054461	4.50961	28.16202	20.53866	14.34352	32.50275	30.58625	35.23824	3.246276	26.89524	3.050493	2.78E-05	0.000151	0.22512	2,4,6-trihy	2,4,6-Trih	90.9	3.267	288.0622	C15H12C	NA	4	-	-	-	-	-
NEG0018	10.78834	6.82068	13.35248	10.33732	4.882287	10.28897	21.69304	22.98888	36.82531	28.39237	27.82467	32.53307	9.411681	28.37622	1.592158	2.97E-05	0.000157	0.192105	Phlorin	1,5-Anhydro-1-(2,4,6		2.51	288.0835	C12H16C	28217-60-9	3	Carbohyc	-	-	-	-
NEG0018	53.42715	21.40374	29.93347	28.93986	16.83986	32.1811	7.131033	13.33038	11.16172	8.739515	6.607391	17.24162	30.4542	10.70194	-1.50877	0.004543	0.008495	0.184501	Atropine			1.691	289.1626	C17H23N	55-48-1	17	Tropane a	C01479	ko01110//	Biosynthe	Metabolism
NEG0018	50.27501	47.63635	51.00212	26.76002	45.9162	55.7348	56.13708	30.22381	27.87081	7.848779	44.44546	12.96611	46.22075	29.91534	-0.62766	0.085296	0.111676	0.116261	Sedoheptulose 7-phosphate			0.851	290.039	C7H15O1	2646-35-7	4	Organic o	C05382	ko01100//	Carbohyc	Metabolism
NEG0018	270.9691	167.8493	352.4793	246.5144	122.8294	196.0657	29.2257	21.13495	28.54351	28.31574	14.74425	35.95829	226.1179	26.32041	-3.10282	0.000138	0.000511	0.635594	Catechin			0.824	290.0755	C15H14C	154-23-4	12	Flavonoic	-	-	-	-
NEG0019	6.366384	3.878641	10.04417	7.4748	3.473006	4.158796	4.411216	2.458108	2.014476	3.048828	3.174105	2.707181	5.8993	2.968986	-0.99057	0.023662	0.036648	0.06439	N-gamma	gamma-Glutamyl-S-		1.56	290.0926	C11H18N	NA	3	Carboxyli	-	-	-	-
NEG0019	6.429654	7.192065	7.607952	4.772012	5.045358	8.809763	71.79119	36.22241	26.82836	60.47048	63.4774	53.58636	6.642801	52.0627	2.970387	7.44E-05	0.000322	0.313102	Argininos	Argininosuccinic aci		0.787	290.1213	C10H18N	2387-71-5	4	Carboxyli	C03406	ko01100//	Amino ac	Metabolism
NEG0019	41.95844	31.79243	46.35224	35.98348	37.12665	45.11826	20.76882	22.40065	23.37055	19.41853	19.04911	30.59263	39.72192	22.60005	-0.81361	0.00015	0.000535	0.18469	Glutamin	phe-gln		1.877	293.1366	C14H19N	92327-75-8	3	Carboxyli	-	-	-	-
NEG0019	94.08712	71.08546	104.6938	72.94247	75.28319	89.78859	52.098	51.38731	53.72438	41.03178	40.46613	56.46952	84.64678	49.19619	-0.78291	0.000195	0.000635	0.263202	Tyrosyl-F	4-Hydroxyprolyltyr		1.953	294.1205	C14H18N	936346-34-	3	Carboxyli	-	-	-	-
NEG0019	28.69405	17.86211	29.38618	18.12975	8.834593	29.31031	15.77669	15.51224	6.224062	12.22648	14.64128	10.39236	22.03617	12.46219	-0.82232	0.029407	0.043973	0.116772	Leucyl-Ty	Leucyltyrosine		1.982	294.157	C15H22N	NA	3	Carboxyli	-	-	-	-
NEG0019	186.5005	292.1907	303.4504	223.8746	46.90186	295.602	295.3478	229.7635	145.5064	45.97767	276.6022	433.0225	224.7533	237.7033	0.08082	0.851952	0.874916	0.120669	6-Ginger	(+)-[6]-Gingerol		5.899	294.1821	C17H26C	58253-27-3	3	Phenols	C10462	ko01110//	Biosynthe	Metabolism
NEG0019	6.078651	3.722301	4.642732	2.834608	3.229115	3.043336	7977.722	4407.325	2176.975	8218.703	13636.56	8771.049	3.925124	7531.388	10.90596	0.000879	0.002148	3.943806	Phaseolic	Phaseolic acid		2.381	296.0519	C13H12C	53755-04-7	4	Fatty Acy	-	-	-	-
NEG0019	30.75828	25.84035	5.977138	13.26635	21.50258	29.08455	49.26235	51.20862	30.29015	26.66511	46.75801	29.83343	21.07154	39.00294	0.888287	0.014136	0.023215	0.1792	Avenolei	13-HODE		8.739	296.2338	C18H32C	177931-23-	5	Fatty Acy	C14762	ko00591//	Lipid met	Metabolism
NEG0019	2.219429	1.552006	3.932862	4.118761	0.905746	1.565544	5.033606	4.907624	2.379596	3.652256	8.824862	4.983873	2.382391	4.963636	1.058987	0.032114	0.0467	0.065667	12,13-EpC	(+/-)12(13)	90.4	9.273	296.2338	C18H32C	NA	4	Fatty Acy	C14826	ko01100//	Global an	Metabolism
NEG0019	1084.382	376.0714	1209.859	960.6756	212.5075	483.3071	62.98755	33.74907	46.26303	48.82149	65.17639	66.12558	721.1338	53.85385	-3.74315	0.002805	0.005871	1.091569	Ostruthin	Ostruthin	91.1	9.881	298.1589	C19H22C	148-83-4	6	-	-	-	-	-
NEG0020	37.67548	16.2728	42.2036	25.11605	3.172795	13.12541	11.59078	11.42297	5.618085	6.206192	4.703693	7.453899	22.92769	7.832603	-1.54953	0.036172	0.051424	0.141773	Ricinoleic	NP-01154	80.1	8.258	298.2495	C18H34C	141-22-0	4	Fatty Acy	-	-	-	-
NEG0020	310.0604	467.395	378.3068	288.776	211.8812	475.7523	45.67304	59.15884	21.54202	26.40514	50.3134	27.53644	355.3619	38.43815	-3.20868	2.46E-05	0.000144	0.808512	4-(β-D-Gl	4-(beta-D-glucosylo		2.049	300.0834	C13H16C	NA	3	-	-	-	-	-
NEG0020	0.162629	0.126823	0.189611	0.141742	0.123573	0.159779	11.27633	3.989551	1.081982	6.933381	16.90656	4.986587	1	7.529066	2.912471	0.010049	0.017324	0.117508	Quercetir	Quercetin	92.7	3.699	302.0415	C15H10C	117-39-5	3	Flavonoic	C00389	ko01100//	Biosynthe	Metabolism
NEG0020	87.31686	61.65524	85.97424	55.56128	37.24431	56.46246	227.9785	93.56784	244.6646	115.8238	173.4265	184.3129	64.03573	173.2957	1.436287	0.001665	0.003776	0.465436	Tributyri	butyrin		3.992	302.1718	C15H26C	21920	3	Glyceroli	-	-	-	-
NEG0020	28.41584	22.40184	29.78536	26.55018	18.02519	26.75561	50.34377	55.08713	44.97931	44.16544	41.43487	53.85895	25.32234	48.31158	0.931958	1.25E-05	9.35E-05	0.220889	Nicotiana	nicotianamine		0.779	303.1416	C12H21N	34441-14-0	4	-	-	-	-	-
NEG0020	24.40819	15.64198	31.65818	44.34783	37.35004	21.50867	7.725434	18.13061	9.80184	6.95966	12.96238	13.9201	29.15248	11.58334	-1.33157	0.003766	0.007284	0.174458	Gallocate	(2S,3S)-2-(3,4,5-trihy		2.381	306.0764	C15H14C	970-73-0	7	Flavonoic	C12127	ko01110//	Biosynthe	Metabolism
NEG0020	31.01959	33.86139	31.5509	27.1092	32.76354	31.25094	231.2881	272.8309	216.5596	151.8654	300.779	242.34	31.25926	235.9438	2.916088	1.92E-06	3.01E-05	0.687577	Dihomo-g	8Z,11Z,14	99.3	10.624	306.2545	C20H34C	1783-84-2	4	Fatty Acy	C03242	ko01100//	Global an	Metabolism
NEG0020	0.317896	0.381844	0.354016	0.20323	0.597453	0.658469	13.76552	9.85859	7.496891	5.59607	26.41075	7.777458	1	11.81755	3.562859	0.004548	0.008495	0.150601	Warfarin			2.488	308.108	C19H16C	81-81-2	10	Coumarir	-	-	-	-
NEG0020	119.496	88.09926	187.068	135.8772	75.71307	135.9606	26.26884	21.97978	15.12223	12.27449	15.54835	27.03031	123.7024	19.704	-2.65031	8.57E-05	0.000359	0.462223	5-O-Meth	(6E,8E,10R,12Z)-10-I		6.924	308.1976	C18H28C	56005-10-8	3	Carbonyl	-	-	-	-
NEG0020	11.5196	10.52122	9.472119	7.489858	11.9942	13.12314	77.91631	65.19208	59.8662	65.49414	90.87107	64.56188	10.68669	70.65028	2.72488	2.00E-07	5.87E-06	0.36993	Eicosadie	11(Z),14(Z	99.8	11.287	308.2702	C20H36C	5598-38-9	4	Fatty Acy	C16525	ko01040//	Lipid met	Metabolism
NEG0021	329.0353	134.2257	540.8886	314.4985	3.249855	176.5314	0.746699	0.635639	0.451577	0.533532	0.666998	0.731901	249.7382	1	-7.96427	0.008511	0.014944	0.644477	Picrotin			4.308	310.1076	C15H18C	21416-53-5	7	Furopyra	-	-	-	-
NEG0021	28.11874	19.51789	25.36824	23.47015	19.1006	35.77665	46.73755	43.4814	56.33443	40.37076	30.29049	48.88024	25.22538	44.34914	0.81403	0.00142	0.003259	0.178899	Picrotin	Picrotin		2.594	310.1076	C15H18C	21416-53-5	7	Furopyra	-	-	-	-
NEG0021	0.354013	0.318333	0.545896	0.337768	0.29183	0.395315	359.2126	184.5967	94.15864	371.6351	545.9422	383.5205	1	323.1776	8.336184	0.000595	0.001574	0.819822	Caftaric acid			2.386	312.0471	C13H12C	67879-58-7	3	Cinnamic	-	-	-	-
NEG0021	137.9942	128.8712	170.6312	149.541	59.80887	150.9839	88.67703	87.05493	86.3221	78.44488	95.21338	108.5833	132.9717	90.71593	-0.55169	0.026603	0.040221	0.245174	Aflatoxin B1			0.863	312.068	C17H12C	1162-65-8	14	Coumarir	C06800	ko01130//	Global an	Metabolism
NEG0021	89.35521	67.79473	84.65343	57.073	95.75628	66.82007	90.42418	81.48114	73.18895	61.55285	91.63972	86.30954	76.90879	80.76606	0.070601	0.63093	0.662216	0.066241	Arachidic	Arachidic	94.9	14.331	312.3016	C20H40C	506-30-9	3	Fatty Acy	C06425	ko01040//	Lipid met	Metabolism
NEG0021	0.381	0.369435	0.42968	0.347886	0.326654	0.3835	112.0268	30.81023	14.83015	118.1589	217.0403	92.60004	1	97.57774	6.60848	0.008189	0.014445	0.421085	Geranyl diphosphate			1.745	314.0628	C10H20C	763-10-0	17	Fatty Acy	C00341	ko01100//	Global an	Metabolism
NEG0021	0.144532	0.119776	0.156463	0.136164	0.115458	0.154713	0.423685	0.131082	0.120841	0.350184	2.696023	0.161966	1	1	0	0.245551	0.282644	0.023444	Chlorochrymorin			1.609	314.0965	C15H19C	52525-23-2	14	-	-	-	-	-
NEG0021	19.62591	11.70389	21.4284	19.59299	9.502268	16.18832	26.69933	31.90476	47.52092	38.92444	37.43565	48.25281	16.3403	38.45632	1.234787	0.000244	0.000754	0.201758	Vanillosic	Avenein		3.053	314.0991	C14H18C	494-08-6	3	Organoox	-	-	-	-
NEG0021	8.118758	3.157793	3.067987	2.684976	3.192094	2.803943	546.0743	165.9165	140.2479	353.4508	630.0777	370.9663	3.837592	367.7889	6.582533	0.001085	0.002585	0.876978	Isorhamn	3-Methoxy	86.7	4.428	316.0571	C16H12C	480-19-3	3	Flavonoic	-	-	-	-
NEG0021	384.0193	380.1902	532.3883	404.873	279.3616	407.6439	280.456	262.9794	221.3925	233.8957	304.3527	274.9529	398.0794	263.0049	-0.59797	0.003378	0.006773	0.471525	Homoferreirin			0.822	316.099	C17H16C	482-01-9	13	Isoflavon	-	-	-	-
NEG0022	32.03805	24.89508	38.18526	11.57374	9.440357	30.1405	17.96196	12.89337	8.586451	18.89384	16.19547	26.34556	24.37883	16.81277	-0.53607	0.18558	0.220956	0.078241	Vanillolo	vanilloloside		2.28	316.1146	C14H20C	74950-96-2	3	Organoox	-	-	-	-
NEG0022	27.90042	13.29013	38.74768	34.9621	19.61975	23.83023	7.523739	9.687312	22.77055	13.99328	11.75813	36.34897	26.39172	17.01366	-0.63339	0.142283	0.174871	0.099884	Eseramin	[Similar to: N-[(1S,3a		2.698	318.1667	C16H22N	6091-57-2	7	-	-	-	-	-
NEG0022	30.25558	8.963906	35.20887	25.49553	8.167167	11.04297	26.46625	15.13592	15.18142	30.24156	22.75986	42.86769	19.85567	25.44212	0.357668	0.407985	0.442855	0.063094	Ourateacatechin			0.825	320.0859	C16H16C	NA	11	-	-	-	-	-
NEG0022	40.51289	19.68177	62.0803	63.52593	4.419444	33.88694	85.08991	45.71688	45.00482	31.0466	39.17807	68.87497	37.35121	52.48521	0.490756	0.258444	0.295697	0.125486	Gallocatechin-4beta-ol			0.879	322.0693	C15H14C	NA	1	-	-	-	-	-
NEG0022	2.314881	1.683403	7.830401	8.786149	1.283833	2.562364	1.212935	7.565264	0.977786	9.153976	1.348033	1.007366	4.076839	3.544227	-0.20198	0.80031	0.825464	0.023628	(-)-Phaseollin			2.415	322.1251	C20H18C	13401-40-6	14	Isoflavon	-	-	-	-
NEG0022	221.4238	314.6183	350.559	260.2651	186.2473	340.9659	69.50252	256.5722	311.6841	270.5027	377.4011	363.5971	279.0132	274.8766	-0.02155	0.939507	0.941979	0.033294	3'-CMP	Cytidine-3'-monoph		0.874	323.0504	C9H14N3	84-52-6	4	Pyrimidir	C05822	ko00240//	Nucleotid	Metabolism
NEG0022	25.22035	4.183142	32.03346	10.37735	55.5172	33.51599	32.00103	27.14928	16.32774	12.11918	43.30422	12.90232	26.80792	23.9673	-0.16159	0.759758	0.786598	0.014721	5'-CMP			1.235	323.0504	C9H14N3	63-37-6	4	Pyrimidir	C00055	ko01100//	Global an	Metabolism
NEG0022	28.77091	69.96042	49.83252	33.68805	168.0638	109.6591	137.9937	105.9267	99.05996	39.14028	226.8691	70.03905	76.66247	113.1715	0.561918	0.313401	0.34914	0.198622	Uridine n	Uridine 5'-monopho		0.86	324.0345	C9H13N2	58-97-9	4	Pyrimidir	C00105	ko01100//	Global an	Metabolism
NEG0022	2.011261	1.766728	2.318649	2.494999	0.920404	4.407393	1.448284	1.908846	2.032549	1.871907	1.722988	11.28827	2.319906	3.378807	0.542448	0.536306	0.567591	0.026251	(-)-Phaseollidin			2.417	324.1408	C20H20C	37831-70-2	14	Isoflavon	-	-	-	-
NEG0022	3.199371	1.36781	3.692549	2.073365	1.044279	1.690375	37.08614	22.0697	13.11455	45.24552	198.3541	60.76954	2.177958	62.77327	4.849102	0.055554	0.074792	0.305564	Fertaric a	Fertaric acid		2.393	326.0625	C14H14C	NA	3	Cinnamic	-	-	-	-
NEG0023	9.364253	5.99112	12.95669	10.37893	6.065491	7.422983	30.62479	18.24316	5.534416	25.96116	58.84473	25.19245	8.696579	27.40012	1.655662	0.028529	0.042962	0.178241	Neohespe	Neohesperidose		1.607	326.1199	C12H22C	17074-02-1	4	Organoox	-	-	-	-
NEG0023	12014.82	9601.274	9700.36	7144.828	14038.69	9166.504	39185.08	34222.39	33523.47	18193.7	42046.43	27319.31	10277.74	32415.06	1.657141	0.000122	0.000489	7.062311	Corchorif	Corchorifa	96.1	4.101	328.2235	C18H32C	95341-44-9	4	Fatty Acy	-	-	-	-
NEG0023	1.905661	1.933834	2.239146	1.239023	1.395654	7.104415	204.016	199.7098	190.4652	230.9638	212.3634	255.425	2.636289	215.4905	6.352972	9.48E-10	3.61E-07	0.6899	Laricitrin	Laricitrin		2.806	332.0494	C16H12C	53472-37-0	11	-	C12633	ko00944//	Biosynthe	Metabolism
NEG0023	3.815963	2.215384	5.095469	3.426829	1.912593	2.292361	32.6037	19.51225	15.81015	49.05393	32.84169	55.91582	3.126433	34.28959	3.455181	0.00071	0.001816	0.245057	beta-Gluc	Glucogallin		1.533	332.0731	C13H16C	13405-60-2	3	Tannins	-	-	-	-
NEG0023	81.19032	40.22027	108.1957	93.30168	17.76523	37.11354	0.35481	0.333489	0.299747	0.281133	0.333155	0.550993	62.96447	1	-5.97647	0.001704	0.003843	0.339236	5'-Hydroxy-3'-methoxysativan			1.731	332.1226	C18H20C	139906-02-	10	Isoflavon	-	-	-	-
NEG0023	112.3597	82.04865	238.2512	307.3145	80.17519	234.5806	192.8977	175.1622	166.9336	156.8754	183.8003	209.7516	175.7883	180.9035	0.041381	0.901188	0.915607	0.002911	AICA-Rib	AICA ribo	69.9	0.864	338.0644	C9H15N4	3031-94-5	5	Imidazole	C04677	ko01100//	Amino ac	Metabolism
NEG0023	40.69244	44.91894	64.56	50.11462	27.70972	44.63965	25.78454	31.27271	15.92015	35.48959	32.81484	29.49836	45.43923	28.46337	-0.67483	0.013573	0.022387	0.16518	Ovalitenc	Ascorbyl glucoside		0.861	338.0835	C19H14C	64280-22-4	13	Linear 1,3	-	-	-	-
NEG0023	438.8716	337.5671	136.7514	8.683323	255.6071	149.4346	3320.09	4630.701	3642.18	2458.023	2913.107	4587.511	221.1525	3591.935	4.021648	3.34E-06	3.86E-05	2.739842	4-p-Coun	NP-00058	91.9	2.117	338.0989	C16H18C	93451-44-6	3	-	-	-	-	-
NEG0023	39.89271	37.63223	37.47016	44.26152	160.6377	60.86408	46.84815	78.68033	52.7092	29.07102	62.7542	42.41944	63.45974	52.08039	-0.2851	0.59921	0.632407	0.057435	(-)-Glyceollin I			2.054	338.1202	C20H18C	57103-57-8	14	Isoflavon	C01701	ko01110//	Biosynthe	Metabolism
NEG0023	21.97717	20.72178	23.671	14.14939	18.99132	17.18597	24302.01	15048.89	6222.68	11776.16	20936.89	15915.95	19.44944	15700.43	9.65686	0.000138	0.000511	5.96945	Sinapoyl	Sinapoyl malate		2.726	340.0778	C15H16C	NA	4	-	C02887	ko00940//	Biosynthe	Metabolism
NEG0024	8.713464	8.341517	2.245516	0.127818	5.180739	2.674009	74.45044	102.133	80.34411	55.15883	64.77195	105.6899	4.547177	80.42471	4.144596	3.77E-06	4.11E-05	0.411662	3-Ketolactose			2.118	340.1038	C12H20C	15990-62-2	9	Organoox	-	-	-	-
NEG0024	20.50749	12.52724	19.54569	14.88533	9.244435	13.97766	95.00049	73.77012	150.6823	197.8981	98.62318	159.794	15.11464	129.2947	3.096645	0.000159	0.000554	0.449898	Linocinnamarin			3.55	340.1144	C16H20C	NA	4	Organoox	-	-	-	-
NEG0024	25.17007	17.6429	58.74181	28.60697	33.07606	16.64404	3.337456	1.944353	1.729381	1.837617	2.029049	3.268308	29.98031	2.357694	-3.66857	0.001383	0.003213	0.222967	(+)-Galba	methyl 2-O-alpha-L-		1.814	340.1355	C20H20C	61891-31-4	13	-	-	-	-	-
NEG0024	4.187705	2.903137	0.910569	1.139477	0.719734	1.166248	109.5552	73.8819	26.40216	132.5241	251.4087	154.8329	1.837811	124.7675	6.085109	0.002868	0.005907	0.492023	Caffeic acid 3-glucoside			1.946	342.0936	C15H18C	24959-81-7	4	-	-	-	-	-
NEG0024	620.4757	141.1806	699.438	323.3184	480.7448	634.4562	201.7752	440.0586	368.9875	190.5495	300.9189	266.363	483.269	294.7754	-0.71321	0.078684	0.103732	0.504319	Cellobios	D-(+)-CELLOBIOSE		0.973	342.1144	C12H22C	16462-44-5	5	Organoox	C00185	ko01100//	Carbohyc	Environmental Information Processing;Metabolism
NEG0024	2288.954	2342.643	736.6312	245.9141	1585.005	3826.566	853.5693	1078.429	812.8108	790.0856	1258.259	1249.005	1837.619	1007.026	-0.86774	0.148846	0.182348	0.898753	Sucrose	SUCROSE		0.794	342.1146	C12H22C	57-50-1	4	Organoox	C00089	ko01100//	Carbohyc	Environmental Information Processing;Metabolism
NEG0024	22.16361	22.21727	32.69564	24.37086	23.78225	24.96231	13.44808	11.63427	17.85044	13.82477	12.64672	17.56507	25.03199	14.49489	-0.78823	0.000267	0.000821	0.145037	Eupatilin			1.836	344.0877	C18H16C	855-96-9	5	Flavonoic	-	-	-	-
NEG0024	35.60271	41.45285	33.841	23.7741	93.01086	75.71207	39.49731	76.71944	79.11705	27.29079	44.62464	19.17414	50.5656	47.73723	-0.08304	0.85552	0.876218	0.031287	cGMP	Guanosine-3',5'-cycl		1.264	345.0459	C10H12N	7665-99-8	4	Purine nu	C00942	ko00230//	Nucleotid	Metabolism
NEG0024	1.129341	0.928951	0.813791	0.173722	3.166001	0.169701	1.570999	0.823639	0.214021	1.518613	1.314444	0.498332	1.063585	1	-0.08893	0.88734	0.906372	0.000267	Aucubin	Aucubin		1.947	346.1251	C15H22C	479-98-1	3	Prenol lip	-	-	-	-
NEG0024	3.59076	5.309443	3.869236	2.609692	6.342003	4.226102	46.40406	41.04692	33.94877	32.0777	46.48941	43.1648	4.324539	40.52194	3.228085	6.80E-08	4.32E-06	0.292256	Gibberellin A24			8.739	346.1796	C20H26C	NA	4	-	C11861	ko01110//	Global an	Metabolism
NEG0025	2.887901	3.293969	3.571417	2.41838	10.91441	2.749723	2.710284	6.341331	2.454523	2.078959	27.98532	3.148056	4.305967	7.453079	0.7915	0.487184	0.518483	0.051093	Adenosin	Adenosine 5'-monop		0.867	347.0616	C10H14N	61-19-8	4	Organoox	C00020	ko01100//	Global an	Metabolism
NEG0025	1.142834	1.050819	1.838948	0.335741	0.782324	0.859711	66.67387	57.64339	76.44354	72.74225	117.8677	104.2076	1.00173	82.5964	6.365514	6.45E-06	5.55E-05	0.426526	IMP			2.658	348.0442	C10H13N	131-99-7	8	Lactams	C00130	ko01100//	Global an	Metabolism
NEG0025	72.80932	33.37771	80.83239	75.31814	16.66614	55.11437	36.8442	54.61882	38.09165	12.44549	33.81059	32.28967	55.68635	34.6834	-0.68308	0.107958	0.137565	0.151884	Chlorogenoquinone			0.865	352.0798	C16H16C	24321-18-4	1	Organoox	-	-	-	-
NEG0025	60.05724	46.04181	78.37218	69.00311	63.30137	47.98707	9.861839	10.54557	12.35812	7.931742	13.1422	15.94997	60.7938	11.63158	-2.38588	2.55E-06	3.14E-05	0.322226	Citrunobin			2.385	352.1356	C21H20C	126026-23-	13	Linear 1,3	-	-	-	-
NEG0025	15.52978	11.60001	20.65141	12.70057	10.38432	16.21721	13.19378	10.8792	8.922493	9.348118	10.47462	12.29064	14.51388	10.85147	-0.41954	0.053589	0.073444	0.065582	Tetrahydroalstonine			8.295	352.1766	C21H24N	6474-90-4	5	-	C11682	ko01110//	Biosynthe	Metabolism
NEG0025	75.84158	63.30809	67.1416	50.05542	77.86959	78.84598	617.5537	561.685	465.2931	511.6511	671.9441	739.7429	68.84371	594.645	3.110632	1.95E-07	5.87E-06	1.108089	Chloroge	Chloroger	99.6	2.045	354.0937	C16H18C	327-97-9	3	Organoox	C00852	ko01110//	Biosynthe	Metabolism
NEG0025	3.790413	3.080822	1.976047	1.627581	4.230284	3.882526	647.8546	721.2135	175.9975	664.2417	667.9968	1110.741	3.097945	664.6743	7.745192	0.000279	0.00084	1.186825	5-Caffeoy	Neochlorc	95.9	1.901	354.0937	C16H18C	NA	3	-	-	-	-	-

NEG0025	5.931669	2.433829	6.959275	4.583903	1.703831	4.667658	255.476	384.7796	110.1962	359.0683	412.4579	212.636	4.380027	289.1023	6.044497	0.000138	0.000511	0.750228	Gentiopic	Gentiopicrin		2.285	356.1094	C16H20C	NA	3	-	C09782	ko01110//	Global an	Metabolism
NEG0025	29.04238	24.26137	39.86655	35.19597	26.23646	93.26877	4.808359	9.009579	3.120766	4.923177	5.448779	9.042274	41.31192	6.058822	-2.76945	0.008101	0.014356	0.253344	Tarennoside			1.57	358.1285	C16H22C	61081-59-2	5	-	-	-	-	-
NEG0025	21.18523	16.61467	23.44416	15.24762	20.4284	26.10698	39.11951	50.32583	51.12677	37.48829	39.38771	56.06465	20.50451	45.58546	1.152632	4.00E-05	0.000201	0.226791	Irigenin			1.373	360.0789	C18H16C	NA	15	-	-	-	-	-
NEG0026	1299.111	1384.553	1652.903	1575.551	735.1407	1546.294	453.0986	715.7467	503.264	455.446	702.7824	408.6478	1365.592	539.8309	-1.33895	0.000228	0.000723	1.292355	Rosmarinate			0.825	360.0886	C18H16C	537-15-5	11	Cinnamic	C01850	ko00350//	Amino ac	Metabolism
NEG0026	3.089618	10.37305	3.269216	2.369427	39.97343	17.90362	27.50016	55.22087	12.03218	15.50192	82.94994	23.05061	12.82973	36.04261	1.490213	0.098547	0.126419	0.179952	guanosin	Guanosine-5'-monop		1.239	363.0566	C10H14N	85-32-5	3	Purine nu	C00144	ko01100//	Global an	Metabolism
NEG0026	0.132513	0.119157	0.204337	0.126432	0.109624	0.270259	96.68077	66.81469	42.45671	94.29344	127.4487	129.5208	1	92.86919	6.537128	5.49E-05	0.000258	0.454129	XMP			2.378	364.0393	C10H13N	523-98-8	7	Purine nu	C00655	ko01100//	Global an	Metabolism
NEG0026	108.8743	21.83894	27.91023	17.40251	17.67975	37.63642	24.88975	58.76912	69.17704	50.26192	134.387	73.01606	38.55703	68.41681	0.827357	0.180634	0.217103	0.177735	Nervonic	Nervonic	99.5	12.901	366.3481	C24H46C	506-37-6	4	Fatty Acy	C08323	ko01040//	Lipid met	Metabolism
NEG0026	7.378822	3.317767	12.93833	10.68813	2.02382	4.333612	117.5556	359.8201	116.3582	156.1615	272.2344	157.2349	6.780081	196.5608	4.857529	0.000806	0.002007	0.59856	3-Feruloy	(1R,3R,4S,5R)-1,3,4-t		2.183	368.1092	C17H20C	62929-69-5	4	Alcohols	-	-	-	-
NEG0026	0.15295	0.607884	0.215287	0.146426	1.367911	0.171373	1.009917	3.820818	2.025029	0.767229	0.993237	1.719527	1	1.722626	0.78461	0.029535	0.043973	0.042608	Gomphidic acid			2.403	372.05	C18H12C	25328-77-2	5	Benzene	-	-	-	-
NEG0026	20.64478	11.1509	20.50795	14.4494	2.984008	9.391489	1.721307	1.444745	1.161004	1.807142	2.059852	2.157912	13.18809	1.725327	-2.93429	0.002135	0.004701	0.14232	Dihydroi	(2S,3S,4S,5R,6R)-6-(3		2.459	372.104	C16H20C	NA	4	-	-	-	-	-
NEG0026	55.59338	59.0121	56.18397	49.81753	49.63376	64.69164	36.03578	23.46146	28.18385	23.94302	24.73971	43.12598	55.82206	29.91497	-0.89997	7.25E-05	0.000318	0.220204	Syringin	beta-Syringin		2.888	372.1406	C17H24C	118-34-3	3	-	C01533	ko00940//	Biosynthe	Metabolism
NEG0026	20.66633	13.40251	22.1293	20.02275	10.6269	16.93159	68.84561	51.87954	38.46913	40.11489	70.8404	91.42215	17.29656	60.26196	1.800762	0.000532	0.001459	0.310813	Tetrahydrocurcumin			9.765	372.1587	C21H24C	36062-04-1	3	Diarylhep	-	-	-	-
NEG0026	0.604136	0.823927	1.118285	0.977146	0.59034	0.505784	0.820731	1.290083	0.458464	0.728813	1.027331	1.348463	1	1	0	0.330211	0.364668	0.013653	Saucernetin			6.551	372.1956	C22H28C	83377-13-3	5	-	-	-	-	-
NEG0027	4.084873	2.696258	4.610286	3.307222	3.952548	3.728154	28.52652	21.37512	23.05995	16.16943	24.73302	17.46256	3.72989	21.88777	2.55292	2.40E-06	3.14E-05	0.202541	Isodiospyrin			2.561	374.0837	C22H14C	20175-84-2	12	Dihydrof	-	-	-	-
NEG0027	33.28452	50.80991	26.81392	24.05264	41.95676	36.39719	1152.764	625.2573	509.3309	675.4099	963.8496	722.1253	35.55249	774.7895	4.445782	1.86E-05	0.000118	1.299667	Tripdiolide			9.739	376.1538	C20H24C	38647-10-8	4	-	-	-	-	-
NEG0027	12.07122	5.031771	7.917184	4.492282	6.571906	5.756415	165.6757	277.2899	312.0105	109.5851	136.8958	174.0097	6.973463	195.9111	4.81218	0.000187	0.000614	0.601566	Tomenin			2.394	384.1042	C17H20C	28446-08-4	3	Coumarir	-	-	-	-
NEG0027	6.800101	3.803796	6.620792	4.561408	4.234595	5.358973	36.17126	24.08617	17.83676	25.60903	22.71975	27.4517	5.229944	25.64578	2.293854	1.14E-05	8.86E-05	0.213105	Kanzonol N			1.942	384.1518	C22H24C	156250-71-	14	Isoflavon	-	-	-	-
NEG0027	278.0948	292.9824	287.2331	239.0825	272.7271	301.7686	123.184	91.48324	90.86914	81.78159	74.25886	139.975	278.6481	100.2586	-1.47472	1.43E-07	5.87E-06	0.611632	1-O-Sinapoyl-β-D-glucose			2.574	386.1199	C17H22C	NA	3	-	-	-	-	-
NEG0027	1032.072	1204.332	1823.308	1523.176	1041.079	1305.884	819.7032	533.3536	704.8892	964.1684	758.5771	875.6693	1321.642	776.0601	-0.76809	0.002854	0.005907	1.003662	Corchoio	3-Hydrox	70.4	2.284	386.192	C19H30C	NA	5	Fatty Acy	-	-	-	-
NEG0027	13.23524	26.46071	2.000554	5.338322	4.736492	24.13653	4.664665	4.757208	7.561734	0.251672	5.008123	0.164315	12.65131	3.734619	-1.76025	0.073237	0.096887	0.101857	Glucobrassicanapin			2.19	387.0612	C12H21N	19041-10-2	11	Organoox	-	-	-	-
NEG0027	1228.74	1224.216	1798.488	1134.784	599.8192	1619.766	574.2528	804.7621	527.8795	544.7756	815.8899	477.5005	1267.636	624.1767	-1.02211	0.005208	0.009539	1.060388	Betanidin			0.826	388.0837	C18H16N	2181-76-2	17	Indoles ar	C08539	ko01110//	Biosynthe	Metabolism
NEG0027	41.55143	2271.782	656.8764	53.66293	1547.357	3759.763	177.9529	124.3216	142.2341	176.0642	181.8552	199.6789	1388.499	167.0178	-3.05545	0.066559	0.088668	1.25881	Artemetir	D-(+)-Mal	70.3	1.007	388.12	C20H20C	479-90-3	10	Flavonoic	C00208	ko01100//	Carbohyc	Environmental Information Processing;Metabolism
NEG0027	1.92324	0.951845	1.915449	1.551947	5.262561	1.508755	30.90782	27.14657	6.374113	28.17544	26.37905	30.47121	2.185633	24.90903	3.510546	0.000145	0.00053	0.220343	Geniposi	geniposide		1.978	388.1357	C17H24C	24512-63-8	3	-	-	-	-	-
NEG0028	0.15936	0.397625	1.606581	0.146307	0.366194	0.285982	262.2007	197.839	94.08761	164.7928	211.4271	135.1106	1	177.5763	7.472295	2.60E-05	0.000148	0.625562	trans-Piceid			2.657	390.13	C20H22C	27208-80-6	4	Stilbenes	-	-	-	-
NEG0028	157.652	118.8171	169.8982	127.0968	94.72753	156.5845	274.8926	296.3011	238.4118	261.9076	223.9364	363.8734	137.4627	276.5538	1.00852	0.000147	0.000534	0.533515	Shanzhiside			7.692	392.1275	C16H24C	NA	11	-	-	-	-	-
NEG0028	2.806423	3.137369	2.590638	1.793854	3.899271	2.170119	13.70127	8.886291	10.31488	4.644016	13.09492	5.099473	2.732946	9.290142	1.765244	0.002173	0.004704	0.115947	Acidissiminol			4.414	393.2346	C25H31N	126006-00-	10	Prenol lip	-	-	-	-
NEG0028	17.15159	12.07882	20.15722	13.67948	12.36096	18.95923	14.61592	14.51091	11.3485	10.70577	12.21592	13.53167	15.73122	12.82145	-0.29507	0.094643	0.121821	0.056379	Rotenone	Rotenone		8.299	394.1432	C23H22C	83-79-4	3	Isoflavon	C07593	ko01110//	Biosynthe	Metabolism
NEG0028	6.038234	4.998485	6.253658	4.369736	9.518839	9.239214	20.13914	17.91634	10.73636	15.47504	14.44187	14.89164	6.736361	15.60007	1.211511	0.000224	0.000722	0.134597	gamma-M	gamma-mangostin		7.875	396.1586	C23H24C	31271-07-5	3	Benzopyr	-	-	-	-
NEG0028	0.632196	0.495994	0.685215	0.501311	0.454239	0.507687	2.246999	0.224848	0.205554	4.64577	0.812472	1.350445	1	1.581015	0.660851	0.164131	0.199153	0.026878	5-Hydrox	4-hydroxy-9-(4-hydr		3.293	400.1144	C21H20C	71802-04-5	3	Flavonoic	-	-	-	-
NEG0028	0.824835	1.378078	0.695259	0.254361	3.615816	1.129561	160.8573	256.5372	151.1022	27.94366	54.86306	52.20305	1.316318	117.2511	6.476949	0.00896	0.01566	0.43547	Shoyuflavone B			2.389	402.0603	C19H14C	190712-88-	3	Isoflavon	-	-	-	-
NEG0028	163.1455	25.84973	236.3382	190.9354	83.73522	246.7124	71.67222	111.2583	69.44873	43.75536	102.5832	55.96141	157.7861	75.77988	-1.05808	0.052125	0.071956	0.348335	Apigenin 7-xyloside			0.824	402.0983	C20H18C	NA	8	-	-	-	-	-
NEG0028	101.2314	53.94247	121.6293	86.60557	67.36516	72.60294	168.7529	162.8555	86.70727	231.5771	160.9621	289.2178	83.89614	183.3454	1.127888	0.007844	0.013965	0.410186	Osajin	N-[(1S)-4-Carbamim		2.868	404.1667	C25H24C	482-53-1	10	-	-	-	-	-
NEG0028	8.783878	8.591491	3.143998	0.132373	5.010642	5.437429	49.23806	56.28549	50.98084	33.57189	40.44614	67.02401	5.183302	49.59107	3.258137	4.53E-06	4.80E-05	0.317085	Lancerin			2.114	406.0858	C19H18C	81991-99-3	10	-	-	-	-	-
NEG0029	96.67537	54.86787	116.1387	76.14994	77.0209	60.83932	37.70682	31.58602	30.91035	43.46962	35.21717	46.74794	80.28202	37.60632	-1.0941	0.001317	0.003078	0.278064	Mammeisin			2.851	406.1741	C25H26C	18483-64-2	9	Neoflavoı	-	-	-	-
NEG0029	6.305302	6.68714	4.763146	3.261033	5.106913	4.489947	46.25796	24.53702	19.77458	17.36331	25.34606	18.51272	5.102247	25.29861	2.309853	0.001034	0.002479	0.206911	dADP			8.74	411.0331	C10H15N	326322	3	Purine nu	C00206	ko01100//	Global an	Metabolism
NEG0029	527.8798	207.7054	560.547	431.3845	90.97251	202.2059	37.93849	24.71555	35.49887	35.48235	29.42441	46.30672	336.7825	34.8944	-3.27075	0.003582	0.007071	0.724995	Patientoside A			10.716	412.0993	C19H21C	NA	16	Organoox	-	-	-	-
NEG0029	5.652428	3.494137	4.575265	5.372898	2.893584	4.995676	2.453577	1.735873	2.222008	2.596233	1.18015	3.688952	4.497331	2.312799	-0.95943	0.003102	0.006286	0.063116	Abscisic alcohol 11-glucoside			2.993	412.208	C21H32C	145153-00-	4	Prenol lip	-	-	-	-
NEG0029	0.177043	0.462305	0.205167	0.150529	0.186496	0.231651	10.71626	3.535235	1.412824	2.26788	2.549415	2.09411	1	3.762621	1.911738	0.032317	0.046816	0.077392	Puerarin	Puerarin		3.295	416.109	C21H20C	3681-99-0	4	Phenylpr	-	-	-	-
NEG0029	0.104919	0.102449	0.260435	0.101399	0.192998	0.114527	16.43943	10.04427	4.591417	8.477956	10.90926	8.433167	1	9.815916	3.295123	0.00012	0.000488	0.146129	Isorhapontin			2.985	420.1406	C21H24C	NA	3	-	-	-	-	-
NEG0029	345.6347	210.445	591.725	726.8319	140.5531	821.6741	95.98578	103.0951	146.6805	528.0166	72.11072	139.9063	472.8106	180.9658	-1.38555	0.055466	0.074792	0.727789	Polydine			4.072	422.1197	C20H22C	NA	3	-	-	-	-	-
NEG0029	22.78802	16.68089	25.74158	22.19832	16.01483	22.52372	15.31809	12.25201	12.77725	11.80152	11.88072	17.36042	20.99123	13.565	-0.6299	0.002165	0.004704	0.113531	Lamiide			2.212	422.1428	C17H26C	27856-54-8	0	-	-	-	-	-
NEG0029	5545.03	1356.353	4665.757	3092.205	34.84149	1205.045	4.899803	3.263087	4.930413	2.950014	3.861217	2.871048	2649.872	3.79593	-9.44725	0.0132	0.022104	2.024719	Picrasin C			11.013	422.2308	C23H34C	33804-89-6	0	-	-	-	-	-
NEG0029	121.6064	111.7278	143.4655	119.8117	89.87365	110.4529	66.23949	39.74041	49.74939	76.17957	62.93563	75.98578	116.1563	61.80505	-0.91027	0.000162	0.000554	0.326898	Neolinustatin			2.291	423.1727	C17H29N	72229-42-6	3	Carbohyc	-	-	-	-
NEG0030	154.6602	141.1639	185.4621	158.4977	110.4325	140.5809	87.87192	50.21524	68.04502	98.27561	75.09707	109.8602	148.4662	81.56084	-0.86419	0.000547	0.001488	0.354944	Grandidentatin			2.284	424.1658	C21H28C	15732-48-6	17	-	-	-	-	-
NEG0030	48.56477	30.2671	57.98162	49.73923	16.23373	39.08672	59.13896	34.83921	41.52321	82.03096	50.35348	69.03112	40.31219	56.15282	0.478142	0.125785	0.157646	0.118877	Carapanaubine			2.52	428.2028	C23H28N	1255-02-3	18	-	-	-	-	-
NEG0030	65.83852	54.65502	83.33473	65.3757	36.41627	48.55116	7.347667	7.856805	7.686027	8.407443	10.83855	8.225637	59.02857	8.393688	-2.81404	1.80E-05	0.000118	0.324318	Ononin			0.942	430.1301	C22H22C	486-62-4	8	Isoflavon	C10509	ko00943//	Biosynthe	Metabolism
NEG0030	3.495836	3.981343	4.230299	4.862053	6.443154	5.595188	8.983772	7.689787	6.01798	8.526364	7.176612	17.27248	4.767979	9.277832	0.96041	0.025124	0.038598	0.086662	Homofukinolide			2.301	430.2411	C25H34C	41059-96-5	12	Prenol lip	-	-	-	-
NEG0030	766.5685	544.2	860.4085	752.9593	500.2629	629.9455	405.7807	276.6794	368.8019	488.9781	389.3138	527.3108	675.7241	409.4774	-0.72265	0.002868	0.005907	0.692114	S-Furanopetasitin			2.286	432.1973	C24H32C	34335-97-2	0	Prenol lip	-	-	-	-
NEG0030	65.06202	71.49617	104.6546	76.07988	27.55954	74.59958	246.8205	157.0858	99.78458	230.2225	212.6548	160.4259	69.90864	184.499	1.40007	0.000934	0.002266	0.467986	Quercetin 3-arabinoside			0.859	434.0889	C20H18C	22255-13-6	9	Flavonoic	-	-	-	-
NEG0030	0.339097	0.621475	0.392963	0.288314	0.318198	0.427733	13.02166	3.787226	1.585782	2.83499	3.552892	2.807912	1	4.59841	2.201135	0.034245	0.04905	0.084715	Dihydrogenistin			3.294	434.1194	C21H22C	NA	4	Isoflavon	-	-	-	-
NEG0030	72.55912	92.17863	94.21392	67.25976	79.01202	99.77866	55.20586	364.3903	280.9666	249.6098	362.3615	71.36261	84.16702	230.6494	1.454375	0.026436	0.040128	0.414756	Taxifolin 3-arabinoside			0.837	436.0945	C20H20C	NA	14	Flavonoic	-	-	-	-
NEG0030	4.715561	3.443926	3.66815	3.354165	1.635377	3.821878	8.422889	5.716597	7.638202	12.32165	10.83641	15.3092	3.439843	10.04082	1.545463	0.00122	0.002868	0.111642	Kanzonol	1-Cyclohe	70	1.996	438.164	C25H26C	181476-22-	8	Isoflavon	-	-	-	-
NEG0030	156.7711	67.27799	146.3515	106.7662	29.13575	57.53409	19.5774	11.72515	22.42153	20.8633	19.7828	30.43195	93.97277	20.80036	-2.17563	0.005937	0.010822	0.347108	Ginkgolide C			11.345	440.1306	C20H24C	15291-76-6	2	Prenol lip	-	-	-	-
NEG0031	0.456908	0.416624	0.497457	0.39901	0.379176	0.471009	62.77013	38.45428	51.85039	85.24906	170.4649	118.4054	1	87.86569	6.457228	0.001448	0.003304	0.418714	Glycitin			2.704	446.1204	C22H22C	40246-10-4	2	Isoflavon	C16195	ko00943//	Biosynthe	Metabolism
NEG0031	271.6401	152.2313	303.6955	239.4091	169.5451	204.1909	940.0463	824.3724	653.3822	440.3789	754.8868	739.4142	223.452	725.4134	1.698838	4.42E-05	0.000218	1.070166	Astragali	Astragalir	94.7	2.722	448.0982	C21H20C	480-10-4	5	Flavonoic	C12249	ko01110//	Biosynthe	Metabolism
NEG0031	0.802549	0.508566	0.78891	0.373178	0.33948	0.527626	27.93214	26.30908	16.27727	21.93534	42.77297	34.95036	1	28.36286	4.825931	2.80E-05	0.000151	0.252191	Luteolin 4	luteolin 4'-O-glucosi		2.964	448.0988	C21H20C	6920-38-3	3	Flavonoic	-	-	-	-
NEG0031	11.45741	11.29223	14.82917	9.919907	8.910174	12.81308	11.34786	9.717135	8.766421	8.349353	10.05463	11.84561	11.537	10.0135	-0.20432	0.168517	0.203825	0.032476	Piperenol C			0.855	448.1406	C22H24C	174819-31-	8	Carboxyli	-	-	-	-
NEG0031	6.275315	5.573305	6.963753	6.014257	1.013423	4.684593	10.51743	8.893993	12.00805	14.49238	17.31355	25.08844	5.087441	14.71897	1.532665	0.003674	0.007179	0.134461	Lonchocarpenin			1.997	448.1928	C27H28C	NA	9	-	-	-	-	-
NEG0031	198.2732	121.3287	201.1332	129.4496	165.7135	130.9749	29.50328	33.1602	28.20198	27.22887	50.01154	38.16082	157.8122	34.37778	-2.19866	9.52E-06	7.56E-05	0.501477	Cajaisoflavone			2.66	450.172	C26H26C	72578-99-5	9	Isoflavon	-	-	-	-
NEG0031	168.9834	96.15582	153.0551	114.526	101.1645	149.3844	123.8811	72.65425	79.36249	127.4444	107.3648	213.4698	130.5449	120.6961	-0.11317	0.692018	0.720379	0.035994	Catechin 5-glucoside			0.821	452.1277	C21H24C	88126-53-8	9	Flavonoic	-	-	-	-
NEG0031	8.908123	4.749209	8.679073	4.540427	33.54939	7.147574	6.670591	6.461539	5.206137	2.867254	3.925646	4.4069	11.2623	4.923011	-1.19389	0.194846	0.229834	0.071063	Heteroart	(2R,3S,4S)-3-Hydrox		8.533	452.1847	C26H28C	170894-23-	3	Flavonoic	-	-	-	-
NEG0031	0.615565	0.490662	0.196111	0.320879	2.059607	0.462914	7.383383	3.406329	3.094496	2.336233	5.902147	2.212139	1	4.055788	2.019982	0.003971	0.007641	0.082659	LysoPE(0	Phosphatidylethano		7.344	453.2831	C21H44N	NA	5	Glyceropl	-	-	-	-
NEG0031	5.431977	6.779981	5.861497	3.982931	4.394373	8.243256	23.82502	24.27148	25.89326	20.40931	29.34957	25.24098	5.782336	24.8316	2.102453	6.43E-08	4.32E-06	0.207989	6''-O-Acetyldaidzin			1.362	458.1252	C23H22C	71385-83-6	8	Isoflavon	-	-	-	-
NEG0032	0.149098	0.134865	0.160295	0.115571	0.180838	0.151142	2.518783	2.428702	0.497427	0.965151	0.383616	1.084001	1	1.312947	0.392808	0.012484	0.02114	0.044455	Wistin			3.318	460.1351	C23H24C	19046-26-5	4	Isoflavon	-	-	-	-
NEG0032	116.1664	12.30293	7.301395	5.296183	5.21189	11.04507	6.034249	5.530094	5.209511	5.648104	9.323589	9.027301	26.22065	6.795475	-1.94806	0.306997	0.345032	0.11072	Aspidin	TU4153400		6.517	460.208	C25H32C	584-28-1	3	-	-	-	-	-
NEG0032	0.134495	0.267914	0.17776	0.103119	0.090581	0.117033	45.04449	49.12897	104.5792	60.95471	67.69566	80.92974	1	68.05546	6.088639	1.99E-05	0.000124	0.369577	Isoscopar	Isoscoparin		3.008	462.1147	C22H22C	20013-23-4	3	-	-	-	-	-
NEG0032	0.409757	4.63944	0.500575	0.384178	15.13839	3.923355	3.01132	0.808123	0.369616	0.35088	108.0087	0.518977	4.165949	18.8446	2.177434	0.433514	0.467102	0.112846	Adenylos	Adenylsuccinic acid		1.517	463.0723	C14H18N	19046-78-7	3	Purine nu	C03794	ko01100//	Amino ac	Metabolism
NEG0032	0.275869	0.230027	0.294555	0.230614	0.219296	0.241952	752.4037	344.269	238.7176	585.8467	419.3075	754.7545	1	515.8832	9.010901	0.000163	0.000554	1.0514	Quercetir	Quercetin	98.3	2.562	464.0937	C21H20C	482-35-9	3	Flavonoic	C05623	ko01100//	Biosynthe	Metabolism
NEG0032	4.995726	9.022332	7.926209	4.664047	4.061826	8.806929	27.16526	14.60566	23.68336	17.25873	17.12989	22.20811	6.579511	20.34183	1.628397	7.94E-05	0.00034	0.171307	Hesperetin 3'-O-glucuronide			1.163	476.1352	C23H24C	NA	7	Flavonoic	-	-	-	-
NEG0032	38.33897	29.57616	44.47688	36.92104	32.04098	31.27104	12.08144	7.687458	7.313548	10.94827	14.83266	12.09245	35.43751	10.82597	-1.71078	2.30E-06	3.14E-05	0.226556	Kanokoside A			3.031	476.1874	C21H32C	64703-85-1	4	Organoox	-	-	-	-
NEG0032	362.1702	91.0456	394.6003	73.43828	42.1811	121.0351	4552.905	2384.092	3455.789	4251.133	3150.327	5692.871	180.7451	3914.52	4.436807	1.53E-05	0.000104	2.844316	Isorhamn	Isorhamnetin-3-gluc		2.769	478.1087	C22H22C	NA	5	-	-	-	-	-
NEG0032	0.136778	0.124368	0.161139	0.116981	1.001833	0.141969	0.153127	0.352179	0.343345	0.133715	0.274461	0.194745	1	1	0	0.801632	0.825464	0.002176	LysoPE(1	Phosphatidylethano		8.604	481.3146	C23H48N	69747-55-3	4	Glyceropl	-	-	-	-
NEG0032	25.81708	30.2251	35.97944	20.09683	6.20629	94.81082	1.675371	1.51369	1.386657	1.287182	1.700075	2.166966	35.52259	1.621657	-4.4532	0.022378	0.0348	0.236159	Karakin			7.228	483.0936	C15H21N	1400-11-9	7	-	-	-	-	-
NEG0033	8.077926	4.409636	8.531691	6.347821	5.859562	5.746832	2.626088	4.999643	4.556261	2.710809	4.358099	6.570899	6.495578	4.303633	-0.5939	0.031355	0.046056	0.053274	Cyanidin-3-galactoside			2.702	484.075	C21H21C	NA	4	-	-	-	-	-
NEG0033	21.33663	10.37609	33.44334	27.43826	4.537917	11.36868	43.04374	43.85829	26.06902	39.38505	53.03284	40.15567	18.08349	40.9241	1.178278	0.00275	0.005789	0.206149	CDP-choline			2.074	488.1147	C14H26N	987-78-0	15	Pyrimidir	C00307	ko01100//	Global an	Metabolism
NEG0033	0.80959	0.827085	1.341773	0.68732	0.584622	3.803356	62.82876	62.93635	57.51403	61.86586	108.2052	61.60131	1.342291	69.15859	5.687139	6.09E-06	5.52E-05	0.386392	6''-O-Ace	[Similar to: NP-0219		3.31	488.13	C24H24C	134859-96-	3	Isoflavon	-	-	-	-
NEG0033	0.145874	0.145807	0.166358	0.128342	0.137423	0.168856	0.197818	0.175846	0.159372	0.144175	0.187351	0.227575	1	1	0	0.035411	0.05053	0.007817	LysoPC(16:0)			9.301	495.3305	C24H50N	17364-16-8	4	Glyceropl	C04230	ko00564//	Lipid met	Metabolism
NEG0033	11.52169	14.98088	19.8692	13.06296	12.28095	13.77068	19.36514	16.61825	17.15584	15.57923	25.37606	22.23261	14.24773	19.38786	0.444421	0.025952	0.039608	0.096536	Withaperuvin G			5.337	502.2632	C28H38C	NA	13	Steroids a	-	-	-	-
NEG0033	1.377126	2.353359	1.995157	1.681703	2.874028	1.758285	8.86416	7.993455	7.898371	8.990846	12.28042	12.41622	2.00661	9.740579	2.279248	4.73E-06	4.80E-05	0.132756	Mycalam	Mycalamide A		7.803	503.2708	C24H41N	115185-92-	4	-	-	-	-	-
NEG0033	7.277364	14.03947	10.90415	13.96896	8.546945	12.96641	153.1536	75.20314	49.59176	103.3388	146.495	163.4372	11.28388	115.2033	3.351846	0.000274	0.000834	0.482105	Raffinose			0.801	504.1666	C18H32C	512-69-6	4	Organoox	C00492	ko02010//	Carbohyc	Environmental Information Processing;Metabolism
NEG0033	0.335828	0.30626	0.383889	0.2799	0.282432	0.988041	2396.519	2521.542	2143.261	2246.93	3531.84	2688.465	1	2588.093	11.33767	1.78E-07	5.87E-06	2.431701	Pendulin	streptonigrin		2.734	506.1417	C24H26C	NA	1	-	-	-	-	-
NEG0033	0.148844	0.132443	0.17289	0.138303	0.103113	0.166563	145.2953	83.79757	43.74029	156.0398	150.1873	90.41825	1	111.5798	6.801932	0.000136	0.000511	0.475533	Malonylgenistin			2.928	518.104	C24H22C	NA	3	-	C16192	ko00943//	Biosynthe	Metabolism
NEG0033	3.096704	2.752346	1.446562	0.329246	1.756057	1.400292	27.9803	82.88427	15.60584	37.78531	20.2691	70.08262	1.796868	42.43457	4.561684	0.004941	0.009139	0.265397	(6-{[3,4-di	[6-[3,4-dihydroxy-2,		2.037	518.1615	C22H30C	NA	4	Cinnamic	-	-	-	-
NEG0034	0.161284	0.091597	0.129721	0.102945	0.079915	0.123767	48.7817	75.70255	39.6855	40.03411	69.97315	55.49216	1	54.94486	5.779913	4.79E-06	4.80E-05	0.349885	Isolaricire	Lariciresir	93.8	2.522	522.2082	C26H34C	145748-07-	3	Lignan gl	-	-	-	-
NEG0034	16.69074	14.31349	21.20825	16.00909	13.77154	20.43638	21.84513	17.98295	22.06138	14.25439	23.94019	12.3535	17.07158	18.73959	0.134493	0.483191	0.515675	0.029762	Ganosporeric acid A			3.196	526.2522	C30H38C	135357-25-	8	Prenol lip	-	-	-	-
NEG0034	650.39	610.7758	718.6949	679.3674	653.9767	642.7871	856.8065	701.4467	815.8688	503.3711	793.7013	864.8233	659.332	756.0029	0.197387	0.125786	0.157646	0.396469	Proscillar	Proscillaridin		9.491	530.2856	C30H42C	NA	4	-	-	-	-	-

NEG0034	0.163195	0.428444	0.295338	0.136097	0.352033	1.150137	32.91753	35.85977	26.70519	18.8892	13.57472	16.64095	1	24.09789	4.590835	8.35E-05	0.000354	0.217209	6'-Malonyltrifolirhizin			3.234	532.1195	C25H24O	135574-57-	4	Isoflavon	-	-	-	-
NEG0034	4.400021	3.167869	0.137055	3.747939	6.534693	3.57714	28.94016	29.70992	20.42596	17.94527	40.97695	28.31156	3.594119	27.7183	2.947129	3.52E-05	0.000179	0.235019	Dalpanin	Dalpanin		2.598	534.1717	C26H30O	-	3	-	-	-	-	-
NEG0034	0.479492	0.836808	0.959679	0.24355	0.441173	0.169236	56.23567	51.209	46.0704	34.4131	53.67212	40.71557	1	47.05264	5.556204	8.41E-08	4.58E-06	0.327413	Vaccinoside			2.866	536.1508	C25H28O	36138-58-6	4	Prenol lip	-	-	-	-
NEG0034	3.120671	3.331759	4.187274	3.451979	2.495699	3.943415	49.67456	65.70418	12.69258	53.26899	29.15296	90.54586	3.4218	50.17319	3.874089	0.001849	0.00412	0.299605	Sibiricose	Sibiricose	90.9	2.013	548.1722	C23H32O	NA	3	-	-	-	-	-
NEG0034	0.403333	0.291416	0.416205	0.304613	0.251561	0.358263	1.465601	1.412324	0.315478	0.283552	1.104131	0.381817	1	1	0	0.060291	0.080883	0.027953	Quercetin 3-O-(6-O-malonyl-β			1.426	550.104	C24H22O	96862-01-0	14	Flavonoic	C12638	ko00944//	Biosynthe	Metabolism
NEG0034	10.43199	7.603359	11.0384	8.000289	7.235705	8.792542	67.5975	42.44596	22.21572	25.1844	39.94965	42.81437	8.85038	40.0346	2.177436	0.000848	0.002085	0.262389	Neoliquiritin 2''-apioside			1.198	550.172	C26H30O	135432-48-	6	Flavonoic	-	-	-	-
NEG0034	4.690383	4.074762	3.078452	3.898936	5.984657	3.963592	61.46656	10.27706	9.549901	52.19594	51.85374	65.95889	4.281797	41.88368	3.2901	0.004609	0.008565	0.270441	Physalin I			7.674	558.2106	C29H34O	NA	1	Steroids a	-	-	-	-
NEG0035	0.292524	0.26677	0.304083	0.428786	0.246014	0.998576	2223.176	2386.373	1962.3	2163.582	3493.244	2666.007	1	2482.447	11.27755	6.14E-07	1.30E-05	2.376756	Malvidin 3-glucoside-pyruvat			2.739	560.114	C26H24O	NA	4	Flavonoic	-	-	-	-
NEG0035	0.117014	0.277729	0.164705	0.105589	0.615197	0.667657	11.92935	16.80861	13.00154	10.79547	18.0782	15.36277	1	14.32932	3.840898	3.18E-07	8.65E-06	0.178094	Citrusin I	Lariciresi	90.1	2.408	568.2132	C27H36O	105279-10-	4	Lignan gl	-	-	-	-
NEG0035	0.085057	0.140039	0.80668	0.795063	0.084386	1.220051	0.847938	0.198966	0.753862	1.187766	0.870898	0.973584	1	1	0	0.263888	0.301021	0.012095	Ganoderic acid H			14.479	572.294	C32H44O	98665-19-1	7	Prenol lip	-	-	-	-
NEG0035	0.19302	0.198653	0.237884	0.171655	0.140591	0.207318	55.42929	27.18842	17.20124	7.956307	14.60264	8.148422	1	21.75439	4.443234	0.01472	0.023969	0.195929	Kaempferol 3-xylosylglucosid			2.647	580.141	C26H28O	29322-07-4	3	Flavonoic	-	-	-	-
NEG0035	225.1115	183.2071	219.4051	146.0445	179.3496	161.0681	261.1096	150.4998	213.692	120.2617	178.9797	202.9535	185.6976	187.916	0.017133	0.928054	0.932952	0.061144	Caracurir	N-[(1S,3aS	63	11.571	584.3206	C38H40N	630-87-5	9	-	-	-	-	-
NEG0035	0.124166	0.127268	0.159142	0.116411	0.290843	0.135784	1.982897	5.777467	5.786113	0.524701	1.604819	1.474557	1	2.858426	1.515221	0.017091	0.027361	0.06162	3,5-Digalloylepicatechin			3.075	594.1022	C29H22O	37484-74-5	2	Flavonoic	-	-	-	-
NEG0035	2.762299	0.885315	1.331371	1.58828	1.015365	2.19114	3.087126	1.664913	0.950882	3.817814	6.132717	1.705571	1.628962	2.893171	0.828699	0.15899	0.193532	0.036217	Daphnandrine			8.355	594.2781	C36H38N	1183-76-2	8	-	-	-	-	-
NEG0035	0.13343	0.109807	0.150771	0.109467	0.103635	0.136079	16.37647	17.25982	21.12332	18.43516	28.554	16.72035	1	19.74485	4.303405	1.16E-06	2.21E-05	0.206179	Pelargonidin 3-O-3'',6''-O-dim			2.61	604.1076	C27H24O	NA	1	-	C16288	ko00942//	Biosynthe	Metabolism
NEG0035	30.56875	21.28773	27.01146	18.74817	31.25196	32.36236	91.23014	64.98158	61.04047	50.22132	86.70626	76.42707	26.87174	71.76781	1.417247	6.47E-05	0.00029	0.322609	Phaeophorbide b			9.778	606.2454	C35H34N	20239-99-0	4	Tetrapyrr	-	-	-	-
NEG0035	29.70303	30.4207	35.70956	31.56328	34.1699	39.27045	107.6115	113.229	106.6349	84.26371	104.3249	95.97801	33.47282	102.007	1.607606	2.88E-08	3.66E-06	0.392454	Cepharanthine			12.046	606.2818	C37H38N	481-49-2	14	-	-	-	-	-
NEG0036	287.9127	151.8138	398.0551	288.8345	134.9152	175.1073	502.9774	799.0332	392.1792	387.8505	791.7584	578.3664	239.4398	575.3608	1.264804	0.003001	0.006128	0.805862	Rutin	cyanidin-3,5-di-O-gl		2.03	610.151	C27H30O	153-18-4	3	Flavonoic	C05625	ko01100//	Biosynthe	Metabolism
NEG0036	19.30681	12.23949	10.8232	8.889374	15.254	12.49614	58.90357	53.87693	44.29544	37.51312	59.10238	46.79004	13.16817	50.08025	1.927187	2.30E-06	3.14E-05	0.293178	Glycerol 1	2-(Decanoyloxy)-1,3		10.36	610.5146	C37H70O	60138-22-9	4	Glyceroli	-	-	-	-
NEG0036	21.88419	15.59366	17.2954	13.91408	19.24532	56.60525	605.7089	517.2908	518.0491	167.5954	313.6314	363.9538	24.08965	414.3716	4.10444	0.000158	0.000554	0.910181	Cyanidin 3-glucogalactoside			2.382	611.1539	C27H31O	142562-01-	11	Flavonoic	-	-	-	-
NEG0036	9.312085	9.622255	5.08103	4.764142	6.086945	10.39812	69.33364	98.70178	102.5825	84.86272	80.65096	112.4705	7.544096	91.43368	3.599306	1.65E-07	5.87E-06	0.425383	Glutathio	Glutathione (oxidize		0.858	612.1493	C20H32N	27025-41-8	4	Carboxyli	C00127	ko00480//	Metabolis	Metabolism
NEG0036	0.303325	0.231427	0.332876	0.245388	0.213405	0.272904	23.39106	18.31862	5.935083	5.283842	4.04406	2.771643	1	9.957385	3.315767	0.020799	0.032483	0.125169	Camellianin A			2.816	620.1639	C29H32O	109232-77-	16	Flavonoic	-	-	-	-
NEG0036	0.343433	0.136363	0.178319	0.151258	0.112004	0.164837	96.75739	134.1824	58.59231	192.4953	130.9365	205.0757	1	136.34	7.091065	0.000135	0.000511	0.517828	Quercetin	NCGC00385506-01!5		1.93	626.1459	C27H30O	42900-82-3	3	Flavonoic	-	-	-	-
NEG0036	16.71097	27.60466	35.5006	29.34305	7.958679	25.24074	53.52255	65.15662	40.91978	69.05823	94.34347	83.22408	23.72645	67.70412	1.512747	0.000574	0.00154	0.297272	Kuwanon L			0.795	626.1879	C35H30O	88524-65-6	14	Flavonoic	-	-	-	-
NEG0036	1.531638	1.51574	1.477127	1.185531	1.367159	1.821696	15.35883	11.40141	6.789527	5.212174	12.6601	12.72452	1.483149	10.69109	2.849674	0.000174	0.000588	0.146595	Tricin 7-neohesperidoside			1.174	638.1878	C29H34O	53766-40-8	5	Flavonoic	-	-	-	-
NEG0036	1.895651	2.184388	2.119019	1.878346	0.973724	3.085174	84.87599	19.1062	67.99343	36.6279	16.90308	41.19731	2.022717	44.45065	4.457838	0.003269	0.00659	0.275749	Isorhamn	3-[(2S,3R,4S,5S,6R)-4		2.292	640.1617	C28H32O	171828-59-	3	Flavonoic	-	-	-	-
NEG0036	0.198133	0.385893	0.543864	0.479622	0.252939	0.547947	20.49164	4.930274	17.82559	9.768445	4.146809	11.62764	1	11.46507	3.519173	0.002235	0.00481	0.141688	Petunin			2.288	641.1653	C28H33O	25846-73-5	10	Flavonoic	-	-	-	-
NEG0037	0.122224	0.111463	0.127054	0.101869	0.102791	0.123783	1251.179	1427.692	694.1402	824.0435	1775.811	1183.535	1	1192.733	10.22006	2.37E-05	0.000141	1.645796	Malonylapiin			2.737	650.1457	C29H30O	NA	3	-	C05622	ko00944//	Biosynthe	Metabolism
NEG0037	6.12497	4.420401	5.673081	4.487937	4.382531	5.176559	44.30284	41.2676	35.14261	41.56406	59.28105	50.56134	5.044247	45.35325	3.168495	3.91E-07	9.32E-06	0.304254	Hespereti	[Similar to: NP-0219		2.541	650.1826	C30H34O	NA	3	Flavonoic	-	-	-	-
NEG0037	2.820063	2.203684	3.201273	2.083225	2.628748	8.788466	89.8211	51.64344	52.46308	23.0843	46.23331	44.8228	3.62091	51.34467	3.82579	0.000321	0.000941	0.322088	Patuletin	NCGC00180501-02!3		2.373	656.1561	C28H32O	NA	4	-	-	-	-	-
NEG0037	0.127059	0.09839	0.141545	0.1124	0.083375	0.116272	0.633415	0.695242	2.4388	0.25815	0.426833	1.183549	1	1	0	0.02969	0.044015	0.033261	Isorhamnetin 3-(4''-sulfatoruti			2.229	704.1239	C28H32O	NA	2	-	-	-	-	-
NEG0037	0.152531	0.116376	0.167391	0.123142	0.25616	0.135446	3.553065	66.69498	0.847512	25.50649	3.987431	43.87945	1	24.07816	4.589653	0.053228	0.073212	0.171102	Natsudai	NCGC00385663-01_		2.828	724.2187	C33H40O	149402-86-	3	Flavonoic	-	-	-	-
NEG0037	4.052226	1.831243	1.729927	0.478386	2.114111	2.130228	117.3591	69.03661	55.22934	76.44971	173.8952	149.2077	2.05602	106.8629	5.699763	0.000314	0.000928	0.485485	2''-(6''-p-C	NCGC00385052-01![		2.613	756.1876	C36H36O	113447-39-	3	Flavonoic	-	-	-	-
NEG0037	0.403442	0.304765	0.472895	0.331101	0.278475	0.367043	811.8812	384.1669	289.6523	493.9582	961.3714	824.4243	1	627.5757	9.293646	0.000226	0.000722	1.191407	Cyanidin 3-glucoside 5-caffeo			2.444	772.1826	C36H36O	-	3	-	C16372	ko00942//	Biosynthe	Metabolism
NEG0037	250.9682	184.0052	309.0088	228.6171	189.9588	497.5976	965.2487	1670.609	1179.516	590.2052	1216.922	873.9253	276.6926	1082.738	1.968328	0.000447	0.001261	1.249883	Quercetin	NCGC00380680-01!5		1.806	772.2037	C33H40O	50867-29-3	3	Flavonoic	-	-	-	-
NEG0037	46.41961	34.72675	58.93067	45.43214	43.13305	98.97246	213.5404	394.9123	251.9869	125.2655	350.2782	204.6455	54.60245	256.7715	2.233447	0.000684	0.001761	0.630912	Cyanidin 3-sophoroside 5-gluc			1.805	773.2071	C33H41O	47888-56-2	8	Flavonoic	-	-	-	-
NEG0037	0.130494	0.099563	0.143208	0.105569	0.09181	0.117407	29.31668	13.76947	11.63837	9.512912	18.83214	17.95194	1	16.83692	4.073556	0.000178	0.000588	0.195754	Kaempferol 3-sophoroside 7-g			2.849	786.1981	C33H38O	140163-56-	3	-	-	-	-	-
NEG0038	0.256681	0.24889	0.289477	0.202716	0.220068	0.454531	118.9937	156.9963	115.8925	134.519	165.8121	187.3126	1	146.5877	7.19562	1.72E-07	5.87E-06	0.574802	Moracetin			1.75	788.1987	C33H40O	61474-16-6	3	Flavonoic	-	-	-	-
NEG0038	0.211118	0.171471	0.294048	0.336284	0.157195	0.212938	16.92722	5.631582	6.12028	16.8538	26.77157	24.81509	1	16.18659	4.016727	0.001403	0.003239	0.181617	Quercetin 7-glucuronoside 3-s			2.37	802.1933	C33H38O	140163-57-	16	Flavonoic	-	-	-	-
POS00001	145.1738	104.344	118.2136	100.8358	68.48734	101.9002	170.0823	147.4677	137.1753	173.3814	197.5916	147.6875	106.4925	162.231	0.607298	0.002254	0.005943	0.356375	Pyrrolidi	Pyrrolidine		0.858	71.07385	C4H9N	123-75-1	5	Pyrrolidi	-	-	-	-
POS00002	233.733	134.2793	159.113	127.0117	117.8286	156.2297	284.5621	297.3048	268.1604	218.7871	296.7117	214.4293	154.6992	263.3259	0.767383	0.000824	0.002694	0.487533	Piperidin	Piperidine		1.306	85.08942	C5H11N	110-89-4	2	Piperidin	C01746	ko00960//	Biosynthe	Metabolism
POS00003	363.3579	275.9185	235.4447	217.9109	162.2493	276.5393	521.9798	539.2644	396.249	478.1616	570.4205	517.0249	255.2368	503.8501	0.981158	5.43E-05	0.000395	0.795907	Crotonic a	Crotonic acid		0.776	86.03702	C4H6O2	3724-65-0	2	Fatty Acy	-	-	-	-
POS00004	7.139554	9.538753	5.108107	4.048732	5.277684	8.736945	64.78265	62.00543	59.95218	62.20147	84.28672	59.73513	6.641629	65.49393	3.301752	3.60E-08	6.30E-06	0.405025	Putrescin	PUTRESCINE		0.643	88.10027	C4H12N2	110-60-1	2	Organoni	C00134	ko01100//	Amino ac	Environmental Information Processing;Metabolism
POS00005	435.4542	128.7322	135.0755	501.307	91.08293	142.4051	583.7659	181.3804	202.4835	317.0859	367.5908	219.1215	239.0095	311.9047	0.384037	0.464979	0.529785	0.239236	beta-Alan	beta-Alanine		0.885	89.04787	C3H7NO	107-95-9	2	Carboxyli	C00099	ko01100//	Carbohyd	Metabolism
POS00006	1160.578	827.5365	807.3696	744.6803	560.7967	802.3799	737.611	699.7752	551.6212	847.153	1038.012	750.2647	817.2235	770.7396	-0.08449	0.662827	0.710637	0.101669	L-Alanine	DL-Alanine		0.787	89.04788	C3H7NO	56-41-7	2	Carboxyli	C01401	ko00460//	Metabolis	Metabolism
POS00007	14.06819	3.301038	4.422197	2.61832	9.196656	4.729423	9.470713	5.699849	1.869516	9.802725	18.42117	6.864455	6.389304	8.688072	0.443377	0.446595	0.512409	0.048616	N-Methyl	N-Methyl-2-pyrrolic		1.754	99.06853	C5H9NO	872-50-4	0	Pyrrolidi	-	-	-	-
POS00008	2.550633	3.527651	2.11422	1.8098	1.332588	3.976155	1.665044	2.54568	1.529385	1.995199	8.677424	1.764302	2.551841	3.029506	0.247544	0.701953	0.742844	0.024002	Cadaveri	CADAVERINE		0.646	102.1157	C5H14N2	462-94-2	0	Organoni	C01672	ko01100//	Amino ac	Metabolism
POS00009	1549.797	999.106	1557.977	780.8814	812.465	1134.856	5571.293	2679.875	2283.512	2030.517	3701.543	2745.427	1139.18	3168.695	1.475892	0.004286	0.009825	2.125927	gamma-A	ϒ-Aminobutyric aci		0.931	103.0635	C4H9NO	20791	1	Carboxyli	C00334	ko01100//	Amino ac	Metabolism
POS00010	21827.64	12050.06	11927.28	10714.71	12516.61	11079.11	8359.076	8669.19	19070.38	6150.049	12336.64	10331.09	13352.57	10819.4	-0.3035	0.339659	0.411365	1.897137	Choline	Choline		0.919	103.0997	C5H13NO	62-49-7	0	Organoni	C00114	ko01100//	Amino ac	Environmental Information Processing;Metabolism
POS00011	46.8745	61.23353	30.82178	29.00797	123.8594	78.87114	39.85114	49.01648	31.63981	36.61337	28.53266	32.04623	61.77805	36.28328	-0.76779	0.118224	0.166635	0.1806	Styrene	Styrene		1.87	104.0625	C8H8	100-42-5	0	Benzene a	C07083	ko01100//	Global an	Metabolism
POS00012	988.1285	926.3711	872.7323	845.4371	481.063	860.4612	353.6336	360.1315	255.3416	308.0991	395.5154	363.9899	829.0322	339.4518	-1.28822	7.11E-05	0.000456	1.090229	L-Serine	D-Serine		0.791	105.0426	C3H7NO	56-45-1	0	Carboxyli	C00065	ko01100//	Amino ac	Environmental Information Processing;Genetic Information Processing;Human Diseases;Metabolism
POS00013	32.10019	14.35159	19.18352	16.82882	13.6368	22.32171	87.34065	43.2396	28.43869	71.99437	87.20422	32.19984	19.7371	58.4029	1.56513	0.006874	0.014686	0.299847	Benzaldel	Benzaldehyde		1.678	106.0418	C7H6O	100-52-7	0	Benzene a	C00261	ko01120//	Global an	Metabolism
POS00014	3.095086	1.638459	1.380766	1.262093	7.512357	0.407009	0.408543	0.504508	0.385811	0.338288	0.488027	0.393397	2.549295	1	-1.3501	0.07116	0.107232	0.057174	S-Methyl	S-Methyl methanesu		1.785	109.9859	C2H6OS2	13882-12-7	0	Thiosulfir	-	-	-	-
POS00015	1403.737	1616.212	910.7151	1145.579	1083.779	1302.307	1768.104	1639.548	1089.99	1240.106	1907.91	1166.774	1243.722	1468.739	0.239914	0.226005	0.293269	0.574658	alpha-Chl	.alpha.-Chlorohydri		14.504	110.0129	C3H7ClO	96-24-2	5	Halohydr	-	-	-	-
POS00016	258.9884	169.4996	199.257	188.0977	105.7895	157.5471	209.2969	280.2181	219.1241	205.6956	250.4513	237.918	179.8632	233.784	0.378276	0.04617	0.074008	0.282001	Cytosine	Cytosine		1.232	111.0433	C4H5N3O	71-30-7	0	Diazines	C00380	ko01100//	Global an	Metabolism
POS00017	275.4038	295.724	196.8294	179.5702	178.0346	273.4261	247.9273	356.6238	327.7836	241.0012	283.5646	249.5714	233.1647	284.412	0.286633	0.112808	0.160383	0.23326	Uracil	Uracil		1.231	112.0272	C4H4N2O	66-22-8	0	Diazines	C00106	ko01100//	Global an	Metabolism
POS00018	79.98079	41.02436	36.43294	49.95618	13.90677	18.99486	422.373	120.3059	275.5025	19.39315	438.7883	46.90971	40.04932	220.5454	2.461226	0.039676	0.064933	0.578298	Maleamic	acid		0.828	115.0291	C4H5NO	557-24-4	18	-	C01596	ko01120//	Global an	Metabolism
POS00019	6738.707	5760.02	3573.722	3998.734	671.0822	6431.334	11025.52	22777.77	13455.26	15055.13	25075.59	22203.5	4528.933	18265.46	2.011875	0.000302	0.001313	5.886312	L-Proline	D-(+)-Proline		0.93	115.0631	C5H9NO	147-85-3	1	Carboxyli	C00148	ko01100//	Amino ac	Environmental Information Processing;Genetic Information Processing;Metabolism
POS00020	51.66087	29.89153	33.48816	28.32104	29.50911	32.2423	58.3946	55.99239	53.02192	53.46459	60.22635	50.59215	34.1855	55.282	0.693425	0.000281	0.001264	0.218176	Indole	Indole		1.939	117.0575	C8H7N	120-72-9	2	Heteroarc	C00463	ko01100//	Amino ac	Metabolism
POS00021	108.433	52.83176	85.20629	65.62262	74.05453	50.9659	108.1497	56.34651	97.32972	93.05945	102.1866	91.30361	72.85236	91.39592	0.327154	0.140067	0.192446	0.139235	Betaine	Betaine		14.479	117.0787	C5H11NO	590-46-5	2	Carboxyli	C00719	ko01100//	Amino ac	Environmental Information Processing;Metabolism
POS00022	838.6441	604.2006	659.3165	507.9537	400.4352	649.6176	877.8378	597.962	1134.445	1124.523	665.0435	615.0421	610.0279	835.8089	0.454298	0.085273	0.12393	0.466456	L-Valine	L-Valine		1.201	117.0787	C5H11NO	72-18-4	2	Carboxyli	C00183	ko01100//	Amino ac	Environmental Information Processing;Genetic Information Processing;Metabolism
POS00023	1139.013	1031.048	844.7491	685.1299	409.0195	850.9022	1215.225	730.0504	628.7032	824.6192	1062.428	917.0147	826.6437	896.34	0.11678	0.623724	0.678258	0.198462	L-Homos	DL-Homoserine		0.792	119.0579	C4H9NO	672-15-1	2	Carboxyli	C00263	ko01100//	Amino ac	Metabolism
POS00024	24.93186	41.69095	30.0953	22.38817	14.64755	60.07128	8.466298	9.298243	12.51778	10.38856	7.394202	7.335744	32.30418	9.23347	-1.80678	0.006315	0.013767	0.214227	L-Threon	L-(-)-Threonine		0.794	119.0588	C4H9NO	72-19-5	4	Carboxyli	C00188	ko01100//	Amino ac	Environmental Information Processing;Genetic Information Processing;Metabolism
POS00025	33.13272	31.49851	17.23568	13.6731	60.15554	40.89634	19.90095	23.83731	16.49506	25.33909	15.41888	15.90468	32.76531	19.48266	-0.74998	0.091179	0.131927	0.139029	Phenyleth	Phenylethylamine		1.87	121.0889	C8H11N	64-04-0	2	Benzene a	C05332	ko00360//	Amino ac	Metabolism
POS00026	200.6377	118.0911	140.9759	136.1393	36.52562	113.0717	124.4589	145.2997	131.9906	99.02915	96.54653	115.6509	124.2402	118.8293	-0.06424	0.819029	0.855663	0.065337	Niacinam	Nicotinamide		0.958	122.0477	C6H6N2O	98-92-0	1	Pyridines	C00153	ko01100//	Global an	Metabolism
POS00027	97.64667	63.59053	113.3972	64.50886	74.13566	62.11244	61.02941	83.90077	118.8351	14.27519	59.19388	10.08163	79.2319	57.886	-0.45287	0.288474	0.358635	0.165676	Nicotinic	Nicotinic acid		0.897	123.0317	C6H5NO	59-67-6	2	Carboxyli	C00253	ko01100//	Biosynthe	Metabolism
POS00028	156.4759	86.60905	271.2679	68.5365	124.0603	20.25662	344.3631	306.1335	235.0385	510.1855	624.4705	311.0855	121.201	388.5461	1.680684	0.003356	0.00813	0.800391	Maltol	Maltol		1.072	126.0313	C6H6O3	118-71-8	3	Pyrans	C11918	ko01130//	Global an	Metabolism
POS00029	1046.835	534.5536	660.7068	634.5426	376.9116	733.5882	1109.558	981.1277	770.7558	859.4421	1151.739	1018.675	664.523	981.883	0.563232	0.015643	0.029568	0.779238	Hydroxyl	4-Hydroxy-6-Methy		0.826	126.0313	C6H6O3	533-73-3	3	Benzene a	C02814	ko01100//	Global an	Metabolism
POS00030	1896.628	1397.973	1257.024	1084.942	1546.607	1510.1	457.8302	850.4444	543.5054	396.4168	986.4989	391.4951	1448.879	604.3651	-1.26144	0.00026	0.001217	1.406535	Coniine	(+/-)-Coniine		0.673	127.1356	C8H17N	458-88-8	3	-	-	-	-	-
POS00031	8398.571	2174.676	2635.101	3282.44	9327.47	1733.469	8907.26	4751.293	4951.812	9496.539	6399.473	4486.708	4591.955	6498.847	0.501076	0.27215	0.340969	1.259449	Pyroglut	L-Pyroglutamic acid		0.884	129.0421	C5H7NO	98-79-3	3	Carboxyli	C01879	ko01100//	Global an	Metabolism
POS00032	2814.674	2120.213	1629.628	1174.382	3999.154	1796.948	1623.935	1506.89	460.3262	708.592	904.7175	1026.413	2255.833	1038.479	-1.11919	0.022883	0.04134	1.483775	DL-pipec	D-(+)-Pipecolinic aci		1.218	129.0786	C6H11NO	535-75-1	2	Carboxyli	C00408	ko01100//	Amino ac	Metabolism
POS00033	224.7424	159.5279	112.426	164.7929	112.0131	112.466	208.5191	103.4666	157.7237	101.6976	128.4158	68.38878	147.6614	128.0352	-0.20575	0.488489	0.548921	0.130121	4-Hydroxyproline			0.789	131.0575	C5H9NO	51-35-4	4	Carboxyli	C01157	ko01100//	Amino ac	Metabolism
POS00034	5146.255	3601.655	3420.232	3128.623	2430.975	2582.109	6253.416	6046.788	5484.92	4990.267	6382.259	4184.607	3384.975	5557.043	0.715172	0.002117	0.005769	2.140366	L-Isoleuci	L-Isoleucine		1.306	131.0942	C6H13NO	73-32-5	3	Carboxyli	C00407	ko01100//	Amino ac	Environmental Information Processing;Genetic Information Processing;Metabolism
POS00035	11792.96	7086.06	7464.242	6874.216	6147.059	10296.85	14727.8	12925.15	12860.43	12789.61	16422.31	12538.5	8276.898	13710.63	0.728133	0.000628	0.002219	3.564983	L-Norleu	L-Norleucine		1.395	131.0942	C6H13NO	327-57-1	3	Carboxyli	-	-	-	-
POS00036	1122.844	909.8363	878.2273	836.4528	580.6842	837.2831	217.8279	261.1689	170.827	221.2402	283.1134	203.9315	860.8879	226.3515	-1.92726	5.53E-06	8.22E-05	1.284531	Glutaric a	Glutaric acid		0.841	132.042	C5H8O4	110-94-1	1	Carboxyli	C00489	ko01120//	Amino ac	Metabolism
POS00037	368.906	293.1704	251.8846	281.2584	198.6981	285.3385	167.6017	173.2224	108.6969	129.5928	198.2916	156.33	279.876	155.6226	-0.84674	0.000799	0.002638	0.523171	L-Aspara	Asparagine		0.791	132.053	C4H8N2O	70-47-3	3	Carboxyli	C00152	ko01100//	Amino ac	Genetic Information Processing;Metabolism
POS00038	27.36941	16.8223	20.40166	20.53898	7.534381	15.0325	7.406184	5.859935	7.543955	13.47211	7.86844	8.407716	17.94987	8.42639	-1.09099	0.008378	0.016911	0.142357	Tetralin	Tetralin		2.307	132.0934	C10H12	119-64-2	3	-	C14114	ko01120//	Global an	Metabolism
POS00039	839.3884	743.3881	613.8852	541.1215	232.0681	592.4892	632.1834	646.48	723.1177	748.2749	781.9416	617.2655	593.7234	691.5439	0.22003	0.299916	0.368694	0.266942	L-Asparti	L-Aspartic acid		0.796	133.037	C4H7NO	56-84-8	3	Carboxyli	C00049	ko01100//	Amino ac	Environmental Information Processing;Genetic Information Processing;Metabolism
POS00040	133.3195	106.6667	62.01097	72.79175	53.69753	87.18383	17.28559	13.33625	15.22804	12.2329	8.908391	8.586859	85.94506	12.59634	-2.77041	0.000137	0.000767	0.434882	p-Cymen	CY9070000		2.458	134.1092	C10H14	99-87-6	2	Prenol lip	C06575	ko01120//	Global an	Metabolism
POS00041	1291.461	947.7881	1114.521	1032.137	404.6226	1004.43	2395.887	1880.687	2031.287	1588.362	1896.905	1538.058	965.8266	1888.531	0.967429	0.000394	0.001612	1.441949	Adenine	Adenine		1.223	135.0539	C5H5N5	73-24-5	3	Imidazop	C00147	ko01100//	Global an	Metabolism
POS00042	175.6432	167.696	111.638	138.9455	108.2132	135.247	98.14806	92.78913	78.68145	58.07243	62.34446	68.55951	139.5638	76.43251	-0.86867	0.000737	0.002486	0.392412	Phenylacetic acid			0.843	136.0513	C8H8O2	103-82-2	8	Benzene a	C07086	ko01100//	Amino ac	Metabolism
POS00043	77.28531	55.28819	50.28473	61.97673	43.02325	46.4571	40.07253	31.58551	27.95605	21.94583	27.54232	16.62334	55.71922	27.62093	-1.01241	0.000936	0.002889	0.257612	Trigonelli	Trigonelline		0.814	137.0471	C7H7NO	535-83-1	4	-	C01004	ko00760//	Metabolis	Metabolism
POS00044	131.9201	137.303	102.3635	76.09547	80.44082	115.506	9.938101	6.880673	2.667427	4.836237	12.80955	2.483484	107.2715	6.602579	-4.02209	2.57E-06	4.95E-05	0.514543	Salicylic a	Salicylic acid		2.033	138.0313	C7H6O3	69-72-7	2	Benzene a	C00805	ko01100//	Amino ac	Environmental Information Processing;Metabolism
POS00045	5.0469	6.965149	3.80461	3.380542	5.642021	4.82708	2.05578	8.632514	2.058349	4.823931	3.061571	7.559827	4.944384	4.698662	-0.07354	0.850858	0.880477	0.00728	Urocanic	Urocanic acid		0.859	138.0423	C6H6N2O	104-98-3	4	Azoles	C00785	ko01100//	Amino ac	Metabolism
POS00046	73.19699	48.70138	51.29917	51.47791	30.46453	49.05882	45.31194	27.19781	37.36345	31.00657	42.21998	31.90807	50.6998	35.83464	-0.50063	0.038557	0.063677	0.161851	2-Keto-gl	2-Keto-glutaramic ac		0.866	145.0369	C5H7NO	18465-19-5	4	Keto acid	C00940	ko00250//	Amino ac	Metabolism
POS00047	170.6121	371.315	174.9564	131.839	186.9934	341.6381	22.09915	119.2769	26.64816	75.0031	43.314	23.53615	229.559	51.64625	-2.15213	0.002331	0.006098	0.609655	Indole-3-	4-Indolecarbaldehyc		3.255	145.0523	C9H7NO	487-89-8	3	Purine nu	-	-	-	-

POS00048	294.8154	168.5048	222.0104	179.2235	135.7389	171.0871	243.4095	227.2924	224.5782	222.0945	182.2788	214.2191	195.23	218.9788	0.165616	0.352615	0.423916	0.095015	Allysine	Allysine	0.86	145.0734	C6H11N(	1740725	3	Carboxyli	C04076	ko01100//	Amino ac	Metabolism
POS00049	143.4643	18.57507	106.6628	105.8122	109.7756	22.38569	35.98181	12.28934	15.40382	12.18599	82.06134	64.6256	84.44593	37.09132	-1.18695	0.080191	0.118119	0.252461	Spermidi	Spermidine	0.598	145.1573	C7H19N3	124-20-9	4	Organoni	C00315	ko01100//	Amino ac	Environmental Information Processing;Metabolism
POS00050	649.7729	403.9493	522.2867	459.9042	308.1138	408.1001	257.3972	350.3191	285.0685	228.2986	435.8852	297.3911	458.6878	309.06	-0.56963	0.024797	0.043817	0.509695	Oxogluta	alpha-Ketoglutaric a	1.224	146.0212	C5H6O5	328-50-7	2	Keto acid	C00026	ko01100//	Amino ac	Metabolism
POS00051	20.46335	16.90934	10.53449	6.328527	10.26212	10.63683	47.50958	537.4904	38.64602	33.15432	43.00451	52.57739	12.52244	125.397	3.323915	0.201169	0.265251	0.346714	Coumarir	Cumarin	2.303	146.0362	C9H6O2	91-64-5	3	Coumarir	C05851	ko01110//	Biosynthe	Metabolism
POS00052	5903.149	10549.08	11760.65	9336.125	7414.702	8351.599	7647.166	6591.884	2808.127	18105.23	8639.281	3375.166	8885.884	7861.143	-0.17678	0.680718	0.725065	0.351553	L-Glutam	DL-Glutamine	0.888	146.0685	C5H10N2	56-85-9	4	Carboxyli	C00064	ko01100//	Amino ac	Environmental Information Processing;Genetic Information Processing;Metabolism
POS00053	756.8134	487.2343	493.7944	444.0086	355.6794	425.3543	981.9709	890.9201	856.2896	712.1297	1193.137	851.2532	493.8141	914.2835	0.888674	0.000683	0.002351	0.997788	L-Lysine	DL-Lysine	0.687	146.1049	C6H14N2	56-87-1	4	Carboxyli	C00047	ko01100//	Amino ac	Environmental Information Processing;Genetic Information Processing;Metabolism
POS00054	5152.525	3732.65	4379.335	3579.009	2523.523	3508.914	3774.609	2894.102	2746.89	4497.504	4258.266	3609.608	3812.659	3630.163	-0.07076	0.701528	0.742844	0.226118	L-Glutam	L-Glutamic acid	0.8	147.0525	C5H9NO	56-86-0	4	Carboxyli	C00025	ko01100//	Amino ac	Environmental Information Processing;Genetic Information Processing;Metabolism
POS00055	12213.06	7338.08	7389.719	4361.251	17548.3	6139.571	3883.924	4049.851	1299.386	1876.257	2746.237	2866.986	9164.996	2787.107	-1.71737	0.010594	0.02062	3.574226	Fagomine	(4R)-5-Hydroxy-L-le	0.862	147.0891	C6H13N(	53185-12-9	3	Piperidin	-	-	-	-
POS00056	29.99153	12.89943	18.88572	18.32584	8.260855	12.84089	9.762713	7.359123	8.04158	7.988235	26.8597	8.154057	16.86738	11.3609	-0.57016	0.237406	0.305637	0.066605	Cuminalc	EL9600000	2.272	148.0882	C10H12O	122-03-2	4	Prenol lip	C06577	ko01120//	Global an	Metabolism
POS00057	866.705	838.0954	899.5859	514.7655	461.2676	708.2691	917.4646	1018.074	1134.785	660.6202	1378.952	1022.697	714.7814	1022.099	0.51596	0.032207	0.054843	0.729199	L-Methio	L-(-)-Methionine	0.858	149.0505	C5H11N(	63-68-3	3	Carboxyli	C00073	ko01100//	Amino ac	Genetic Information Processing;Metabolism
POS00058	30.35647	10.38248	17.69534	16.50722	41.54443	9.696004	95.38782	106.0854	50.58277	33.81083	99.17031	50.55058	21.03032	72.59794	1.787458	0.003642	0.008693	0.345138	Asparagu	asparagusic acid	1.257	149.9802	C4H6O2S	118374	4	Dithiolan	-	-	-	-
POS00059	35.02563	99.69125	23.69794	18.4273	54.79783	155.1317	25.36134	24.5511	18.7252	13.87845	20.84518	1.756537	64.46193	17.51964	-1.87947	0.059145	0.091197	0.258149	Guanine	Guanine	1.47	151.0489	C5H5N5(	73-40-5	3	Imidazop	C00242	ko01100//	Global an	Metabolism
POS00060	17.85928	10.65673	13.13325	10.18685	8.657664	11.93122	21.46964	19.37288	20.35106	14.12289	20.70457	18.31877	12.07083	19.05663	0.658768	0.002141	0.005787	0.118632	p-Hydroxyphenylacetic acid		1.223	152.0459	C8H8O3	156-38-7	9	Phenols	C00642	ko01120//	Amino ac	Metabolism
POS00061	4.433192	31.11802	32.70562	3.645472	23.2275	24.40588	31.22217	33.68766	27.63109	25.29317	32.95627	28.76176	19.92261	29.92535	0.586961	0.094158	0.135638	0.125251	(-)-trans-(	5-Isopropyl-2-methy	2	152.1195	C10H16O	2102-58-1	4	Prenol lip	C00964	ko01110//	Global an	Metabolism
POS00062	96.69422	53.34929	50.63626	50.37829	47.16477	51.90268	188.2432	179.5414	108.732	182.0823	181.1386	144.93	58.35425	164.1113	1.491764	3.29E-05	0.000299	0.523762	L-Histidi	L-Histidine	0.694	155.0689	C6H9N3(	71-00-1	3	Carboxyli	C00135	ko01100//	Amino ac	Environmental Information Processing;Genetic Information Processing;Metabolism
POS00063	1977.048	1692.52	1678.954	1468.319	1402.282	1744.204	1717.933	1987.731	1899.553	1758.962	2414.991	1443.335	1660.554	1870.417	0.171695	0.21149	0.275527	0.507309	Phosphog	phosphoglycolate	14.512	155.9829	C2H5O6F	13147-57-4	3	Organic p	C00988	ko01100//	Carbohyd	Metabolism
POS00064	17.37523	49.01314	18.47432	13.26744	7.149943	62.10533	30.01062	38.61451	21.62967	21.3831	39.33581	23.40771	27.89757	29.06357	0.059073	0.90639	0.934983	0.025575	6-Methox	6-Methoxyquinoline	2.791	159.0677	C10H9N(	5263-87-6	3	-	-	-	-	-
POS00065	105.3211	44.20419	93.36255	75.20389	33.40604	43.75532	75.78998	73.26876	49.20441	61.54724	63.91123	51.91546	65.87551	62.60618	-0.07344	0.805135	0.843843	0.033183	Calystegi	Guvacoline	0.859	159.0887	C7H13N(	131580-36-	5	Tropane a	C10850	ko00960//	Biosynthe	Metabolism
POS00066	59.04901	38.04528	53.05317	48.23513	26.85423	33.87607	35.9949	48.03328	34.12725	31.59242	61.22931	26.6147	43.18548	39.59864	-0.1251	0.630625	0.682829	0.027897	L-2-Amin	.alpha.-Aminoadipic	1.221	161.0681	C6H11N(	1118-90-7	4	Carboxyli	C00956	ko01100//	Amino ac	Metabolism
POS00067	3585.952	2850.666	3459.787	2784.751	1960.917	3034.275	4873.119	4434.348	3494.036	3803.373	5565.155	4824.727	2946.058	4499.126	0.610859	0.0026	0.006721	1.924889	Levogluc	Levoglucosan	0.829	162.0521	C6H10O5	498-07-7	4	Oxepanes	-	-	-	-
POS00068	2127.344	1501.15	827.5239	1744.649	527.4563	677.9955	2744.998	2418.412	1621.397	2818.359	2645.629	2585.582	1234.353	2472.396	1.002155	0.003101	0.007682	1.642431	D-1,5-Anl	1,5-Anhydro-L-arab	1.099	162.0521	C6H10O5	75414-43-6	4	Oxanes	-	-	-	-
POS00069	14.8482	5.678948	7.549314	7.147268	8.454976	5.352548	11.41592	45.98167	3.64558	8.752113	5.973207	17.43864	8.171876	15.53452	0.926739	0.287166	0.358409	0.078571	Methyl ci	3-Methyl-3-sulfanyll	1.892	162.0707	C10H10O	103-26-4	16	Cinnamic	-	-	-	-
POS00070	62.62342	9.981695	17.09948	14.68207	21.76893	16.91929	36.64637	20.60914	24.34672	11.73526	42.60076	32.61404	23.84581	28.09205	0.236426	0.653028	0.702435	0.034071	L-Nicotin	(+/-)-nicotine	14.473	162.1151	C10H14N	20033	3	Pyridines	C16150	ko01120//	Global an	Metabolism
POS00071	125.1136	85.41032	71.73896	61.89757	39.76342	77.13481	38.46201	17.48554	35.21839	47.29449	41.23373	31.6605	76.84311	35.22578	-1.12528	0.007	0.014763	0.301803	Homome	homomethionine	0.712	163.066	C6H13N(	25148-30-5	4	Carboxyli	C17213	ko01110//	Biosynthe	Metabolism
POS00072	181.676	111.3944	139.2048	113.0002	101.0363	113.409	338.7923	374.7769	243.1913	268.7285	378.0491	253.2748	126.6201	309.4688	1.289287	6.58E-05	0.000444	0.689449	Ethionine	L-Ethionine	1.226	163.066	C6H13N(	13073-35-3	4	-	-	-	-	-
POS00073	1773.141	944.6804	997.938	789.7015	661.4187	958.0963	2226.72	2063.873	1848.162	1939.032	2334.767	1782.727	1020.829	2032.547	0.993547	0.000241	0.001161	1.534944	Phenylpy	3-(3,4-dihydroxyphe	1.248	164.0467	C9H8O3	156-06-9	3	Benzene a	C00166	ko01100//	Amino ac	Metabolism
POS00074	132.2058	77.02722	20.21085	2.516539	53.47943	20.22601	495.8325	641.8776	536.6687	338.1331	429.8839	521.7685	50.94431	494.0274	3.277598	2.39E-06	4.95E-05	1.042744	2-Hydrox	2-Hydroxycinnamic	2.101	164.0467	C9H8O3	583-17-5	3	Cinnamic	C01772	ko01110//	Amino ac	Metabolism
POS00075	49.93052	31.57606	7.165778	19.38171	21.41636	24.34058	1155.385	860.6103	983.133	1292.933	2488.274	1410.427	25.63517	1365.127	5.734767	0.000227	0.001123	1.895633	p-Couma	4-Coumaric acid	2.649	164.0468	C9H8O3	501-98-4	3	Cinnamic	C00811	ko01100//	Amino ac	Metabolism
POS00076	717.7842	411.2525	530.5431	443.588	333.0428	496.5307	318.6196	347.666	299.9778	346.5546	384.3738	401.8783	488.7902	349.845	-0.4825	0.032369	0.054843	0.505901	S-(2-Amir	S-Aminoethyl-L-cys	0.643	164.0612	C5H12N2	2936-69-8	5	Carboxyli	-	-	-	-
POS00077	71.45608	33.13678	40.41494	42.94848	27.01275	38.45842	82.42325	51.98816	98.20356	43.80546	73.76965	53.78662	42.23791	67.32945	0.672699	0.039714	0.064933	0.183427	cis-Jasmo	Fenipentol	4.526	164.1195	C11H16O	NA	3	Organoox	-	-	-	-
POS00078	279.1197	158.9732	171.2754	224.2318	148.0732	158.7676	62.71494	46.94196	24.54335	42.39657	38.96556	42.83403	190.0735	43.06607	-2.14193	4.60E-05	0.000367	0.612061	DL-Methi	L-Methionine sulfox	0.8	165.0454	C5H11N(	3226-65-1	3	Carboxyli	-	-	-	-
POS00079	84.32182	54.18582	59.68844	55.61679	34.22984	50.47627	132.1996	112.3814	81.50977	96.73924	115.0098	93.92879	56.41983	105.2948	0.900159	0.000604	0.002219	0.340178	1-Methyl	Epiguanine	1.226	165.0644	C6H7N5(	938-85-2	3	Imidazop	-	-	-	-
POS00080	9205.581	5044.203	5490.648	4670.363	4193.405	5209.045	13074.55	11602.37	9866.129	11923.72	13800.02	9747.007	5635.541	11668.97	1.050051	0.000124	0.000709	3.854208	L-Phenyl	L-Phenylalanine	1.678	165.0783	C9H11N(	63-91-2	4	Carboxyli	C00079	ko01100//	Amino ac	Environmental Information Processing;Genetic Information Processing;Metabolism
POS00081	964.0398	527.2129	581.5244	516.1481	422.3859	521.7967	1370.847	1141.725	985.6212	1224.656	1420.172	981.9644	588.8513	1187.498	1.011949	0.000264	0.001218	1.205026	1-Deoxy-l	3-(4-Methyl-3-pente	1.679	166.0814	C6H14O5	18545-96-5	16	Organoox	-	-	-	-
POS00082	28.48651	14.50052	15.87431	14.44357	21.24081	15.12104	50.99533	55.51859	46.17415	37.46868	67.78553	31.85043	18.27779	48.29879	1.401895	0.000385	0.00161	0.272347	Myrtenic	(2E,4E,7E)-2,4,7-Dec	3.276	166.0988	C10H14O	NA	3	-	C11940	ko00903//	Metabolis	Metabolism
POS00083	111.8009	69.06653	81.74741	86.13869	31.05384	64.59485	53.89319	59.82899	60.61279	47.99707	65.5324	47.29704	74.06704	55.86025	-0.40701	0.139753	0.192446	0.159002	Pyridoxal	Pyridoxal	0.855	167.0576	C8H9NO	66-72-8	3	Pyridines	C00250	ko01100//	Global an	Metabolism
POS00084	161.1707	77.51964	120.8423	132.9341	11.85977	58.37957	2.340543	0.904834	0.943957	1.801875	1.753461	1.594039	93.78433	1.556451	-5.91301	0.002079	0.005712	0.457974	Homoger	MFCD00189386	2.556	168.0418	C8H8O4	451-13-8	2	Phenols	C00544	ko01100//	Amino ac	Metabolism
POS00085	135.6107	89.53334	95.30613	85.81508	60.71962	84.77704	66.19852	75.4827	85.73971	72.92605	73.54425	84.94717	91.96031	76.47307	-0.26606	0.168971	0.229268	0.159159	Norepine	noradrenaline	1.225	169.0731	C8H11N(	51-41-2	4	Phenols	C00547	ko01100//	Amino ac	Metabolism
POS00086	6.804875	4.939631	4.693429	5.174544	1.343597	5.264044	30.28916	45.65955	38.60164	42.84373	37.27594	42.49983	4.703353	39.52831	3.071125	3.85E-08	6.30E-06	0.301599	Pyridoxir	PYRIDOXINE	1.805	169.0732	C8H11N(	65-23-6	4	Pyridines	C00314	ko01100//	Global an	Metabolism
POS00087	265.9338	172.1544	190.8242	178.3876	101.3876	152.0459	194.8318	138.1073	134.3458	149.8858	178.2397	145.5191	176.7889	156.8216	-0.1729	0.426398	0.4962	0.124706	Glycylprc	Gly-l-pro	0.848	172.084	C7H12N2	704-15-4	4	Carboxyli	-	-	-	-
POS00088	505.6544	267.6812	535.1641	406.7315	374.6588	435.111	204.3715	280.5356	227.7128	177.1257	298.9876	235.1753	420.8335	237.3181	-0.82643	0.001772	0.004994	0.629458	Aconitic a	trans-Aconitic acid	1.224	174.0159	C6H6O6	4023-65-8	3	Carboxyli	C02341	ko00660//	Carbohyd	Metabolism
POS00089	108.9265	68.26261	77.35982	57.63528	32.17777	68.91271	99.53284	106.9386	113.4012	108.9647	105.481	94.60691	68.87912	104.8209	0.605787	0.006916	0.014686	0.248558	L-Arginir	L-(+)-Arginine	0.937	174.111	C6H14N4	74-79-3	3	Carboxyli	C00062	ko01100//	Amino ac	Environmental Information Processing;Genetic Information Processing;Metabolism
POS00090	982.9221	3235.343	830.8562	574.05	449.7055	5636.668	3.115801	8.226442	6.018646	8.884777	2.54801	4.224764	1951.591	5.503073	-8.4702	0.044648	0.071921	1.719184	3-Indolea	1-methoxyindole-3-c	4.511	175.0628	C10H9N(	87-51-4	3	Indoles ar	C00954	ko01100//	Amino ac	Environmental Information Processing;Metabolism
POS00091	16.61394	19.1449	13.64152	13.34254	28.58385	21.37203	16.39217	17.99548	8.197811	10.31839	15.2078	10.28719	18.78313	13.06648	-0.52357	0.072116	0.108173	0.083507	4-Methyl	hymecromone	2.505	176.0465	C10H8O3	90-33-5	4	Coumarir	-	-	-	-
POS00092	2.844516	0.611311	1.09415	0.571812	1.118115	0.652292	0.785586	1.068595	1.199795	1.896959	1.921266	1.233501	1.148699	1.35095	0.233973	0.624329	0.678258	0.006883	Serotonin	Serotonin	14.509	176.0943	C10H12N	50-67-9	3	Indoles ar	C00780	ko01100//	Amino ac	Metabolism
POS00093	148.9953	75.33004	126.6598	97.74563	130.3161	155.8572	163.6837	1043.457	150.9903	403.5127	178.5288	2281.981	122.484	703.6923	2.522351	0.123115	0.172784	0.837341	DL-Sulfo	Sulforaphane	2.94	177.0275	C6H11N(	4478-93-7	3	Sulfoxide	-	-	-	-
POS00094	1.7554	1.03614	1.995933	1.180858	0.807599	2.31728	30.54206	22.81583	6.870049	9.011701	19.18796	20.13566	1.515535	18.09388	3.577602	0.001009	0.003054	0.201715	Alliin		1.625	177.0451	C6H11N(	556-27-4	4	Carboxyli	-	-	-	-
POS00095	778.4575	558.6598	521.7596	586.5735	397.0519	504.5942	65.3336	125.2719	92.26364	87.13898	107.1491	81.27717	557.8494	93.07241	-2.58345	4.54E-06	7.42E-05	1.111345	Gluconol	D-Glucono-delta-lac	1.176	178.0471	C6H10O6	90-80-2	3	Organoox	C00198	ko01100//	Carbohyd	Metabolism
POS00096	1714.439	1357.825	2079.62	1454.296	1165.099	1560.405	2785.625	2673.338	2028.118	2095.643	3298.08	2824.794	1555.28	2617.6	0.75107	0.001113	0.003308	1.633455	D-Glucos	3-hydroxy-3-methyl	0.829	179.0785	C6H13N(	3416-24-8	4	Carbohyd	C00329	ko01100//	Carbohyd	Metabolism
POS00097	439.1688	293.2779	287.6834	237.3926	227.0803	272.7172	1706.307	755.8633	416.7057	1960.233	3040.617	1626.742	292.8867	1584.411	2.435532	0.006901	0.014686	1.78112	Caffeic A	Caffeic acid	2.358	180.0416	C9H8O4	331-39-5	3	Cinnamic	-	-	-	-
POS00098	24.44071	16.09687	14.93978	17.84431	9.870035	22.05946	21.1587	14.04775	16.09453	18.52196	24.57737	16.09942	17.54186	18.41662	0.070207	0.748406	0.789447	0.020848	Jasmolon	3-BHA	2.619	180.1143	C11H16O	54383-66-3	4	Organoox	-	-	-	-
POS00099	1572.405	845.7382	882.09	702.8582	584.0652	858.7733	1994.696	1852.571	1643.102	1732.376	2084.88	1575.533	907.6549	1813.86	0.998847	0.000239	0.001161	1.454466	L-Tyrosin	L-Tyrosine	1.245	181.0732	C9H11N(	60-18-4	3	Carboxyli	C00082	ko01100//	Amino ac	Genetic Information Processing;Metabolism
POS00100	243.8464	257.4724	143.8584	120.6891	559.9537	236.0256	35.65612	44.23232	24.1912	12.31746	44.01426	15.03575	260.3076	29.24119	-3.15414	0.00498	0.011153	0.686823	Phosphoc	Phosphocholine	0.794	183.0653	C5H14N(	107-73-3	4	Organoni	C00588	ko01100//	Global an	Metabolism
POS00101	2115.413	2267.761	1687.152	1425.706	1409.35	1879.163	1921.053	2174.672	1733.829	1682.68	2489.943	1828.098	1797.424	1971.713	0.133518	0.384764	0.455862	0.412488	Phosphol	3-Phosphonooxypyr	14.504	183.9776	C3H5O7F	3913-50-6	1	Organoox	C03232	ko01100//	Amino ac	Metabolism
POS00102	542.0247	327.1435	421.9795	380.62	219.7362	317.7638	245.4805	207.3868	197.8688	200.9104	223.1507	236.5936	368.2113	218.5651	-0.75247	0.007933	0.016213	0.565136	Alanylprc	L-Alanyl-L-proline	0.853	186.0996	C8H14N2	13485-59-1	4	Carboxyli	-	-	-	-
POS00103	1877.212	652.9731	961.029	787.5505	784.3867	843.1343	2445.776	2555.569	2388.548	2470.532	2977.629	2213.277	984.381	2508.555	1.349568	2.84E-05	0.000274	1.937392	Indoleacr	trans-3-Indoleacrylic	1.931	187.0625	C11H9N(	1204-06-4	4	Indoles ar	-	-	-	-
POS00104	52.73545	39.49717	36.54331	40.01405	28.54257	33.08113	37.24699	36.57169	39.76615	43.98583	44.75076	46.68166	38.40228	41.50051	0.111937	0.430767	0.497741	0.039431	Nonaned	Azelaic acid	3.088	188.1041	C9H16O4	123-99-9	4	Fatty Acy	-	-	-	-
POS00105	387.1603	262.038	257.7306	231.6556	211.0025	221.303	152.9244	135.5827	136.5808	123.1193	91.17453	101.8688	261.815	123.5418	-1.08355	0.000592	0.0022	0.598386	Glycyl-L-	Glycyl-L-leucine	1.754	188.1155	C8H16N2	869-19-2	3	Carboxyli	-	-	-	-
POS00106	255.8168	130.7288	203.9931	164.044	136.7363	172.4139	71.37004	78.62206	59.85048	68.17666	84.39201	50.2067	177.2888	68.76966	-1.36626	0.00026	0.001217	0.515478	Alanyl-Va	Ala-Val	1.269	188.1156	C8H16N2	3303-45-5	2	-	-	-	-	-
POS00107	4.947621	3.655756	2.78162	2.426263	1.41339	3.867548	105.5331	91.06505	78.07763	85.65445	122.9663	82.97433	3.182033	94.37847	4.890437	1.19E-07	9.71E-06	0.505487	Kynureni	Kynurenic acid	2.048	189.042	C10H7N(	492-27-3	3	Quinoline	C01717	ko01100//	Amino ac	Metabolism
POS00108	109.8328	102.365	111.994	89.6467	61.08751	87.45359	106.2034	119.1527	76.85537	142.6499	118.6729	88.57128	93.72994	108.6843	0.213561	0.25379	0.324177	0.131538	Diaminop	DL-alpha,epsilon-Di	0.803	190.0946	C7H14N2	583-93-7	3	Carboxyli	C00666	ko01100//	Amino ac	Metabolism
POS00109	43.96077	22.60156	32.65314	29.66883	56.59347	20.90773	45.25729	29.65393	47.14637	18.41767	19.55271	22.59218	34.39758	30.43669	-0.1765	0.615773	0.673438	0.062316	5-Hydrox	5,6-Dihydroxy-3-me	3.213	191.0576	C10H9N(	54-16-0	3	Indoles ar	C05635	ko01100//	Amino ac	Metabolism
POS00110	6237.338	3897.746	5228.37	4491.871	3529.766	3707.538	2382.4	3470.63	3133.047	1702.462	3734.96	3033.665	4515.438	2909.527	-0.63408	0.012095	0.023129	1.790597	Citric acic	Citric acid	0.891	192.0263	C6H8O7	77-92-9	3	Carboxyli	C00158	ko01100//	Amino ac	Metabolism
POS00111	2.721411	2.283754	1.681001	2.096787	0.846874	0.971613	9.390107	4.566569	2.769195	15.95534	67.2842	12.87353	1.766907	18.80649	3.411933	0.116201	0.164493	0.181839	Scopoleti	7-hydroxy-6-methox	2.369	192.0416	C10H8O4	92-61-5	3	Coumarir	C01752	ko01110//	Biosynthe	Metabolism
POS00112	795.2299	589.653	642.5071	614.2856	350.2834	508.7253	55.8802	173.4378	146.5967	134.1154	179.1286	107.9442	583.4474	132.8505	-2.1348	3.18E-05	0.000297	1.076498	Quinic ac	1,3,4,5-Tetrahydroxy	0.863	192.0627	C7H12O6	77-95-2	3	Quinoline	C00296	ko00400//	Amino ac	Metabolism
POS00113	88.08198	46.76557	59.17484	61.826	47.02826	66.74221	17.29172	16.0631	34.14186	20.93764	17.89257	28.30916	61.60314	22.43934	-1.45697	0.000205	0.001045	0.320964	2,6-Diisop	2,6-Diisopropyl-3-m	7.993	192.1508	C13H20O	28434-93-7	3	Prenol lip	-	-	-	-
POS00114	44.09594	158.1567	24.15605	120.7663	14.55197	75.38088	4442.689	5442.142	4986.566	4778.071	7730.612	4629.936	72.85132	5335.003	6.19439	9.88E-07	2.94E-05	3.816814	ferulic aci	Ferulic acid	2.738	194.0572	C10H10O	1135-24-6	3	Cinnamic	C01494	ko01100//	Biosynthe	Metabolism
POS00115	173.8879	84.40546	63.65533	60.18014	107.3715	62.28744	221.02	233.2096	170.541	86.4951	291.6379	94.34554	91.96463	182.8748	0.991706	0.036872	0.061831	0.404397	Neocnidil	Sedanolide	4.405	194.13	C12H18O	4567-33-3	3	Lactones	-	-	-	-
POS00116	3597.406	2987.524	2793.053	2562.298	2412.302	2804.354	343.7431	674.9438	455.7048	337.793	606.9892	306.2258	2859.49	454.2333	-2.65425	1.08E-07	9.71E-06	2.551582	Gluconic	D-Gluconic acid	0.819	196.0575	C6H12O7	526-95-4	3	Organoox	C00257	ko01100//	Carbohyd	Metabolism
POS00117	12.79536	6.966053	5.65243	7.512498	10.84582	4.656519	13.62776	4.801308	7.076255	11.57186	9.81806	6.415448	8.071446	8.885115	0.138563	0.673853	0.720098	0.016405	dodecana	Lauramide	7.308	199.1929	C12H25N	NA	3	-	-	-	-	-
POS00118	202.3506	121.7122	169.9653	155.8057	86.62841	104.0889	122.5173	108.1149	119.6371	123.3344	92.9096	111.7019	140.0918	113.0359	-0.30959	0.172611	0.232278	0.204527	Coprine	Coprine	0.851	202.0943	C8H14N2	58919-61-2	4	Carboxyli	-	-	-	-
POS00119	546.8534	422.4232	417.3097	382.0946	359.1984	405.7761	135.4978	96.79085	176.0497	127.0524	121.9267	89.73324	422.2759	124.5085	-1.76194	1.48E-06	3.45E-05	0.905328	Alanyl-Is	Ala-Ile	1.75	202.1311	C9H18N2	29727-65-9	3	Carboxyli	-	-	-	-
POS00120	4.228687	2.275795	3.005403	2.526082	2.887075	1.708796	9.225067	14.96049	7.079584	7.802238	9.39043	3.66991	2.771973	8.687953	1.648103	0.003425	0.008236	0.112862	Glycyl-Gl	Glycylglutamine	9.08	203.0896	C7H13N3	NA	4	Carboxyli	-	-	-	-
POS00121	1407.529	1040.822	1016.733	880.7983	735.6011	791.3833	1221.09	1194.899	1222.156	1124.206	1465.947	996.2479	978.811	1204.091	0.298842	0.083424	0.121784	0.547924	3-Butylid	(Z)-3-butylidene-7-h	8.693	204.0778	C12H12O	93236-67-0	4	Isobenzof	-	-	-	-
POS00122	1990.945	722.6718	1045.154	866.7262	848.887	948.4873	2479.413	2625.684	2444.249	2455.363	3016.954	2244.63	1070.479	2544.382	1.249059	4.84E-05	0.000368	1.892387	L-Trypto	DL-Tryptophan	1.93	204.089	C11H12N	73-22-3	4	Indoles ar	C00078	ko01100//	Amino ac	Genetic Information Processing;Metabolism
POS00123	465.8602	322.6915	355.7402	304.7095	219.0878	323.5957	256.136	250.6352	250.1253	254.1353	279.2778	254.3891	331.9475	257.4498	-0.36666	0.047743	0.075786	0.365283	N6-Acety	N~6~-Acetyl-5-hydr	1.227	204.1103	C8H16N2	NA	3	Carboxyli	-	-	-	-
POS00124	1.809447	0.978268	1.20772	1.014531	0.862365	0.919278	2.370313	2.608032	1.478728	2.966816	3.474933	3.516623	1.131935	2.735908	1.273228	0.000899	0.002827	0.06269	Zeanic ac	telmesteine	1.241	205.04	C10H7N(	30536-55-1	12	Quinoline	-	-	-	-
POS00125	15.59417	22.55955	29.81895	21.19752	48.70319	86.54524	45.43258	115.1598	16.59784	38.54979	74.03624	61.27504	37.4031	58.50854	0.645489	0.259298	0.327376	0.171854	1-Isothioc	1-Isothiocyanato-6-(	3.949	205.0587	C8H15N(	4430-35-7	4	Sulfoxide	-	-	-	-
POS00126	9.948954	8.15371	5.671076	2.800078	6.405484	4.10134	4914.445	3446.94	1762.88	3143.76	5391.295	3072.45	6.180107	3621.962	9.194924	5.58E-05	0.000396	3.164612	Scoparon	Sinapinic acid	2.693	206.057	C11H10O	120-08-1	4	Coumarir	-	-	-	-
POS00127	61.92082	30.11364	43.70257	41.72745	22.07791	28.69082	20.17682	14.14324	18.23766	23.7379	25.99322	21.53143	38.03887	20.63671	-0.88226	0.016864	0.031333	0.184553	Arenaine		2.272	207.1335	C11H17N	35471-10-4	17	Diazines	-	-	-	-
POS00128	346.8448	220.673	284.9877	254.8894	171.295	213.6687	160.6404	255.4277	179.948	141.6368	216.8625	184.9163	248.7264	189.9053	-0.38928	0.079865	0.118119	0.301313	Vanilpyruvic acid		1.224	210.056	C10H10O	1081-71-6	15	Benzene a	-	-	-	-
POS00129	1338.973	897.4596	886.8978	971.2116	738.9575	891.6045	112.4333	191.7884	151.5691	156.1846	185.3674	146.3968	954.1839	157.2899	-2.60084	2.51E-06	4.95E-05	1.460369	D-manno	alpha-D-manno-hep	1.175	210.0732	C7H14O7	60426-75-7	3	Organoox	-	-	-	-
POS00130	192.8857	259.9977	154.3647	170.5075	87.85898	261.9421	63.7425	41.39415	35.35162	38.80203	65.26303	45.64544	187.9261	48.36646	-1.95809	0.000505	0.001965	0.558968	Sedohept	Hept-2-ulose	0.837	210.0743	C7H14O7	3019-74-7	1	Organoox	C02076	ko00710//	Energy m	Metabolism
POS00131	141.2402	95.21699	130.7908	123.0127	69.45284	124.1742	48.19114	91.9365	50.70979	91.58178	66.35667	78.42312	113.9813	71.19983	-0.67885	0.009754	0.019099	0.290074	(-)-Jasmo	NP-000124	2.643	210.1249	C12H18O	6894-38-8	3	Fatty Acy	C08491	ko01100//	Global an	Environmental Information Processing;Metabolism
POS00132	124.2869	72.3158	81.56436	73.18797	56.71935	71.55309	126.0402	105.056	105.4215	113.6065	115.6592	94.56055	79.93791	110.0573	0.461303	0.016231	0.030329	0.223975	Vanillactic acid		0.723	212.0665	C10H12O	2475-56-1	9	Phenylpro	-	-	-	-
POS00133	1402.011	938.7257	717.7534	668.2666	1179.367	659.8524	3512.187	3280.141	2703.357	1417.475	4161.93	1666.854	927.6627	2790.324	1.58876	0.002232	0.005941	2.084127	cucurbic a	NP-002855	4.09	212.1404	C12H20O	58240-50-9	3	Fatty Acy	-	-	-	-

POS00134	20.64109	20.11309	14.31743	15.25398	13.86378	16.06934	12.91525	14.29535	17.60888	13.15214	19.83504	14.0165	16.70979	15.30386	-0.1268	0.41568	0.487194	0.032905	Tetradecy	1-Tetradecylamine	5.11	213.2447	C14H31N	2016-42-4	4	-	-	-	-	-
POS00135	627.3564	363.0745	407.8834	359.5747	371.6442	348.5887	495.4161	477.7361	575.9232	471.0953	505.3449	572.6448	413.0203	516.3601	0.322165	0.055113	0.086229	0.339214	Valylprol	Valylproline	1.558	214.131	C10H18N	20488-27-1	3	Carboxyli	-	-	-	-
POS00136	5.11376	2.735264	5.015181	3.729222	0.874141	4.391302	3.393818	5.816944	3.704606	4.02917	6.389572	4.088894	3.643145	4.5705	0.327168	0.28954	0.358635	0.031961	Succinylp	Succinyl proline	1.866	215.0785	C9H13N(	63250-32-8	4	-	-	-	-	-
POS00137	39.01332	59.66362	40.37689	44.638	51.83433	45.5497	24.33843	43.52345	84.78154	249.1734	71.66279	51.31671	46.84598	87.46606	0.900798	0.254885	0.324309	0.190807	N-Nonan	N-Nonanoylglycine	3.267	215.1514	C11H21N	NA	3	Carboxyli	-	-	-	-
POS00138	40.01672	2.267941	6.795318	2.903485	14.09159	9.927346	4.232635	2.601069	80.87268	1.86453	3.896222	1.84022	12.66707	15.88456	0.326543	0.825587	0.859768	0.018912	L-1,2,3,4-T	1,2,3,4-tetrahydro-be	4.598	216.0889	C12H12N	42438-90-4	4	Indoles ar	-	-	-	-
POS00139	155.6214	95.47525	127.5289	103.465	64.61989	87.46197	103.826	81.85727	87.06213	86.55699	110.9733	97.24013	105.6954	94.58596	-0.16021	0.440655	0.507373	0.093619	Prolyl-Th	pro-thr	0.855	216.11	C9H16N2	NA	4	Carboxyli	-	-	-	-
POS00140	65.83112	41.05175	45.29359	38.56622	28.39858	42.9265	47.81947	47.44874	43.93146	43.53579	50.49217	38.98262	43.67796	45.36837	0.054782	0.756401	0.795315	0.013296	β-Alanyl-	b-Ala-Lys	0.664	217.1419	C9H19N3	NA	3	-	-	-	-	-
POS00141	0.740992	0.57671	0.508156	0.82892	0.384036	0.400169	17.57977	27.87614	16.16507	12.32082	31.94664	12.54195	1	19.7384	4.302933	0.000199	0.001035	0.224414	Eupatoric	EUPATORIOCHRO	2.511	218.0935	C13H14O	19013-03-7	3	-	-	-	-	-
POS00142	290.0723	656.0199	598.2827	546.5856	480.4959	637.8576	490.0061	435.5728	447.7485	408.6145	448.1578	409.3224	534.8857	439.9037	-0.28204	0.125621	0.175547	0.301863	Pantother	Pantothenic acid	1.753	219.1101	C9H17N(	137-08-6	2	Alcohols	C00864	ko01100//	Global an	Metabolism
POS00143	824.3943	486.9511	728.902	661.5152	199.7169	506.427	572.9621	728.5897	438.9258	611.3633	759.5778	931.7475	567.9844	673.8611	0.2466	0.376429	0.447824	0.299045	(-)-Sedam	sedamine	2.695	219.1614	C14H21N	497-88-1	4	-	C10171	ko00960//	Biosynthe	Metabolism
POS00144	185.0015	148.9696	110.0328	113.5575	86.24731	132.8837	18.80928	19.5742	20.32661	15.67904	33.55857	22.74955	129.4487	21.78288	-2.57111	2.04E-05	0.000223	0.528558	Chrycorir	(-)-threo-isodihomo	0.879	220.0575	C12H12O	91362-90-2	7	Oxanes	-	-	-	-
POS00145	11.37565	9.84399	6.044814	3.554606	7.314616	4.926783	5784.668	4056.962	2066.728	3743.123	6532.736	3632.725	7.176743	4302.824	9.227739	6.66E-05	0.000444	3.449271	Sinapic ac	Sinapinic acid	2.699	224.0675	C11H12O	530-59-6	4	-	C00482	ko01100//	Biosynthe	Metabolism
POS00146	96.82756	42.91452	70.37066	84.16622	32.10807	36.86912	23.75371	14.24032	13.06776	24.72272	25.9832	12.98632	60.54269	19.12567	-1.66244	0.004415	0.010025	0.295417	Methyl ja	Methyl Jasmonate	2.551	224.1404	C13H20O	39924-52-2	3	Fatty Acy	C11512	ko01110//	Global an	Metabolism
POS00147	103.4454	51.07975	76.35041	67.98598	42.41211	74.02601	105.04	134.0118	69.50663	141.915	121.8841	139.3004	69.21661	118.6096	0.777031	0.006109	0.013498	0.321386	Allixin	3,4-Methylenesebaci	3.976	226.1196	C12H18O	125263-70-	4	Pyrans	-	-	-	-
POS00148	214.9368	102.9396	130.116	110.6054	55.50085	107.1219	353.7212	257.8705	374.3799	269.3919	349.0329	297.6865	120.2034	317.0138	1.399068	5.14E-05	0.000382	0.677273	Traumati	NP-008952	4.526	228.1352	C12H20O	6402-36-4	4	Fatty Acy	C16308	ko01110//	Global an	Metabolism
POS00149	1585.361	928.6555	958.6358	998.6155	839.5538	926.3712	455.165	1832.048	1714.757	1382.199	1608.165	1493.001	1039.532	1414.223	0.444075	0.135855	0.18824	0.611253	Leucylprc	Leucylproline	1.834	228.1465	C11H20N	6403-35-6	3	Carboxyli	-	-	-	-
POS00150	15.76972	21.4749	23.67239	52.60108	6.019521	64.0168	1.21608	2.266745	0.362555	0.365182	2.213627	0.396999	30.5924	1.136865	-4.75004	0.009679	0.019067	0.225783	Cyclobras	160492	6.878	234.0275	C11H10N	105748-58-	4	Indoles ar	-	-	-	-
POS00151	286.6381	187.8187	211.8142	203.3188	105.6121	163.7561	226.5757	190.1989	213.1714	276.7829	211.7331	194.0446	193.1596	218.7511	0.179496	0.373927	0.447824	0.115489	Glutamyl	glu-ser	0.79	234.0841	C8H14N2	5875-38-7	4	Carboxyli	-	-	-	-
POS00152	57.60651	38.66211	33.81952	30.91798	58.67996	30.22035	165.1902	166.9171	146.5642	61.45969	205.3437	83.68055	41.65107	138.1926	1.730255	0.00183	0.005114	0.474434	Valerenic	(9E)-9-Pentadecene-	4.091	234.161	C15H22O	609870	4	Prenol lip	-	-	-	-
POS00153	1.421818	0.997762	1.080228	0.837644	0.83093	0.928027	1.033833	0.969229	0.933162	0.932387	1.214463	1.035644	1.016068	1.019786	0.00527	0.971022	0.981317	0.000163	1-Hexade	Bis(2-ethylhexyl) am	6.056	241.2759	C16H35N	143-27-1	4	-	-	-	-	-
POS00154	715.8303	392.0582	640.2638	606.9077	280.2969	383.5557	362.3201	533.7187	497.5775	463.7164	518.7438	647.9527	503.1521	504.0049	0.002443	0.991762	0.991762	0.027354	Cytidine	Cytidine	0.825	243.0844	C9H13N3	65-46-3	4	Pyrimidir	C00475	ko01100//	Global an	Metabolism
POS00155	221.7254	125.6594	147.5497	145.3738	81.98787	110.879	165.248	209.399	175.132	160.3399	188.9135	175.4575	138.8625	179.0817	0.36696	0.079763	0.118119	0.221316	Uridine	Uridine	1.231	244.0685	C9H12N2	58-96-8	4	Pyrimidir	C00299	ko01100//	Global an	Metabolism
POS00156	70.21463	45.06185	66.00279	66.87947	28.32884	45.74371	71.9741	100.8614	79.68331	74.38814	97.69873	96.28239	53.70521	86.81467	0.692877	0.003174	0.007804	0.266564	D-Biotin	hexahydro-2-oxo-1h	0.824	244.0877	C10H16N	58-85-5	1	Biotin anc	C00120	ko01100//	Global an	Environmental Information Processing;Metabolism
POS00157	644.6428	394.9202	532.427	557.786	344.9767	421.0943	84.56289	105.1628	82.03987	109.85	91.28818	96.55897	482.6412	94.91045	-2.34631	8.49E-06	0.000116	1.006773	Leucyl-le	Leu-Leu	2.13	244.1779	C12H24N	3303-31-9	3	Carboxyli	-	-	-	-
POS00158	516.9739	287.1981	347.8188	299.5369	254.2267	314.1977	448.9234	383.9248	374.523	339.4158	389.8225	318.7697	336.6587	375.8965	0.159049	0.376611	0.447824	0.142487	Leucyl-As	Leucylasparagine	1.72	245.1366	C10H19N	NA	3	Carboxyli	-	-	-	-
POS00159	126.1831	80.35012	80.22353	68.76276	78.02197	75.69162	102.4037	105.9142	102.3964	77.87628	101.4984	80.00395	84.87219	95.01548	0.162871	0.328772	0.39966	0.073927	Valyl-Glu	Valylglutamine	1.667	245.1366	C10H19N	42854-54-6	3	Carboxyli	-	-	-	-
POS00160	134.1999	79.91725	114.0923	85.89079	81.65569	68.88651	25.27944	34.01916	48.79057	38.85525	30.48513	53.91877	94.10707	38.55805	-1.28727	0.00052	0.001976	0.379381	Methiony	met-pro	1.688	246.1029	C10H18N	NA	3	Carboxyli	-	-	-	-
POS00161	551.7185	329.7064	364.776	301.6952	278.3372	336.4339	272.4339	280.3519	290.7237	235.899	293.7549	252.3197	360.4445	270.9139	-0.41194	0.054831	0.0862	0.40954	Aspartyl-	asp-leu	1.825	246.1206	C10H18N	NA	3	Carboxyli	-	-	-	-
POS00162	43.92918	16.95775	23.31873	21.65479	16.24098	19.46823	41.19466	39.0649	31.71393	35.97281	39.78079	32.46312	23.59494	36.69837	0.637238	0.015746	0.029591	0.149868	Avocadie	2-(12-Tridecyn-1-yl)	7.68	246.1973	C17H26O	34227-08-2	4	Heteroarc	-	-	-	-
POS00163	48.39622	22.24622	37.48551	28.77757	16.43184	25.68689	195.231	153.4644	355.0602	138.1268	174.5251	174.3174	29.83738	198.4542	2.733613	0.000424	0.001712	0.585414	S-Acetyldihydrolipoamide		0.857	249.0842	C10H19N	NA	6	Fatty Acy	-	-	-	-
POS00164	6.342541	5.745491	3.510475	2.295093	5.087025	3.787017	63.77922	33.77804	49.51665	45.25508	64.28032	37.57699	4.461274	49.03105	3.458168	7.48E-06	0.000106	0.343962	Norambr	OPEO	8.727	250.1921	C16H26O	564-20-5	5	Naphthof	-	-	-	-
POS00165	226.7747	181.7249	203.9547	229.6985	57.8623	127.3603	120.9072	107.3073	81.76587	105.5143	141.9332	105.641	171.2292	110.5115	-0.63173	0.059302	0.091197	0.294436	Muramic	muramic acid	0.813	251.0993	C9H17N(	1114-41-6	4	Organoox	-	-	-	-
POS00166	237.414	1141.907	102.4001	98.88848	906.0615	1338.51	51.60337	24.88936	15.97762	23.82377	26.73175	24.3266	637.53	27.89208	-4.51457	0.02319	0.041666	1.009841	Deoxyade	2'-Deoxyadenosine	1.414	251.1008	C10H13N	958-09-8	4	Purine nu	C00559	ko01100//	Global an	Metabolism
POS00167	378.7062	252.0295	261.6321	252.5283	234.0022	262.3262	126.0435	162.9172	122.5702	123.6979	148.8745	121.0334	273.5374	134.1894	-1.02747	0.000106	0.000637	0.59486	Alanyl-Ty	Ala-Tyr	1.845	252.1099	C12H16N	3061-88-9	4	Carboxyli	-	-	-	-
POS00168	79.84245	60.26655	56.74025	42.30591	72.9408	41.37683	62.25109	106.2405	93.85094	20.72048	59.77611	18.70208	58.91213	60.25686	0.032561	0.935024	0.958473	0.011014	Nicotinat	D-ribosylnicotinate	1.152	255.0729	C11H13N	NA	5	Organoox	C05841	ko01100//	Global an	Metabolism
POS00169	312.074	148.6587	113.9341	101.4943	146.3647	123.9701	193.9283	199.9972	153.1607	130.6708	167.1729	107.4156	157.7493	158.7243	0.008889	0.978316	0.981317	0.028126	Palmitic a	Hexadecanamide	10.005	255.255	C16H33N	629-54-9	5	Fatty Acy	-	-	-	-
POS00170	195.4329	117.928	154.8021	138.1477	149.7084	120.6429	1.128551	0.956799	0.899873	0.656823	1.007026	0.850513	146.1103	1	-7.19091	1.94E-07	1.02E-05	0.628809	piscidic a	piscidic acid	1.447	256.0574	C11H12O	35388-57-9	3	Phenylpr	-	-	-	-
POS00171	59.29909	36.67123	41.77841	32.85528	38.40555	36.20001	32.92143	54.61756	39.25862	12.98532	38.28182	9.19833	40.86826	31.21051	-0.38895	0.256569	0.325187	0.104524	Pinocembrin		0.83	256.0764	C15H12O	480-39-7	11	Flavonoic	C09827	ko00941//	Biosynthe	Metabolism
POS00172	109.7969	88.55447	93.8038	74.33017	76.2636	101.1369	75.67169	101.5358	94.45347	117.2149	79.52446	79.33364	90.64763	91.289	0.010172	0.942762	0.963384	0.01513	Leucyl-Gl	leu-gln	1.662	259.1522	C11H21N	NA	3	Carboxyli	-	-	-	-
POS00173	229.1211	150.807	144.3945	130.2616	112.1662	140.0907	128.8377	142.7963	127.2551	137.1514	122.1891	110.7726	151.1402	128.167	-0.23786	0.21038	0.275177	0.184762	Lysyl-Leu	lys-leu	1.225	259.1883	C12H25N	7369-79-1	4	Carboxyli	-	-	-	-
POS00174	720.0234	564.6065	782.4113	602.9954	381.1069	522.4789	202.8424	199.6371	228.3261	115.5241	197.3027	116.0866	595.6038	176.6198	-1.75371	4.84E-05	0.000368	1.03277	Glucose 1	Glucose 1-phosphate	0.832	260.0286	C6H13O9	59-56-3	4	Organoox	C00103	ko01100//	Carbohyc	Metabolism
POS00175	966.8047	691.0155	673.0337	605.826	505.4533	685.6359	439.6539	497.777	492.1634	383.9473	442.58	407.9462	687.9615	444.0113	-0.63173	0.003897	0.009169	0.758765	gamma-G	gamma-Glutamylleu	1.799	260.1362	C11H20N	2566-39-4	3	Carboxyli	-	-	-	-
POS00176	212.6954	86.21219	122.8787	120.9054	130.3212	103.6127	76.41378	93.03295	140.7191	115.5603	137.581	104.0542	129.4376	111.2269	-0.21875	0.397989	0.469828	0.142044	Prolylphe	pro-phe	2.036	262.1306	C14H18N	13589-02-1	4	Carboxyli	-	-	-	-
POS00177	852.3097	480.8149	660.8172	678.3662	509.3595	537.2829	548.5287	497.184	288.0824	279.1529	427.2338	269.3155	619.8251	384.9162	-0.68732	0.011306	0.021876	0.665974	Ascorbala	4,5-Dihydroxy-4-(2,3	0.843	263.0629	C9H13N(	41679-87-2	4	Carboxyli	-	-	-	-
POS00178	48.42583	15.74697	28.60263	34.3296	18.15679	14.52317	22.02323	19.38004	13.42166	10.19861	18.1304	8.468839	26.63083	15.27046	-0.80235	0.080116	0.118119	0.132876	Thiamine		0.672	264.1033	C12H16N	59-43-8	4	Diazines	C00378	ko01100//	Folding, s	Environmental Information Processing;Genetic Information Processing;Metabolism
POS00179	41.21861	37.1765	34.58783	24.6256	47.73288	53.03445	139.7383	86.46056	88.57249	171.5446	136.6441	115.1739	39.72931	123.0223	1.630644	0.000144	0.000785	0.459974	Abscisic A	(±)-Abscisic acid	3.169	264.1326	C15H20O	7773-56-0	13	Prenol lip	C06082	ko01100//	Global an	Environmental Information Processing;Metabolism
POS00180	225.3334	141.7029	180.967	175.8145	94.20933	144.4921	185.626	256.9593	215.7113	238.944	198.8942	198.7319	160.4199	215.8111	0.427916	0.026326	0.045791	0.286446	Subaphyl	Vorinostat	2.117	264.1462	C14H20N	501-13-3	5	Cinnamic	C10497	ko00330//	Amino ac	Metabolism
POS00181	451.7038	314.1159	368.2907	325.9759	404.8674	252.3952	519.0677	559.6332	452.8159	347.2943	581.1615	602.4633	352.8915	510.406	0.532421	0.008879	0.017703	0.560147	7-Methoxy-2-methylisoflavone		0.856	266.0922	C17H14O	19725-44-1	8	Isoflavon	-	-	-	-
POS00182	279.3302	236.3137	259.9805	240.3077	144.7633	182.3583	438.034	320.821	320.5179	300.9104	374.0621	363.4171	223.8423	352.9604	0.657024	0.001255	0.003663	0.539394	Sapidolide A		0.797	266.1116	C14H18O	182191-87-	14	Lactones	-	-	-	-
POS00183	4658.24	2308.114	2682.507	2239.297	3631.223	1624.279	3223.587	4558.264	4117.674	2889.417	4886.495	4310.696	2857.277	3997.689	0.484526	0.065475	0.099582	1.237288	Adenosin	Adenosine	1.371	267.0954	C10H13N	58-61-7	5	Purine nu	C00212	ko01100//	Global an	Metabolism
POS00184	269.5284	246.1577	202.1473	270.6716	158.9552	209.2466	277.0968	224.4678	222.4479	255.0561	247.6713	220.4113	226.1178	241.1919	0.093107	0.472457	0.534579	0.100418	Capsi-am	Capsi-amide	10.43	269.2706	C17H35N	64317-66-4	4	Carboxyli	-	-	-	-
POS00185	1.150945	3.697608	0.791546	0.683429	0.564316	2.90823	3.620119	40.62672	0.761403	13.91765	65.4542	2.575915	1.632679	21.15933	3.695981	0.099545	0.142145	0.194594	Sarmento	Sarmentosin	1.598	275.0996	C11H17N	71933-54-5	3	Fatty Acy	-	-	-	-
POS00186	32.73553	24.1626	24.64094	17.46307	13.98489	22.38593	54.79288	42.38577	48.36246	34.78783	62.7381	36.26269	22.56216	46.55496	1.04503	0.000933	0.002889	0.236811	epsilon-(g	epsilon-(gamma-Glu	0.673	275.147	C11H21N	17105-15-6	4	Carboxyli	-	-	-	-
POS00187	174.2208	143.5826	87.46255	96.19918	122.8289	172.178	30.7366	35.38476	49.95424	18.0826	48.11858	19.95489	132.7453	33.70528	-1.97761	0.000109	0.000637	0.498904	6-Phosph	6-O-Phosphono-D-g	1.263	276.0238	C6H13O1	53411-70-4	3	Organoox	C00345	ko01100//	Carbohyc	Metabolism
POS00188	191.7159	134.6923	133.366	139.9585	81.48875	135.9601	172.8545	167.2851	156.622	214.6842	209.3881	126.0178	136.1969	174.4753	0.357328	0.080826	0.118521	0.238093	Gamma G	Glu-Glu	0.831	276.0945	C10H16N	1116-22-9	4	Carboxyli	-	-	-	-
POS00189	10.45575	8.523736	6.40332	6.712549	21.07006	7.764681	4.764963	5.388142	5.7981	2.624638	5.350913	3.64453	10.15502	4.595214	-1.14399	0.037313	0.062252	0.09699	Saccharopine		7.407	276.1295	C11H20N	997-68-2	9	Carboxyli	C00449	ko01100//	Amino ac	Metabolism
POS00190	2737.583	1274.641	1362.139	1329.11	3113.339	1763.746	6757.205	5500.387	4561.423	5795.936	6315.343	5168.584	1930.093	5683.146	1.558019	9.69E-06	0.000122	3.136734	Stearidon	Stearidonic acid	7.231	276.2076	C18H28O	20290-75-9	4	Fatty Acy	C16300	ko01110//	Global an	Metabolism
POS00191	62.93398	39.08515	42.24901	37.64174	29.59431	37.64643	12.34249	14.57288	18.67095	12.04994	14.52785	13.76559	41.5251	14.32162	-1.53579	0.000179	0.000944	0.266621	N-gamma	L-gamma-Glutamyl-	1.484	278.0928	C10H18N	17663-87-5	3	Carboxyli	-	-	-	-
POS00192	19305.42	14722.81	14120.38	14029.65	11595.81	13435.8	16727.58	15534.31	19131.27	13547.52	20661.58	15581.85	14534.98	16864.02	0.21442	0.150338	0.204836	1.597244	Diisobuty	Diisobutylphthalate	8.676	278.1504	C16H22O	84-69-5	5	Benzene a	-	-	-	-
POS00193	305.0974	166.8929	172.7209	158.0755	246.2364	240.2173	519.9974	493.6485	335.3058	400.1515	504.1735	328.1228	214.8734	430.2332	1.001632	0.000513	0.001973	0.724893	α-Linolen	?-linolenic acid	7.863	278.223	C18H30O	463-40-1	5	Fatty Acy	C06427	ko01100//	Global an	Metabolism
POS00194	174.7179	100.286	96.19909	92.94755	90.56482	99.94351	99.1896	115.6293	97.54395	90.68565	108.3353	87.23865	109.1098	99.77042	-0.1291	0.517278	0.57534	0.092201	Asparagi	asn-phe	1.855	279.1207	C13H17N	NA	4	Carboxyli	-	-	-	-
POS00195	194.3761	129.6878	116.3427	112.2243	111.4874	131.3431	70.9264	78.06149	102.6342	73.14881	71.03991	79.66182	132.5769	79.24544	-0.74243	0.003069	0.007661	0.36618	L-beta-as	L-alpha-Aspartyl-L-	1.955	280.1048	C13H16N	NA	4	Carboxyli	-	-	-	-
POS00196	77.44649	54.13513	90.98305	102.132	12.68118	42.80944	22.13383	10.99139	23.53351	19.34752	13.42063	15.88004	63.36454	17.55115	-1.85211	0.007562	0.015552	0.304656	Floribunc	2-Hydroxy-2-methyl	1.516	281.1461	C18H19N	3153-55-7	16	Aporphin	-	-	-	-
POS00197	240.5019	62.26419	86.41268	104.6769	383.2207	27.39043	146.0734	178.0747	38.31392	15.23108	80.321	52.37509	150.7445	85.06486	-0.82547	0.30752	0.37522	0.247606	Oleamide	Oleamide	10.253	281.2705	C18H35N	301-02-0	4	Fatty Acy	-	-	-	-
POS00198	17.50121	10.38255	9.425043	7.504574	26.87293	8.934398	14.28113	20.06144	33.19368	13.2279	14.26783	12.27791	13.43678	17.88498	0.412561	0.341913	0.412566	0.041941	Guanosin	Guanosine	1.414	283.0901	C10H13N	118-00-3	5	Purine nu	C00387	ko01100//	Global an	Metabolism
POS00199	12.04562	7.793208	8.04243	7.860773	9.068794	10.21033	8.874276	9.830549	8.081071	8.608137	7.311811	8.789843	9.170193	8.582615	-0.09553	0.463133	0.529526	0.025557	Stearamic	Stearamide	11.504	283.2861	C18H37N	124-26-5	4	Fatty Acy	-	-	-	-
POS00200	1048.189	825.0702	815.5841	686.2623	477.9142	839.2832	591.1798	445.3971	573.1661	627.6693	547.9997	638.3727	782.0505	570.6308	-0.4547	0.027951	0.048359	0.646112	Morphine	Glycylprolylhydroxy	1.57	285.1312	C17H19N	57-27-2	18	Morphina	C01516	ko01100//	Biosynthe	Metabolism
POS00201	18.99878	9.307272	11.17995	8.596274	11.60187	30.23584	379.0848	341.7884	271.8983	146.6036	331.1697	215.5889	14.98666	281.0223	4.228933	2.27E-05	0.00024	0.830898	Kaempfer	Kaempferol	2.366	286.0463	C15H10O	520-18-3	5	Flavonoic	C05903	ko01100//	Biosynthe	Metabolism
POS00202	305.2866	236.5893	223.3057	225.7721	182.6371	241.9247	281.1583	281.1461	325.425	234.1016	374.4753	276.0082	235.9193	295.3857	0.324307	0.042486	0.06912	0.310446	Hexadecanedioic acid		9.562	286.2129	C16H30O	505-54-4	5	Fatty Acy	C19615	ko01100//	Global an	Metabolism
POS00203	96.21883	60.75649	56.21278	59.12184	52.39529	56.93565	16.84173	23.00599	26.3276	17.7485	26.69074	25.46627	63.60681	22.68014	-1.48775	0.000138	0.000767	0.325367	Atropine		1.672	289.1624	C17H23N	55-48-1	18	Tropane a	C01479	ko01110//	Biosynthe	Metabolism
POS00204	428.9722	261.1733	317.218	274.1507	186.9273	266.6645	227.7546	232.4834	189.8636	177.483	264.8695	194.0586	289.1844	214.4188	-0.43156	0.061229	0.093561	0.34419	Sedoheptulose 7-phosphate		0.656	290.0438	C7H15O1	2646-35-7	12	Organic o	C05382	ko01100//	Carbohyc	Metabolism
POS00205	26.73522	13.37533	25.06691	22.21297	9.196567	11.48941	17.04099	11.47882	8.942452	13.30671	17.29387	11.51163	18.01273	13.26241	-0.44167	0.188995	0.251226	0.070093	N-gamma-Glutamyl-S-allylcys		1.544	290.0922	C11H18N	NA	4	Carboxyli	-	-	-	-
POS00206	24.33753	4.206255	16.91239	11.20562	4.760343	5.107116	143.6067	89.84816	53.6518	90.02391	155.0648	73.26724	11.08821	100.9104	3.185977	0.000305	0.001313	0.488467	Argininos	Argininosuccinic aci	0.784	290.1211	C10H18N	2387-71-5	5	Carboxyli	C03406	ko01100//	Amino ac	Metabolism
POS00207	2109.653	1399.245	1126.416	1012.617	1777.393	1000.969	5517.555	5081.217	4472.707	2303.281	6384.278	2584.547	1404.382	4390.597	1.644481	0.001528	0.004346	2.647011	(9R,13R)-	9S,13R-12-Oxophytc	4.09	292.2024	C18H28O	NA	4	-	-	-	-	-
POS00208	159.0936	96.15116	102.0894	102.879	100.6719	111.1172	63.25081	82.54211	64.45729	59.23085	77.23588	70.26975	112.0004	69.49778	-0.68846	0.00205	0.005681	0.313202	Glutamin	gln-phe	1.866	293.1363	C14H19N	92327-75-8	4	Carboxyli	-	-	-	-
POS00209	164.5984	112.0849	135.2616	105.5394	110.3805	110.8055	77.38991	83.53039	89.46125	63.86476	65.87539	74.3236	123.1117	75.74089	-0.70082	0.000899	0.002827	0.350953	Tyrosyl-H	Tyrosyl-4-hydroxyp	1.935	294.1202	C14H18N	936346-34-	4	Carboxyli	-	-	-	-
POS00210	175.6492	118.628	140.806	137.461	95.80623	135.2967	12.88103	13.38181	19.55893	16.43188	19.23044	16.6979	133.9412	16.36367	-3.03303	7.55E-07	2.72E-05	0.561961	Leucyl-Ty	Leucyltyrosine	2.045	294.1569	C15H22N	NA	3	Carboxyli	-	-	-	-
POS00211	2.259068	1.563382	1.648468	1.378878	1.201567	1.552067	632.724	338.8065	169.4919	861.055	1454.128	656.1557	1.600572	685.3935	8.742201	0.003952	0.009231	1.319479	Phaseolic	Phaseolic acid	2.364	296.0518	C13H12O	53755-04-7	4	Fatty Acy	-	-	-	-
POS00212	29.16593	26.05722	17.7525	16.60677	20.86841	17.63936	132.891	79.84848	54.40678	48.9339	130.5875	37.2469	21.34836	80.65244	1.917593	0.00637	0.013794	0.372378	Avenoleic	NP-008993	8.723	296.2335	C18H32O	177931-23-	5	Fatty Acy	-	-	-	-
POS00213	0.426225	0.291898	0.312143	0.261393	0.219935	0.266984	40.58991	33.86991	44.90541	56.4439	98.8357	61.72829	1	56.06219	5.808956	0.00016	0.000856	0.38516	Kuhlmannin		2.71	298.087	C17H14O	20972-79-6	9	-	-	-	-	-
POS00214	21.61456	7.70285	11.39498	8.483758	7.835315	7.09567	12.21866	15.19927	13.38597	13.42274	16.17734	13.41675	10.68785	13.97012	0.386372	0.192015	0.254206	0.052735	Sphingosi	THREO-SPHINGOS	9.523	299.2811	C18H37N	123-78-4	4	Organoni	C00319	ko01100//	Global an	Metabolism
POS00215	296.0324	87.72266	64.82166	68.54908	60.95506	78.4911	89.90242	75.74651	75.61307	67.26197	118.0561	90.67001	109.4287	86.20835	-0.34409	0.557322	0.615691	0.140233	Sphingan	Dihydrosphingosine	5.594	301.2967	C18H39N	764-22-7	4	Organoni	C00836	ko01100//	Global an	Metabolism
POS00216	1.616119	0.958884	2.744113	0.740389	0.620707	1.030484	57.39825	47.59975	79.99725	93.10066	124.7087	105.9472	1.285116	84.79197	6.043957	3.72E-05	0.000328	0.467065	Delphinidin		2.658	302.0386	C15H10O	528-53-0	13	-	C05908	ko01110//	Biosynthe	Metabolism
POS00217	45.21276	37.402	27.31408	21.46929	34.85029	27.65702	205.1043	161.8735	162.2868	138.7092	212.9588	139.8869	32.31757	170.1366	2.396303	1.30E-06	3.40E-05	0.610092	Diffutidir	4380	8.733	302.1158	C17H18O	NA	1	-	-	-	-	-
POS00218	182.4006	129.9773	100.6726	137.1584	98.17313	79.94536	183.5422	113.5724	92.91526	127.8429	83.116	49.09045	121.3879	108.3465	-0.16397	0.597701	0.655867	0.093009	Nicotianamine		0.791	303.1415	C12H21N	34441-14-0	5	-	-	-	-	-
POS00219	4.909178	3.874651	10.08227	3.114789	3.827292	3.183651	9.806276	8.940656	8.790179	8.40066	10.90065	23.39184	4.831972	11.70504	1.276446	0.024656	0.043817	0.108985	Musanolone C		0.814	306.091	C19H14O	NA	5	Naphthal	-	-	-	-

POS00220	141.3163	76.20705	155.7805	136.3237	55.315	113.3083	8.345613	11.5125	13.98329	13.92334	13.87432	11.38612	113.0418	12.17087	-3.21535	9.85E-05	0.000607	0.498212	5-O-Methylembelin		6.912	308.1976	C18H28O	56005-10-8	3	Carbonyl	-	-	-	-
POS00221	412.0913	39.55012	182.9911	163.2897	21.05958	50.58818	306.8562	243.9313	69.77617	42.39405	159.331	65.4766	144.9283	147.9609	0.029876	0.968394	0.981317	0.006863	N-Hexad	1-Hexadecanoylpyr	11.291	309.3016	C20H39N	70974-48-0	5	Pyrrolidi	-	-	-	-
POS00222	3.073699	1.782045	2.057174	0.675054	12.41482	2.737574	15.75661	24.07218	2.806026	6.959452	34.05468	4.561426	3.790061	14.70173	1.955693	0.068943	0.104372	0.149019	(+)-Prosoj	NP-014287	8.718	313.2602	C18H35N	14058-39-0	4	-	-	-	-	-
POS00223	664.7141	329.7525	437.6924	416.6218	156.2017	381.0704	574.4297	671.4191	553.2073	492.2889	745.3524	558.7297	397.6755	599.2379	0.591537	0.026015	0.045492	0.59098	Dehydroj	(8E)-2-Amino-8-octa	5.317	315.2759	C18H37N	3687-54-5	4	Organoni	-	-	-	-
POS00224	0.900859	0.651481	0.660326	0.511857	0.470903	0.566295	38.62891	12.47496	10.44128	29.77779	56.33421	22.55258	1	28.36829	4.826207	0.002835	0.007131	0.266689	Isorhamn	Isorhamnetin	4.424	316.0569	C16H12O	480-19-3	4	Flavonoic	-	-	-	-
POS00225	50.22739	30.53749	23.86749	22.25512	38.57965	46.61932	249.0944	170.0618	110.4915	143.9197	109.9092	124.0063	35.34774	151.2471	2.097218	0.000389	0.00161	0.518174	Cafestol	Cafestol	7.347	316.1998	C20H28O	469-83-0	12	Naphthof	-	-	-	-
POS00226	155.0916	87.84182	84.00234	82.01225	55.89659	67.03061	51.94239	61.24796	46.20841	35.05523	71.01671	48.83764	88.64587	52.38472	-0.75891	0.036754	0.061831	0.25576	Phytosph	Phytosphingosine	5.579	317.2916	C18H39N	554-62-1	4	Organoni	C12144	ko01100//	Global an	Metabolism
POS00227	35.71322	17.92977	19.55948	16.71888	29.16746	30.54199	77.04281	73.02075	43.28436	55.65137	69.64538	42.57304	24.93847	60.20295	1.271462	0.000503	0.001965	0.294559	Leukotrie	NP-020521	7.865	318.2153	C20H30O	72059-45-1	13	Fatty Acy	C00909	ko01100//	Global an	Metabolism
POS00228	26.29959	9.59832	22.07821	18.11466	7.915435	9.035421	20.0341	11.82346	20.78596	14.56201	34.0862	15.33821	15.50694	19.43832	0.32599	0.406026	0.477592	0.058414	Gallocate	N'2-{2-[(4-Chloroph	0.821	322.065	C15H14O	NA	12	-	-	-	-	-
POS00229	127.0774	154.5821	76.50956	53.69454	220.7319	76.62435	892.1337	1667.706	1204.199	943.3812	1742.446	1003.858	118.2033	1242.287	3.393656	2.75E-05	0.000274	1.718108	(-)-Phaseollin		2.72	322.115	C20H18O	13401-40-6	17	Isoflavon	-	-	-	-
POS00230	34.31856	21.7755	18.26434	13.91633	52.30661	25.58885	108.9438	67.72545	51.84129	35.30817	100.3716	38.66442	27.69503	67.14246	1.277598	0.01822	0.033662	0.287226	5'-CMP	Cytidine 5'-monoph	0.85	323.0504	C9H14N3	63-37-6	4	Pyrimidir	C00055	ko01100//	Global an	Metabolism
POS00231	2.925602	4.139113	2.000945	2.134065	15.23678	5.29137	11.33239	8.171821	7.877722	2.823414	26.97224	7.06241	5.287979	10.70667	1.017721	0.205964	0.270483	0.094048	Uridine n	Uridine 5'-monopho	0.865	324.0343	C9H13N2	58-97-9	4	Pyrimidir	C00105	ko01100//	Global an	Metabolism
POS00232	0.740435	0.542073	0.536766	0.463369	0.445286	0.51759	113.7203	118.928	71.15532	89.2305	244.1799	99.70896	1	122.8205	6.940407	0.000683	0.002351	0.572481	Mahalebc	skimmin	1.934	324.0831	C15H16O	NA	4	Coumarir	-	-	-	-
POS00233	0.811162	29.7073	31.46106	26.52253	31.2524	34.67753	7.964036	4.466561	8.199685	12.94457	9.085984	9.068344	25.73866	8.621529	-1.57792	0.00831	0.016878	0.169886	Sophorapterocarpan A		1.432	324.1303	C20H20O	NA	18	-	-	-	-	-
POS00234	0.445833	0.391805	0.339155	0.278843	0.305127	0.348597	0.324587	0.790318	0.317629	0.377363	0.518039	0.5184	1	1	0	0.14208	0.194394	0.012689	Betagarin		1.983	328.1011	C18H16O	60132-69-6	20	Flavonoic	-	-	-	-
POS00235	9698.923	6462.522	5587.387	4884.999	7995.885	4760.938	23084.52	20484.81	18269.55	11102.49	27621.6	11982.6	6565.109	18757.6	1.514584	0.001211	0.003568	5.407882	Corchorifatty acid F		4.09	328.2228	C18H32O	95341-44-9	6	Fatty Acy	-	-	-	-
POS00236	17.85931	19.97358	10.58698	11.03639	16.21084	13.32137	65.16529	61.02954	53.05127	49.92912	70.20644	47.48739	14.83141	57.81151	1.962701	8.33E-07	2.72E-05	0.341914	Gibberelli	NCGC00385965-01_	9.721	330.147	C19H22O	NA	1	-	C11867	ko01110//	Global an	Metabolism
POS00237	185.4681	94.95036	48.12586	62.09119	70.573	71.35498	198.7854	204.5934	196.104	120.5417	179.487	137.7652	88.76058	172.8795	0.961775	0.007043	0.014763	0.383131	MG(16:0/	NCGC00186665-03!2	10.339	330.2754	C19H38O	32899-41-5	4	Glyceroli	-	-	-	-
POS00238	0.643388	1.403447	0.459988	0.351187	0.657593	4.769427	234.3923	294.1115	300.9406	249.4005	468.8224	239.8384	1.380838	297.9176	7.753225	9.24E-06	0.000121	0.897322	Laricitrin		2.789	332.0491	C16H12O	53472-37-0	12	-	C12633	ko00944//	Biosynthe	Metabolism
POS00239	0.567008	0.345549	0.365211	0.295329	0.258592	0.320707	7.480155	3.360997	3.840151	3.48312	7.646721	3.743749	1	4.925815	2.300363	0.000282	0.001264	0.109221	Annulatir	Annulatin	3.071	332.0517	C16H12O	NA	4	-	-	-	-	-
POS00240	81.21217	43.19476	41.45904	40.73697	66.01137	53.00442	117.037	113.3235	107.3773	103.2236	129.831	113.1228	54.26979	113.9859	1.070634	1.46E-05	0.000171	0.387573	Gibberelli	NP-019547	7.229	332.1648	C19H24O	468-44-0	7	-	C11864	ko01110//	Global an	Environmental Information Processing;Metabolism
POS00241	144.2904	59.71275	92.89133	73.35235	101.76	105.6126	171.7784	116.93	114.3148	122.6159	122.5169	143.7678	96.2699	131.9873	0.455243	0.038363	0.063677	0.229881	Gibberelli	Andrographolide	6.098	332.1946	C20H28O	NA	12	-	C11857	ko01100//	Global an	Metabolism
POS00242	69.71802	46.29137	46.09057	38.69943	37.87215	45.40151	30.89722	32.1637	32.21357	24.78545	31.37391	34.30524	47.34551	30.95652	-0.61298	0.00754	0.015552	0.193106	Seneciphylline		1.533	333.1522	C18H23N	480-81-9	16	-	-	-	-	-
POS00243	277.292	46.49347	116.6416	74.34459	1.950197	13.65417	0.336782	0.30213	0.281941	0.281525	0.355242	0.302534	88.39601	1	-6.46591	0.059403	0.091197	0.404109	Junosidin	Junosidine	7.053	337.13	C20H19N	110883-39-	4	Quinoline	-	-	-	-
POS00244	9434.263	2558.635	5493.694	5098.951	1130.674	2203.694	8097.836	7504.326	6344.492	3745.866	8001.884	3089.345	4319.985	6130.625	0.505008	0.263713	0.331669	1.252268	13Z-Doco	Erucamide	12.795	337.3324	C22H43N	112-84-5	6	-	-	-	-	-
POS00245	11.64648	12.76765	3.999159	0.273088	4.87744	4.999737	38.15273	41.79877	27.16154	35.84714	32.92919	47.49682	6.427259	37.23103	2.53423	4.88E-06	7.60E-05	0.279242	4-p-Coun	NP-000587	2.313	338.0985	C16H18O	93451-44-6	4	-	-	-	-	-
POS00246	18.46823	12.56783	16.02468	16.12713	7.565654	10.04362	12.59661	10.88402	10.664	12.94277	16.08463	7.463723	13.46619	11.77263	-0.19391	0.430005	0.497741	0.031063	Evoprenine		2.397	339.1512	C20H21N	16584-45-5	12	-	-	-	-	-
POS00247	4.053857	3.322026	2.838055	1.824059	2.197873	1.863076	2078.682	1237.54	558.6759	1067.858	2331.218	998.6538	2.683158	1378.771	9.005236	0.00059	0.0022	1.919113	Sinapoyl	Sinapoyl malate	2.699	340.0774	C15H16O	NA	5	-	C02887	ko00940//	Biosynthe	Metabolism
POS00248	615.6365	498.6314	331.3792	312.9111	221.3746	450.0117	177.8535	145.6177	201.8827	128.1201	221.7555	162.5707	404.9908	172.9667	-1.22739	0.003205	0.007822	0.725631	Cassythine		0.854	341.1306	C19H19N	4030-51-7	12	-	-	-	-	-
POS00249	1930.84	1320.112	1265.755	1262.326	935.5877	1118.105	2481.705	2689.285	2070.014	2175.973	3700.084	2616.771	1305.454	2622.305	1.006284	0.000722	0.00246	1.81562	Trehalose	Trehalose	0.817	342.1144	C12H22O	99-20-7	5	Organoox	C01083	ko01100//	Carbohyc	Environmental Information Processing;Metabolism
POS00250	0.72414	0.499664	0.517708	0.441996	0.387124	0.489832	1.913031	0.699582	0.653506	2.664175	18.37624	2.514843	1	4.47023	2.160349	0.188194	0.251181	0.08388	DIBOA-g	DIBOA-glucoside	2.378	343.089	C14H17N	155835-54-	3	Organoox	C15772	ko00402//	Biosynthe	Metabolism
POS00251	44.57536	43.55435	23.14563	19.55678	60.70304	42.99616	22.56915	60.76536	62.72184	20.79779	42.55186	11.16427	39.08855	36.76171	-0.08854	0.835226	0.867044	0.034113	cGMP		1.248	345.0456	C10H12N	7665-99-8	5	Purine nu	C00942	ko00230//	Nucleotid	Metabolism
POS00252	0.652598	0.46869	0.48154	0.383489	0.322993	0.424926	5.532746	2.039399	2.687336	2.188179	5.459914	2.746671	1	3.442374	1.783404	0.001109	0.003308	0.086463	Tomentin	3',4',5,6-tetrahydroxy	3.858	346.0673	C17H14O	NA	4	-	-	-	-	-
POS00253	166.3219	98.70559	102.4351	105.1188	77.60988	98.16243	139.7272	154.9055	121.6534	109.2672	141.5749	119.052	108.0589	131.03	0.278079	0.135645	0.18824	0.158954	9-(3,4-Dir	9-(3,4-Dimethoxyph	7.685	346.1213	C22H18O	NA	2	Naphthal	-	-	-	-
POS00254	1145.579	779.3138	690.9032	623.5604	959.8805	591.7275	2776.176	2423.72	2147.24	1343.262	3127.155	1415.629	798.494	2205.531	1.465772	0.001003	0.003054	1.839504	Ginkgoic acid		4.088	346.2529	C22H34O	22910-60-7	6	Benzene a	-	-	-	-
POS00255	26.01395	18.44845	16.87826	14.49149	33.6251	12.09578	12.77258	13.39853	3.973176	8.128328	71.31505	4.504517	20.25884	19.01536	-0.09139	0.912917	0.938755	0.021797	Adenosin	Adenosine 5'-monop	0.853	347.0614	C10H14N	61-19-8	4	Organoox	C00020	ko01100//	Global an	Metabolism
POS00256	1150.986	2542.298	661.4782	368.6856	197.9281	3241.315	2.320129	12.10302	1.92354	3.036959	1.010352	0.738997	1360.448	3.522166	-8.5934	0.023866	0.042646	1.535388	Camptothecin		6.954	348.1064	C20H16N	2114454	13	-	-	-	-	-
POS00257	1794.389	1288.219	1137.93	1027.396	1439.57	953.4037	3348.111	3257.267	2833.09	1879.853	4111.983	2194.787	1273.484	2937.515	1.205815	0.000883	0.002827	2.003354	Oryzalic a	(11E,15Z)-9,10,13-tri	4.094	350.205	C20H30O	134859-97-	12	Oxanes	-	-	-	-
POS00258	44.69574	34.34791	30.90581	30.92738	60.67236	31.50561	45.53439	37.48635	39.98112	37.177	55.05175	40.94269	38.84247	42.69555	0.136451	0.506722	0.565523	0.053686	trans-Zea	trans-Zeatin-ribosid	2.023	351.1527	C15H21N	6025-53-2	4	Purine nu	C16431	ko01110//	Global an	Metabolism
POS00259	3.957573	2.841432	3.602247	0.268829	9.212223	2.248353	146.5572	179.6136	96.16606	124.0504	209.3275	158.1374	3.688443	152.3087	5.367843	3.87E-06	7.03E-05	0.644349	Citrunobi	1-(alpha-D-Glucopy	2.793	352.1256	C21H20O	126026-23-	16	Linear 1,3	-	-	-	-
POS00260	234.1274	113.5583	85.21984	78.41435	144.3208	74.27671	386.46	324.3147	260.7993	145.4663	448.9549	126.8512	121.6529	282.1411	1.213646	0.02059	0.037404	0.559955	PGE2	(12Z)-9,10,11-trihyd	4.405	352.2207	C20H32O	363-24-6	12	Fatty Acy	C00584	ko01100//	Global an	Metabolism
POS00261	0.498345	0.351026	0.369355	0.410483	0.242194	0.312861	65.32649	31.56688	8.081211	48.18711	33.68438	62.94588	1	41.63199	5.379621	0.000889	0.002827	0.31465	Chloroge	(1r,3R,4s,5S)-4-{[(2E)	2.079	354.0937	C16H18O	327-97-9	3	Organoox	C00852	ko01110//	Biosynthe	Metabolism
POS00262	0.725747	1.657581	1.526029	0.416867	0.366727	3.70169	34.19171	38.77761	27.09275	37.69217	63.25276	26.0357	1.399107	37.84045	4.757351	6.31E-05	0.000439	0.316804	Asarinin		2.603	354.1087	C20H18O	13079-95-3	4	-	-	-	-	-
POS00263	1.98097	0.901442	0.363389	0.407901	0.337158	0.376166	3073.812	1819.188	797.2296	1770.136	3539.482	1419.758	1	2069.934	11.01537	0.000629	0.002219	2.354412	HMBOA-Glc		2.74	357.1041	C15H19N	17622-26-3	5	Benzoxaz	-	-	-	-
POS00264	42.6205	63.75586	22.14592	21.8514	92.40828	61.91591	42.81261	100.0225	69.5875	33.11701	97.38226	25.58871	50.78298	61.41843	0.274327	0.55355	0.613596	0.087386	guanosin	Guanosine 5'-monop	1.224	363.0562	C10H14N	85-32-5	4	Purine nu	C00144	ko01100//	Global an	Metabolism
POS00265	246.7079	188.721	112.497	102.3574	365.318	184.5831	494.11	506.4926	426.3623	291.8451	504.5502	287.1227	200.0307	418.4138	1.064709	0.003733	0.008845	0.682682	Terminali	Terminaline	6.477	363.312	C23H41N	15112-49-9	4	-	-	-	-	-
POS00266	0.571622	0.346609	0.382558	0.312666	0.257158	0.793153	11.00215	35.3651	14.26632	13.84915	19.94479	15.00386	1	18.23856	4.18892	0.000615	0.002219	0.204843	3-Feruloy	(1S,3R,4R,5R)-1,3,4-t	2.16	368.109	C17H20O	62929-69-5	4	Alcohols a	-	-	-	-
POS00267	0.879079	0.598518	0.721919	0.498553	0.451404	0.53519	106.5374	78.15494	44.46136	75.35119	106.1044	47.55125	1	76.36008	6.254747	4.35E-05	0.000365	0.45492	Tangeritii	3-(2,4-Dihydroxyph	2.631	372.1193	C20H20O	481-53-8	4	Flavonoic	-	-	-	-
POS00268	35.70748	63.04163	19.92303	13.13153	6.036272	80.64269	0.386019	0.372793	0.335197	0.361065	0.424276	0.389696	36.41377	1	-5.18641	0.013934	0.02649	0.259493	Riboflavin		6.951	376.1378	C17H20N	83-88-5	1	Flavin nu	C00255	ko01100//	Global an	Metabolism
POS00269	63.68392	46.42072	38.21574	39.61864	28.82789	42.41704	83.72075	66.78548	60.48497	63.70491	85.21844	66.90744	43.19733	71.137	0.719658	0.001441	0.004133	0.25311	Symphyti	symphytine	0.781	381.2133	C20H31N	22571-95-5	4	-	-	-	-	-
POS00270	19.89834	13.45591	10.64122	9.079518	16.90573	8.983145	37.35873	37.94356	28.87447	16.27766	50.97929	11.79243	13.16064	30.53769	1.214361	0.019631	0.036063	0.188254	Glicoisoflavanone		4.414	384.1579	C22H24O	NA	1	Flavonoic	-	-	-	-
POS00271	1091.297	521.7132	747.421	673.7706	389.4303	501.7036	310.511	231.6586	325.2264	343.0026	392.4694	295.388	654.2227	316.376	-1.04814	0.008801	0.017655	0.849436	Corchoio	(4S)-4-hydroxy-3,5,5	2.27	386.1922	C19H30O	NA	4	Fatty Acy	-	-	-	-
POS00272	6.771646	2.098769	5.271579	2.278968	2.786505	3.052628	225.0035	440.2954	473.9147	130.3977	277.8754	270.8287	3.710016	303.0526	6.351999	0.000227	0.001123	0.810703	Glucoconringiin		2.276	391.0555	C11H21N	28463-28-7	13	Carbohyc	-	-	-	-
POS00273	95.83244	80.17802	68.12084	56.38753	59.63713	70.86626	61.57519	41.82275	48.92498	42.24986	48.74606	50.5643	71.83704	48.98052	-0.55252	0.006071	0.013498	0.226218	Garciman	5,7-dihydroxy-2-(4-r	2.871	392.1066	C19H20O	NA	10	Organoox	-	-	-	-
POS00274	225.3264	148.9806	149.3729	145.762	139.4966	224.2068	170.5531	224.9535	201.2436	267.9244	265.0401	248.0866	172.1909	229.6335	0.415325	0.031006	0.053084	0.314787	3-Hydrox	(2E,4E,6E,8E,10E,12I	9.515	392.2696	C27H36O	79709-31-2	4	Prenol lip	-	-	-	-
POS00275	0.556831	0.379968	0.660188	0.317339	0.370119	0.360994	5.782262	1.243885	0.721858	0.658605	0.992341	0.59982	1	1.666462	0.736788	0.170982	0.231038	0.03889	5-Hydroxyflavone		3.277	400.1137	C21H20O	71802-04-5	5	Flavonoic	-	-	-	-
POS00276	93.6987	50.64529	72.40994	76.99466	24.26988	44.06158	9.078232	7.074012	15.30773	12.49428	5.962429	5.684476	60.34667	9.26686	-2.70312	0.000631	0.002219	0.353601	Corchoionoside B		0.802	400.1681	C19H28O	189344-54-	13	Fatty Acy	-	-	-	-
POS00277	10.99233	8.341231	5.974039	5.55538	2.758229	8.954092	72.92306	47.95139	71.25094	81.02979	61.61746	70.19172	7.095884	67.49406	3.249706	1.89E-07	1.02E-05	0.393465	Deoxygomisin A		1.567	400.194	C23H28O	82467-52-5	13	-	-	-	-	-
POS00278	1.684451	1.174169	1.26467	1.020098	1.502247	1.1806	47.22816	87.50932	38.40009	11.87756	32.23913	15.93657	1.304372	38.86514	4.897049	0.007164	0.014922	0.271916	Shoyuflavone B		2.362	402.0602	C19H14O	190712-88-	3	Isoflavon	-	-	-	-
POS00279	6.712752	4.906849	5.318857	3.925822	3.585079	4.353471	5.203536	3.385249	5.357517	3.249228	5.130809	4.732048	4.800472	4.509731	-0.09013	0.639454	0.690103	0.014949	Cassaidin	Cassaidine	2.096	407.3017	C24H41N	26296-41-3	4	-	-	-	-	-
POS00280	1.626739	1.170467	1.377667	0.904827	1.215606	0.981937	1.067893	3.089953	1.493881	2.507025	1.518798	0.791872	1.212874	1.744904	0.524718	0.186401	0.249808	0.022927	Kuwanol D		9.6	408.1945	C25H28O	123702-93-	2	Linear 1,3	-	-	-	-
POS00281	123.2311	1.898482	8.244919	9.498611	20.33778	2.529252	4.626628	4.862392	3.148835	9.615446	96.00842	52.01781	27.62335	28.37992	0.038982	0.976248	0.981317	0.00783	Stigmasterol		13.225	412.3681	C29H48O	83-48-7	5	Steroids a	C05442	ko01110//	Global an	Metabolism
POS00282	16.44149	12.05224	13.01138	10.35798	9.52957	12.11113	11.35362	11.84701	11.75972	8.463191	10.22189	11.63031	12.25063	10.87929	-0.17127	0.250762	0.321566	0.044817	Asperuloside		8.691	414.1215	C18H22O	14259-45-1	12	-	C09769	ko01110//	Global an	Metabolism
POS00283	2161.844	199.9871	89.46173	77.44797	86.08569	148.6674	89.12853	80.74736	76.28621	73.04112	138.3391	87.54978	460.5824	90.84869	-2.34192	0.303614	0.371841	0.695839	Magnosh	Bis(4-ethylbenzylide	6.51	414.2024	C24H30O	86702-02-5	4	-	-	-	-	-
POS00284	3.84994	2.294184	3.27033	2.546689	1.292611	2.708083	3.044312	1.979259	2.15526	2.55002	2.998389	1.813817	2.660306	2.42351	-0.13449	0.58135	0.640072	0.010576	Desacetylvindoline		8.282	414.2181	C23H30N	3633-92-9	6	-	C01091	ko01110//	Biosynthe	Metabolism
POS00285	161.2611	62.33489	111.1463	116.2064	58.2577	51.77152	24.88246	19.73	20.01249	28.61524	28.63312	18.44602	93.49634	23.38656	-1.99923	0.002756	0.007042	0.398436	Ancistrotectorine		2.546	421.2293	C26H31N	98985-59-2	9	-	-	-	-	-
POS00286	30.21538	39.9152	22.89191	25.90235	22.8173	28.16118	25.82321	37.12809	29.17018	27.67068	38.66947	38.90909	28.31722	32.89512	0.216194	0.228611	0.295477	0.073293	Nigakilac	(S)-AL 8810	9.59	424.2052	C22H32O	30248-06-7	10	-	-	-	-	-
POS00287	33.07183	49.31298	20.65075	16.82667	5.812567	76.25255	0.542833	2.141004	1.571369	2.835075	0.445423	0.594484	33.65456	1.355031	-4.6344	0.011578	0.022271	0.247661	Mangostanol		3.586	426.1635	C24H26O	184587-72-	10	Benzopyr	-	-	-	-
POS00288	1.347517	0.808209	1.189736	0.813007	1.342078	0.810089	0.781992	4.581455	4.711176	1.355711	0.866782	4.496547	1.051773	2.798944	1.412059	0.057871	0.090113	0.043756	Marchantin A		2.77	440.1634	C28H24O	88418-46-6	2	-	-	-	-	-
POS00289	51.27787	31.37878	33.13191	26.80601	22.35763	34.10964	122.0609	148.231	117.9874	91.82938	173.4956	96.86209	33.17697	125.0777	1.914571	4.22E-05	0.000363	0.490315	Tetrahydrofolic acid		0.783	445.1773	C19H23N	135-16-0	14	Pteridine	C00101	ko01100//	Amino ac	Genetic Information Processing;Metabolism
POS00290	0.609018	0.41558	0.458454	0.347081	0.32479	0.394827	5.388667	0.560424	0.445032	0.578852	0.52472	0.433125	1	1.321803	0.402508	0.296819	0.366263	0.02924	Swertisin	Swertisin	3.279	446.1193	C22H22O	6991/10/2	4	-	-	-	-	-
POS00291	1183.905	2146.174	1572.345	1607.668	1281.928	1904.741	2043.712	688.125	630.632	730.914	1245.074	627.1464	1616.127	994.2672	-0.70084	0.046876	0.074773	0.883932	Stigmastentriol		12.283	446.3738	C29H50O	NA	4	Steroids a	-	-	-	-
POS00292	13.68321	7.281766	11.95409	10.7725	11.74355	7.832368	77.33144	94.24223	53.29168	47.34531	79.34744	51.87792	10.54458	67.23934	2.672804	2.76E-05	0.000274	0.38625	Luteolin 7	Cynaroside	2.051	448.0979	C21H20O	1268798	5	Flavonoic	C03951	ko00944//	Biosynthe	Metabolism
POS00293	3.669772	7.201156	5.634296	7.138928	7.884416	8.439352	135.6722	98.38252	90.91371	31.78811	86.8224	59.65088	6.66132	83.87164	3.654303	0.000327	0.00139	0.428721	Trifolin	Trifolin	2.367	448.0983	C21H20O	23627-87-4	4	Flavonoic	C12626	ko00944//	Biosynthe	Metabolism
POS00294	1.753235	1.220735	1.124587	0.941786	1.18076	0.948676	2.622696	2.285269	2.122713	1.401992	3.071414	1.561826	1.194963	2.177652	0.865807	0.006231	0.013675	0.045999	Acuminoside		4.088	448.2234	C21H36O	120163-17-	16	Prenol lip	-	-	-	-
POS00295	41.69418	19.42067	29.97254	25.45452	17.26146	19.53928	291.3708	206.5041	178.6317	168.2295	283.5552	162.3571	25.55711	215.1081	3.073265	1.33E-05	0.000161	0.715055	Cyanidin 3-galactoside		2.71	449.1013	C21H21O	27661-36-5	15	Flavonoic	-	-	-	-
POS00296	170.808	86.5561	52.22203	60.9695	50.36657	32.79757	71.53988	79.71008	68.54997	103.2111	175.9838	65.93345	75.61996	94.15471	0.316266	0.503153	0.563462	0.112269	Phylloqui	Reduced Vitamin K	12.21	450.3478	C31H46O	84-80-0	4	Prenol lip	C02059	ko01100//	Global an	Metabolism
POS00297	0.590486	0.725873	0.568515	0.357748	1.478112	1.06747	4.529966	3.570476	1.740136	1.216018	2.077983	1.229907	1	2.394081	1.259472	0.020198	0.036898	0.055019	LysoPE(0	1-palmitoyl-2-hydro	7.332	453.2829	C21H44N	NA	5	Glyceropl	-	-	-	-
POS00298	5.773572	4.027635	6.419956	5.531613	2.1608	4.057321	0.282652	0.317776	1.095344	0.312412	0.360826	0.271165	4.661816	1	-2.22089	6.83E-05	0.000447	0.10349	Gein		2.225	458.1742	C21H30O	585-90-0	10	Organoox	-	-	-	-
POS00299	0.668226	0.688693	0.482157	0.403888	6.593496	0.688959	0.500336	0.492122	0.457965	0.402854	42.63038	0.458896	1.58757	7.490426	2.238228	0.425106	0.4962	0.091871	Adenylos	Adenylsuccinic acid	1.505	463.0723	C14H18N	19046-78-7	3	Purine nu	C03794	ko01100//	Amino ac	Metabolism
POS00300	0.460966	0.303994	0.327264	0.268493	0.22582	0.264148	250.7214	119.5228	95.9855	256.1268	185.7307	210.6364	1	186.4539	7.542675	4.53E-05	0.000367	0.695295	Quercetir	Quercetin-3β-D-gluc	2.546	464.0936	C21H20O	482-35-9	4	Flavonoic	C05623	ko01100//	Biosynthe	Metabolism
POS00301	0.846033	0.618699	0.539533	0.55819	0.4605	0.549793	0.608455	0.596652	0.566843	0.492007	0.722427	0.57169	1	1	0	0.969469	0.981317	0.00055	Panaxatriol		13.07	476.3842	C30H52O	NA	4	Prenol lip	-	-	-	-
POS00302	320.253	66.07368	219.0966	46.02355	25.18829	62.14848	5584.487	2934.859	4844.337	5684.722	5572.262	5751.506	123.1306	5062.029	5.361455	6.55E-07	2.68E-05	3.611	Isorhamn	5,7-dihydroxy-2-(4-h	2.744	478.1087	C22H22O	NA	5	Flavonoic	-	-	-	-
POS00303	3.979244	4.596515	0.941865	0.870584	26.04518	1.596272	9.268836	6.186514	6.51065	2.935415	10.22002	2.160816	6.338277	6.213709	-0.02864	0.976964	0.981317	0.002216	LysoPE(18:0/0:0)		8.836	481.3145	C23H48N	69747-55-3	4	Glyceropl	-	-	-	-
POS00304	40.25433	25.53038	24.04514	23.44276	41.95604	25.06774	95.38379	47.82806	52.6894	43.09308	66.83045	30.64849	30.0494	56.07888	0.900121	0.024923	0.043817	0.219048	PC(16:0/0	PC(16:0/0:0)	7.622	495.33	C24H50N	NA	5	Glyceropl	-	-	-	-
POS00305	1.565075	0.560659	0.379651	0.30177	0.273065	0.330731	3.498245	4.06454	3.581438	2.262692	5.537726	0.406105	1	3.225124	1.689355	0.004878	0.011001	0.076211	Eriojapos	eriojaposide A	2.088	502.2402	C24H38O	290308-51-	2	Fatty Acy	-	-	-	-

POS00306	0.412677	0.312348	0.33188	0.269741	0.338158	0.323557	0.498678	1.051551	1.178368	0.708708	1.012502	0.29473	1	1	0	0.009416	0.018661	0.029042	Mycalam	(2R)-3-{[(2-Aminoeth	7.559	503.2986	C24H41N	115185-92-	4	-	-	-	-	-
POS00307	17.04865	10.02859	6.251783	7.336502	21.69774	5.099452	106.0482	86.3763	74.60076	21.8856	122.332	29.06492	11.24379	73.38464	2.70635	0.004123	0.009562	0.37725	Protobass	(1α,2α,3β,5ξ,9ξ,18ξ)	4.088	504.3427	C30H48O	37905-13-8	4	Prenol lip	-	-	-	-
POS00308	10.50964	9.214663	6.714518	6.106799	17.34728	7.059409	18.15422	11.73702	12.27164	11.84211	15.97261	10.11499	9.492052	13.34877	0.491915	0.098403	0.141131	0.075372	LysoPC(1	1-Stearoyl-sn-glycer	8.907	523.3613	C26H54N	19420-57-6	4	Glyceropl	C04230	ko00564//	Lipid met	Metabolism
POS00309	1.09343	0.743178	0.731822	0.707306	0.581868	0.670773	0.773083	0.680459	0.669265	0.568437	0.842007	0.621223	1	1	0	0.467844	0.531198	0.006739	Diosbulbinoside F		9.489	538.1993	C26H34O	66756-58-9	10	Prenol lip	-	-	-	-
POS00310	31.4964	27.426	24.06964	22.39117	19.33992	17.7775	277.0355	197.1968	124.8378	96.4246	295.3213	131.5312	23.7501	187.0579	2.977479	0.000761	0.00254	0.649083	Isochama	(1S,4aS,7aS)-7-({[(2E	0.797	542.1224	C30H22O	NA	2	-	-	-	-	-
POS00311	408.4234	386.4677	337.5129	399.8241	316.7454	385.8055	371.7943	532.0009	589.059	476.3401	560.1984	591.6601	372.4631	520.1755	0.481901	0.002798	0.007093	0.545007	Echineno	(13'cis,15cis)-2',3',4',5	12.657	550.4149	C40H54O	NA	4	-	C08592	ko01110//	Global an	Metabolism
POS00312	524.1001	210.7017	184.6452	151.73	422.8029	268.0478	2020.897	1446.67	1124.52	1064.449	1893.853	838.3419	293.6713	1398.122	2.251216	0.000292	0.001291	1.671325	Pheophor	Pheophorbide A	11.126	592.2649	C35H36N	15664-29-6	6	-	C18021	ko01110//	Global an	Metabolism
POS00313	413.6247	238.8792	238.773	281.8683	139.9181	214.0325	483.3767	291.7267	318.8828	307.3091	366.7865	388.1348	254.516	359.3694	0.497712	0.05011	0.079159	0.392499	Auroxant	2-Acetamido-4-(5-ar	11.576	600.4148	C40H56O	27785-15-5	5	Prenol lip	-	-	-	-
POS00314	216.5543	105.5662	100.2072	85.01778	188.4361	144.4975	561.6225	375.7394	349.5969	295.7079	630.1414	290.7698	140.0465	417.263	1.575051	0.001266	0.003663	0.82521	Phaeophorbide b		9.766	606.244	C35H34N	20239-99-0	6	Tetrapyrr	-	-	-	-
POS00315	233.1564	188.569	86.60123	143.857	212.4283	227.8823	767.0187	993.3702	689.3184	557.9559	1110.047	550.3944	182.0824	778.0174	2.095211	0.000107	0.000637	1.25011	Cepharan	(1S,2S,3aR,4S,5S,9R,	12.419	606.2804	C37H38N	481-49-2	12	-	-	-	-	-
POS00316	245.2418	116.7165	224.3851	219.039	101.1834	103.2517	207.2962	491.3179	232.0233	220.4965	534.6513	249.3581	168.3029	322.5239	0.938346	0.043533	0.070472	0.5451	Rutin	NP-008131	2.014	610.1496	C27H30O	153-18-4	6	Flavonoic	C05625	ko01100//	Biosynthe	Metabolism
POS00317	18.87419	7.981249	9.305362	8.152813	12.6743	33.55774	335.807	306.1121	271.7911	470.1813	288.9618	182.049	15.09094	309.1504	4.356554	1.87E-05	0.000211	0.861074	Cyanidin	3-glucogalactoside	2.364	611.153	C27H31O	142562-01-	13	Flavonoic	-	-	-	-
POS00318	34.13663	38.45629	9.240112	17.05809	25.42369	31.64784	252.8423	229.3923	347.7896	217.1361	295.9582	203.8342	25.99378	257.8255	3.310157	1.35E-06	3.40E-05	0.76639	Glutathio	Epigallocatechin	0.86	612.1489	C20H32N	27025-41-8	5	Flavonoic	C12136	ko01110//	Biosynthe	Metabolism
POS00319	61.23578	32.38861	36.60869	34.72423	81.90498	49.04297	30.81126	24.23178	46.83434	14.00441	23.90342	16.7204	49.31754	26.08427	-0.91892	0.030641	0.052734	0.213916	Kanokoside D		10.391	624.2551	C27H44O	64703-88-4	12	Prenol lip	-	-	-	-
POS00320	0.589498	0.28641	0.272166	0.268376	0.260881	0.27817	46.3816	58.87296	34.54469	100.8124	69.34733	92.44574	1	67.06746	6.067541	8.63E-05	0.000543	0.411229	Quercetin	NCGC00385380-02!	1.913	626.1447	C27H30O	42900-82-3	5	Flavonoic	-	-	-	-
POS00321	6.817182	3.636357	2.598566	2.321796	5.860211	0.962366	43.62523	28.09715	24.55887	9.152912	40.66983	9.783724	3.699413	25.98129	2.812105	0.004297	0.009825	0.224748	Ginsenoside Rh1		4.088	638.4356	C36H62O	63223-86-9	5	Prenol lip	-	-	-	-
POS00322	1.375682	0.489998	1.22433	0.873806	0.258824	1.010555	39.24405	11.0455	35.12296	19.79816	13.36233	11.41545	1	21.66474	4.437277	0.002235	0.005941	0.200355	Rhamneti	3-{[(2S,3R,4S,5S,6R)-	2.276	640.1598	C28H32O	259234-17-	6	Flavonoic	-	-	-	-
POS00323	167.0083	107.1296	123.2664	117.4913	139.714	143.0205	136.004	137.6904	118.3112	131.9106	176.5777	149.4598	132.9383	141.659	0.091665	0.48181	0.543283	0.087643	Pittoside	2-[5-[2-[2-[5-[2-[2-[5-	12.519	768.4732	C41H68O	75303-44-5	9	-	-	-	-	-
POS00324	0.51147	0.359769	0.358201	0.298078	0.255776	0.31296	121.1887	60.31629	34.50587	69.97777	141.0501	76.61033	1	83.94151	6.391312	0.000431	0.001717	0.474646	Cyanidin	3-glucoside 5-caffeoy	2.429	772.18	C36H36O	-	6	-	C16372	ko00942//	Biosynthe	Metabolism
POS00325	200.1007	146.9042	149.8563	153.4018	119.1432	246.243	525.7871	824.1286	566.2722	309.9272	879.688	316.274	169.2749	570.3462	1.75247	0.00261	0.006721	0.970632	Quercetin	[Similar to: NP-0081	1.789	772.2009	C33H40O	50867-29-3	6	Flavonoic	-	-	-	-
POS00326	0.536098	0.383079	0.407005	0.333914	0.272916	0.34352	62.87643	40.30318	38.78703	32.99397	63.44936	46.19924	1	47.43487	5.567876	4.38E-06	7.42E-05	0.359156	Kaempferol	3-sophoroside 7-g	2.576	786.1947	C33H38O	140163-56-	11	-	-	-	-	-
POS00327	0.509155	0.307551	0.344268	0.277543	0.241348	0.295539	71.24865	88.87151	75.71025	94.07227	120.9546	85.2026	1	89.34331	6.481288	2.19E-07	1.02E-05	0.497647	Moracetin	NCGC00179992-02!	1.738	788.1961	C33H40O	61474-16-6	6	Flavonoic	-	-	-	-
