# Supplementary material for: BcMYB111 Responds to BcCBF2 and Induces Flavonol Biosynthesis to Enhance Tolerance under Cold Stress in Non-Heading Chinese Cabbage
Source: Int J Mol Sci. 2023 May 12;24(10):8670. doi: 10.3390/ijms24108670 (PMC10217907; doi:10.3390/ijms24108670)
Supplement: Supplementary file 1 [file ijms-24-08670-s001.zip › Supplementary Table S2.pdf]

Supplementary Table S2 -List of primers used for the gene cloning, vector constructions and qRT-PCR analyses.

**Name**	**Primer sequence (5'-3')**
BcCBF2-F	ATGACCTCATTTTCTACCTTTTCTG
BcCBF2-R	CTAATAACTCCAAAGGGACACGTC
BcCBF2-AD-F	gtaccagattacgctcatatgATGACCTCATTTTC
BcCBF2-AD-R	atgcccacccgggtggaattcATAACTCCAA
BcCBF2-RT-F	ATGGAGGAGACGATCGTGGA
BcCBF2-RT-R	ACAAGGCCGGCATCTCTAAC
BcCHS-RT-F	CTGCGCTTATCGTTGGTTCG
BcCHS-RT-R	CCGAGTCGGGAAGAATGGTC
BcF3H-RT-F	CGAGCAGACTATCAATAG
BcF3H-RT-R	ATATCTCTACTCATCTTTCTC
BcFLS1-RT-F	CATCTTCCCTCCACACCGAAG
BcFLS1-RT-R	TCTTCGTCGGGATCGCTCA
BcMYB111-RT-F	GCCTCACAATGTCTCCCACA
BcMYB111-RT-R	TACGCTCCCAACGACTTTCC
AtF3H-RT-F	CCGTCAGATCGTTGAGGCTT
AtF3H-RT-R	AATCTTGCACAGCCTCTCCC
AtFLS1-RT-R	AAGCGAGTGAAGAATGGGGG
AtFLS1-RT-R	CCGGTTTAGCGACGGATTCT
AtACTIN-RT-F	CCATTGCTTAATTCCACGGACAAAC
AtACTIN-RT-R	TTGACAATTGATGCAAACAATGACG
BcGAPC-RT-F	AGAGCCGCTTCCTTCAACATCATT
BcGAPC-RT-R	TGGGCACACGGAAGGACATACC
BcCBF2-pRI101-F	tcttcactgttgatacatatgATGACCTCATTTTC
BcCBF2-pRI101-R	gcccttgctcaccatggatccATAACTCCA
BcMYB111-pRI101-F	tcttcactgttgatacatatgATGGGAAGGGC
BcMYB111-pRI101-R	gcccttgctcaccatggatccTATGCTATCCA
BcMYB111-pGreenII-0800-F	ctatagggcgaattgggtaccATATCCAGTA
BcMYB111-pGreenII-0800-R	atcgataccgtcgacctcgagCTCTTCTCTC
BcFLS1-pGreenII-0800-F	ctatagggcgaattgggtaccGAACTTACTG
BcFLS1-pGreenII-0800-R	atcgataccgtcgacctcgagAGCTTTAGTTA
BcF3H-pGreenII-0800-F	ctatagggcgaattgggtaccGGTTGTTATTG
BcF3H-pGreenII-0800-R	atcgataccgtcgacctcgagTTATAATTAT
pClod-HIS-BcCBF2-F	tcggtaccctcgagggatccATGACCTCAT
pClod-HIS-BcCBF2-R	aggtcgacaagcttgaattcCTAATAACTC
BcMYB111-Pro-F	ATATCCAGTAAAATAGTAATATAAT
BcMYB111-Pro-R	CATCTCTTCTCTCAAAAAAGAGAA
BcMYB111-F	ATGGGAAGGGCTCCTTGTTGTGAGA
BcMYB111-R	TCATATGCTATCCACTTGCTGTATT
BcMYB111-pCAMBIA1302-F	acgggggacgagctcggtaccATGGGAAGGGC
BcMYB111-pCAMBIA1302-R	tcctttactgtcgactctagaTATGCTATCCA
BcMYB111-DRE-pAbAi	cttgaattcgagctcggtaccCCACATAAACGTCTTCATATAAACATAGCCATTCTCGCCGACTCTACTAGCCATAGAAACATAGCCATTCTCGCCGACTCTACTAGCCATAGAAACATAGCCATTCTCGCCGACTCTACTAGCCATAGACGTGTTTGTTCCATTGACTCTctcgaggcatgtgctctgtat
BcMYB111-DRE-m-pAbAi	cttgaattcgagctcggtaccCCACATAAACGTCTTCATATAAACATAGCCATTCTCCCCCACTCTACTAGCCATAGAAACATAGCCATTCTCCCCCACTCTACTAGCCATAGAAACATAGCCATTCTCCCCCACTCTACTAGCCATAGACGTGTTTGTTCCATTGACTCTctcgaggcatgtgctctgtat
BcMYB111-pJG-F	gattatgcctctcccgaattcATGGGAAGGGC
BcMYB111-pJG-R	agaagtccaaagcttctcgagTATGCTATCCA
BcCHS-pLaczi-F	tttgatattggatcggaattcCTTAGTTGTTGCGACTTAGTTGTTGCGACTTAGTTGTTGCGAatacagagcacatgcctcgag
BcCHS-pLaczi-R	ctcgaggcatgtgctctgtatTCGCAACAACTAAGTCGCAACAACTAAGTCGCAACAACTAAGgaattccgatccaatatcaaa
BcCHS-m-pLaczi-F	tttgatattggatcggaattcCTTAAAAAAAGCGACTTAAAAAAAGCGACTTAAAAAAAGCGAatacagagcacatgcctcgag
BcCHS-m-pLaczi-R	ctcgaggcatgtgctctgtatTCGCTTTTTTTAAGTCGCTTTTTTTAAGTCGCTTTTTTTAAGgaattccgatccaatatcaaa
BcF3H-pLaczi-F	tttgatattggatcggaattcGGTCCCAGGTAGCTGGTCCCAGGTAGCTGGTCCCAGGTAGCTatacagagcacatgcctcgag
BcF3H-pLaczi-R	ctcgaggcatgtgctctgtatAGCTACCTGGGACCAGCTACCTGGGACCAGCTACCTGGGACCgaattccgatccaatatcaaa
BcF3H-m-pLaczi-F	tttgatattggatcggaattcGGCCCCCCCTAGCTGGCCCCCCCTAGCTGGCCCCCCCTAGCTatacagagcacatgcctcgag
BcF3H-m-pLaczi-R	ctcgaggcatgtgctctgtatAGCTAGGGGGGGCCAGCTAGGGGGGGCCAGCTAGGGGGGGCCgaattccgatccaatatcaaa
BcFLS1-pLaczi-F	tttgatattggatcggaattcTGTGGTAGTTACTTTGTGGTAGTTACTTTGTGGTAGTTACTTatacagagcacatgcctcgag
BcFLS1-pLaczi-R	ctcgaggcatgtgctctgtatAAGTAACTACCACAAAGTAACTACCACAAAGTAACTACCACAgaattccgatccaatatcaaa
BcFLS1-m-pLaczi-F	tttgatattggatcggaattcTGTAAAAAAAACTTTGTAAAAAAAACTTTGTAAAAAAAACTTatacagagcacatgcctcgag
BcFLS1-m-pLaczi-R	ctcgaggcatgtgctctgtatAAGTTTTTTTTACAAAGTTTTTTTTACAAAGTTTTTTTTACAgaattccgatccaatatcaaa
BcMYB111-EMSA-F	CATTCTCGCCGACTCTACTACATTCTCGCCGACTCTACTA
BcMYB111-EMSA-R	TAGTAGAGTCGGCGAGAATGTAGTAGAGTCGGCGAGAATG
BcMYB111-m-EMSA-F	CATTCTCAAAAAATCTACTACATTCTCAAAAAATCTACTA
BcMYB111-m-EMSA-R	TAGTAGATTTTTTGAGAATGTAGTAGATTTTTTGAGAATG
GFP-R	GTGCAGATGAACTTCAGGTCA
